# Structure‐Dependent Resonant Frequency Engineering of Textile Tactile Sensors Toward Rapid and Precise Braille Recognition Surpassing Human Sensation

**DOI:** 10.1002/advs.202520152

**Published:** 2026-01-12

**Authors:** Xianhong Zheng, Runrun Zhang, Yu Shi, Zhao Zhang, Guiyang Li, Zhengliang Shang, Xin Liu, Fan Zhao, Sheng Hu, Ran Xu, Shuai Wang, Zhiqi Zhao, Zhi Liu, Lihua Zou, Xu Han, Zongqian Wang, Wei Huang, Gengzhi Sun

**Affiliations:** ^1^ School of Textile and Garment Anhui Polytechnic University Wuhu China; ^2^ School of Flexible Electronics (Future Technologies) & Institute of Advanced Materials (IAM) Nanjing Tech University (Nanjing Tech) Nanjing China; ^3^ College of Intelligent Science and Control Engineering, Jinling Institute of Technology Nanjing China; ^4^ International Joint Laboratory of Green Textile (Zhejiang Sci‐Tech University) Ministry of Education Hangzhou China; ^5^ College of Intelligent Systems Science and Engineering Harbin Engineering University Harbin China; ^6^ Key Laboratory of Textile Science & Technology College of Textiles Donghua University Shanghai China; ^7^ Key Laboratory of Advanced Textile Materials and Manufacturing Technology Ministry of Education (Zhejiang Sci‐Tech University) Hangzhou China

**Keywords:** braille recognition, intelligent glove, sensory system, tactile sensor, textile

## Abstract

Artificial tactile perception emulating slow‐(SA) and fast‐adapting (FA) mechanoreceptors is crucial for visually impaired individuals as an advanced auxiliary learning electronic system. However, existing sensors, particularly single‐mode ones, struggle to simultaneously detect static pressure and high‐frequency vibrations due to their inherent response limitations. Herein, for the first time, we report a textile‐based bionic tactile sensor (TBTS) that, solely via piezoresistive mechanism, achieves high sensitivity and fast response across an ultrabroad frequency range (5–600 Hz), surpassing human vibrotactile range (<500 Hz). Finite element analysis (FEA) reveals that such superior capability originates from the resonant frequency engineering of 3D woven structure in sensing fabric. Under the assist of machine learning, an instantaneous braille‐to‐audio conversion system comprising a TBTS‐integrated commercial glove, signal processer and a smartphone interface is built, realizing rapid and precise braille recognition with an operational frequency significantly higher than skilled human reading speeds (5–10 Hz), achieving 100.0% accuracy for characters and 97.5% for Chinese multi‐character sentences, and enabling real‐time audio feedback. This work establishes a new paradigm for assistive technology, paving the way for next‐generation smart wearables that offer immediate aids in braille education and navigation.

## Graphical abstract

A resonant frequency engineering strategy is proposed to modulate the sensibility of piezoresistive textile‐based tactile sensor. It achieves simultaneous detection of static pressure and dynamic vibrations across an unprecedented bandwidth of 5–600 Hz, surpassing human sensation, therefore enables rapid and precise braille recognition. An instantaneous braille‐to‐audio conversion system is further built as learning aid for visually impaired individuals.

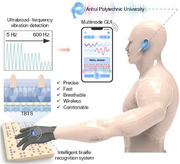

## 1 Introduction

Braille literacy is a critical determinant of life outcomes for the 285 million visually impaired individuals worldwide, with proficient users 80% more likely to secure skilled employment [1, 2]. However, it remains a formidable challenge to learn braille, particularly in developing regions where over 90% of visually impaired population live, due to the prohibitive cost of necessary equipment, insufficient support from government, and a critical scarcity of portable/wearable reading aids [3, 4, 5]. Wearable haptic systems that can convert braille to audio signals offer a promising solution, yet their development has been fundamentally constrained by the longstanding trade‐off: the inability of the tactile sensors to rapidly achieve simultaneous detection of static pressure and high‐frequency vibrations with high‐fidelity.

To date, research on braille recognition sensors has evolved along two mainstream pathways, both of which face inherent limitations in achieving rapid and precise signal acquisition of braille patterns. The first strategy employs single‐mode of either capacitive, piezoresistive, piezoelectric or triboelectric to fabricate sensors [6, 7, 8, 9, 10, 11, 12, 13, 14, 15, 16, 17, 18, 19, 20, 21, 22, 23]. While such designs benefit from structural simplicity, they are fundamentally ill‐suited for either static (piezoelectric and triboelectric) or dynamic (capacitive and piezoresistive) responsiveness. In addition, the homogeneous sensing layers suffer from limited signal fidelity and spatial resolution. Therefore, the inadequate sensibility of mimicking finger touch on and movement across braille texts impairs reading speed and accuracy. The second strategy relies on dual‐mode sensor design that integrates biomimetic slow‐adapting (SA) and fast‐adapting (FA) mechanoreceptors (e.g., combining piezoresistive and piezoelectric units) to decode both static and dynamic stimuli [24, 25, 26, 27, 28, 29, 30]. There are several drawbacks that fundamentally limit its practicality for rapid braille reading: (i) inevitable signal crosstalk between layered modules corrupts signal integrity, especially under dynamic scanning conditions; (ii) mechanical failure at the multiple interfaces of dissimilar materials compromises durability; (iii) complex data acquisition systems are required to process incompatible signal formats, increasing latency; and most critically, (iv) a fundamental bandwidth ceiling prevents effective replication of the full frequency range of human tactile perception (<500 Hz), making it impossible to accurately capture the high‐frequency vibrations essential for high‐speed braille recognition. Moreover, in such sensors, the typically used multi‐layered structures lack breathability, further hindering long‐term wearing comfort.

The imperative for high‐frequency response in tactile sensing is fundamentally rooted in the demands of high‐speed braille recognition. As a sensor slides over braille dots, the frequency (f) of the generated electrical signal is dictated by the scanning velocity (v) and the spatial period (λ) of the braille pattern (f ≈ v / λ). While the operational frequency for a skilled human reader is around 5–10 Hz, a machine‐assisted system aiming at surpassing human speed necessitates a superior dynamic response to capture these high‐frequency components, resulting in smeared signal profiles of the dots and consequently compromising both recognition speed and accuracy.

In this work, we propose a structure‐dependent resonant frequency engineering strategy to modulate the sensibility of textile‐based bionic tactile sensor (TBTS) that surprisingly achieves simultaneous detection of static pressure and dynamic vibrations across an unprecedented bandwidth of 5–600 Hz solely via piezoresistive mechanism toward rapid and precise braille recognition, surpassing human sensation (<500 Hz). TBTS leverages a meticulously 3D woven fabric functionalized with MXene/PEDOT:PSS to create a hierarchically responsive sensing layer. The structure‐dependent resonant behavior and high‐frequency responsiveness are validated by experimental characterizations and finite element analysis. It is noteworthy that TBTS represents the first report of such ultrabroad frequency detection capability in piezoresistive tactile sensors. Under the assist of machine learning, we further develop an instantaneous braille‐to‐audio conversion system comprising a TBTS‐integrated commercial glove, signal processer and a smartphone interface. This system achieves unparalleled accuracy (97.5%–100.0%) for characters/numbers/buttons and Chinese sentences, and shows the capability to deliver real‐time audio feedbacks. This work establishes a new paradigm for assistive technology, paving the way for next‐generation smart wearables that offer immediate aids in braille education and navigation.

## 2 Results and Discussion

**FIGURE 1:**
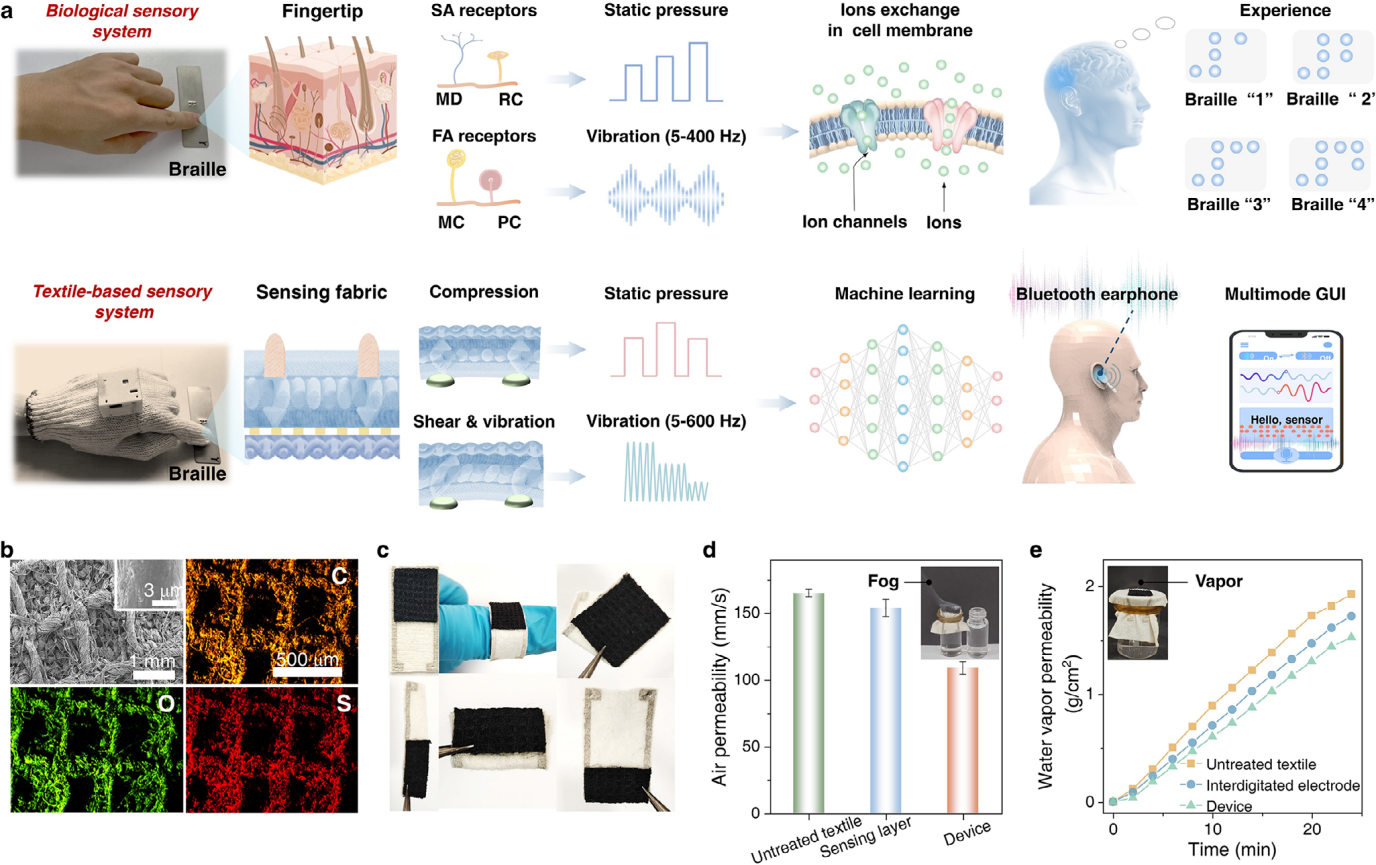
Schematic illustration of the TBTS‐based sensory system mimicking the human sensory system for braille‐to‐audio transduction. (a) The biological sensory system of humans and TBTS‐based sensory system. (b) SEM and EDS mapping images of the 3D sensing fabric. (c) Flexibility demonstrations of the TBTS. (d) Air permeability and (e) moisture permeability. Inset image showing the fog and vapor permeating through the TBTS.

In human biological sensory system (Figure 1a), Merkel discs (MD) are indeed located superficially at the epidermal‐dermal junction, Ruffini cylinder (RC) are situated in the deep dermis and subcutaneous tissue [31]. They act as SA receptors reside superficially within skin and generate sustained responses to static pressure stimuli, while Meissner corpuscle (MC) and Pacinian corpuscle (PC) as FA receptors produce transient responses only at the onset and offset of stimulation, enabling the detection of dynamic mechanical events such as vibration (5–400 Hz) and sliding motion [24]. These receptors can transduce external force stimuli into biopotentials via ion exchange and such non‐propagating electrical signals are subsequently transmitted by nervous system to brain. Such perceptual process enables visually impaired individuals to decipher the spatial arrangement and meaning of braille dots. Under this inspiration, we employ 3D woven fabric as the sensing layer to construct a textile‐based tactile sensor and develop an instantaneous braille‐to‐audio conversion system. The compressive, shear and interfacial vibration deformation in 3D woven structure empowers TBTS simultaneous detection of both static forces and high‐frequency vibrations solely through piezoresistive sensing mechanism, replicating the functionalities of SA and FA receptors. It is feasible to tune the resonant frequency of 3D fabric via tailoring woven structure and thus enhance the sensibility of as‐designed textile‐based tactile sensors. The collected distinct electrical signals are analyzed by a deep learning framework, enabling robust feature extraction and classification of braille patterns, wirelessly transmitted through a self‐designed signal processing circuit equipped with Bluetooth Low Energy (BLE), and vocally played on a smartphone via a multi‐mode graphical user interface (GUI). This system demonstrates three key technological advantages: (i) enhanced wearability with high breathability and moisture permeability, maintaining physiological comfort during prolonged use; (ii) Superior recognition accuracy achieved by applying deep learning techniques to extensive braille pattern datasets; and (iii) highly operational convenience through compact BLE modules, eliminating cable constraints. The intelligent braille recognition glove with all electronic components well‐integrated preserves the wearable characteristics as evidenced by photo images in Figure S1, and the circuit diagram is shown in Figure S2. The system employs a lithium‐ion battery to supply a 0.5 V bias to the sensor. The current variations during braille reading are subsequently processed by a transimpedance amplifier and an inverting amplifier for signal conditioning and amplification, transformed by an Analog‐to‐Digital converter, and wirelessly transmitted via BLE protocols.

TBTS is fabricated from silver interdigitated electrodes and a fabric sensing layer modified with MXene and PEDOT:PSS (Figure S3). The interdigitated electrodes were screen‐printed onto a cotton textile substrate using silver paste with tailored viscoelastic properties. These textile‐based interdigitated electrodes feature a low sheet resistance of 0.5 Ω cm^−1^ and high mechanical robustness under deformation, attributable to the strong silver‐textile adhesion promoted by polymer binders (Figures S4–S6). Both MXene and PEDOT:PSS form stable aqueous dispersions, as confirmed by the Tyndall effect (Figure S7). The sensing layer was fabricated through sequential spray coating of MXene and PEDOT:PSS dispersions onto a 3D woven fabric substrate with a honeycomb‐like microstructure (Figure S8). Scanning electron microscopy (SEM) images and corresponding energy‐dispersive X‐ray spectroscopy (EDS) mapping reveal uniform wrapping of the cotton yarns by MXene nanosheets and PEDOT:PSS (Figure 1b; Figures S9–S12), establishing a continuous conductive network.

As expected, benefiting from the intrinsic properties of textiles, TBTS exhibits remarkable flexibility (Figure 1c) and high permeability to both gas and moisture (Figure 1d,e; Figure S13). Gas permeability was visualized by substantial release of white fumes when covering TBTS on a bottle containing concentrated ammonia solution. Quantitative assessment further confirmed a gas permeability value of 109.0 ± 4.7 mm s^−1^ (Figure 1d), which is slightly lower than that of bare fabric and M2P2 sensing layer alone, due to the moderate blocking by the conductive coating and the laminated device architecture. Moreover, TBTS demonstrates an exceptional moisture permeability of 0.06 g cm^−2^ min^−1^ (Figure 1e) because of the high evaporation rate during test, implying its excellent vapor permeability and thus satisfying the requirements for effective thermoregulation during prolonged wear [32].

**FIGURE 2:**
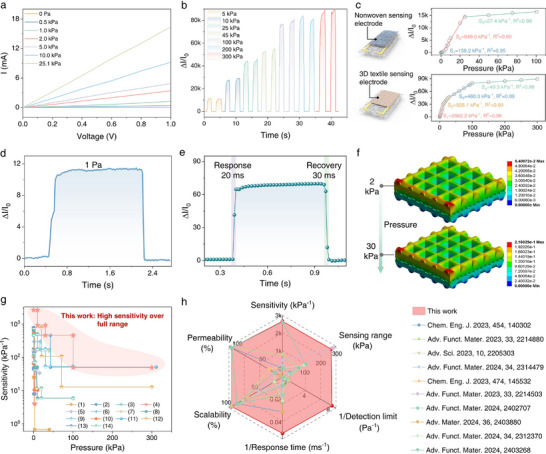
Static pressure sensing performance of TBTS. (a) *I‐*‐*V* curves of the sensor under various external pressures. (b) Relative current response at external pressures ranging from 5 to 300 kPa. (c) Schematic diagram showing the structure of sensor assembled by the nonwoven and 3D textile sensing electrode, and the corresponding sensing curves. (d) Current response at a static pressure of 1 Pa. (e) Response/recovery time. (f) Finite element modeling of the strain distribution of the sensing layer under 2 kPa and 30 kPa, respectively. (g) Comparison of the sensitivity and working range between our bionic sensor and other sensors made of (1) graphene/polydimethylsiloxane (PDMS) film [39], (2) wrinkled rGO/bond tape [40], (3) polypyrrole (PPy)/PDMS stamps film [41], (4) MXene/Au/polyethylene terephthalate (PET) film [42], (5) MXene/PDMS film [43], (6) MXene/protein nanofiber mat [44], (7) MXene/PEI nanofiber mat [34], (8) Ag nanowires/MXene aerogel [45], (9) MXene/Tissue paper [46], (10) MXene/bacterial cellulose/PDMS film [47], (11) intermittent structured MXene/PDMS film [33], (12) urchin‐like MXene/PDMS film [48], (13) MXene/AgNW nonwoven fabric [49], (14) MXene/AgNW fabric [50]. (h) Radar plot to comprehensively compare the pressure sensing performance of our bionic sensor with recently reported flexible pressure sensors, in terms of sensitivity, sensing range, detection limit, response time, scalability, and permeability.

To ensure high‐fidelity signal acquisition for rapid braille recognition, a customized low‐noise circuit was designed (Figure S2), and stable ohmic contact was secured between the interdigitated electrodes and the sensing fabric to minimize electronic noise and mechanical interference. Figure 2 shows the sensing performance of TBTS toward static pressure. Ohmic contact between sensing fabric and interdigitated electrode is evidenced by the linear current‐voltage (*I‐V*) characteristics; moreover, the slope increases with the exerted static pressures (Figure 2a), implying a decreased device resistance. The relative current change (∆*I*/*I*_0_) was monitored in a time‐resolved mode as a function of loaded pressures (Figure 2b); the continuous and stable increase demonstrates its capability for force sensing in a broad range from 5 to 300 kPa. To further substantiate the contribution of 3D woven structure on sensing performance, we optimized the conductive coating layer and also fabricated tactile sensors based on nonwoven sensing fabric for comparison. As shown in Figure 2c and Figure S14, M2P2‐based TBTS shows the optimal sensing performance, including significantly higher sensitivity (2662.2 kPa^−1^), which is 4‐fold greater than that of nonwoven sensor, and prominent response to 1 Pa with the predicted detection limit as low as 0.12 Pa (Figure 2d). This remarkable disparity underscores the critical role of the 3D woven architecture. Unlike the nonwoven fabric, which deforms minimally due to its dense, randomly‐oriented fiber network, the honeycomb structure provides ample void space for large, reversible compression. This leads to a dramatically higher change in the contact area within the conductive network and at the electrode interface, thereby translating into superior sensitivity. In addition, TBTS exhibits fast response time of 20 ms and recovery time 30 ms (Figure 2e), which is superior to most tactile sensors, such as bionic intermittent structured MXene/PDMS film‐based (54 ms) [33], MXene/PEI film‐based (163 ms) [34], MXene/nonwoven fabric‐based (61 ms) [35], and comparable with human skin (20–40 ms) (Table S2) [36, 37, 38], implying its potential capability for dynamic detection. Additionally, the TBTS exhibits good reliability over 4000 loading/unloading cycles with negligible signal fading and good washability because of the good adhesion between fiber and MXene/PEDOT:PSS (Figures S15 and S16).

Finite element modeling was employed to clarify the excellent pressure sensing performance of our TBTS (Figure 2f; Figure S17). When the loading pressure was raised from 2 to 30 kPa, the compression strain was gradually accumulated from 5.4% to 21.6%, resulting in enlarged contact area between 3D woven yarns in sensing fabric and interdigitated electrode, thereby producing continuous resistance variation and current responses. Therefore, the excellent pressure sensing performance of TBTS is attributed to the compressive deformation of 3D sensing fabric and the decreased interfacial contact resistance (Figures S18 and S19). The superiority of our TBTS is further confirmed by horizontally comparing the sensitivity and detectable range with previously reported MXene‐based and mictrostructured tactile and/or pressure sensors as illustrated in Figure 2g [33, 34, 39, 40, 41, 42, 43, 44, 45, 46, 47, 48, 49, 50, 51]. Specifically, the sensitivity of TBTS is 777.4 times higher than that of CNT/waterborne polyurethane‐based sensor [51], 477.5% higher than that of bionic intermittent structured MXene/PDMS film‐based pressure sensor [33], and 239.6% higher than that of urchin‐like MXene/polybutylene adipate‐polyurethane film‐based pressure sensor [48]. As shown in Figure 2h, a Radar plot was also constructed to comprehensively compare the performance of our device with other newly reported tactile sensors in terms of sensitivity, sensing range, detection limit, response time, scalability, and permeability (Table S1) [33, 34, 35, 49, 51, 52, 53, 54, 55, 56, 57]. Benefiting from their textile characteristics, compatibility with solution processing technique, simple device configuration, 3D woven patterns, and hierarchical microstructures, our TBTS exhibits outstanding overall performance and are feasible for wearable applications.

**FIGURE 3:**
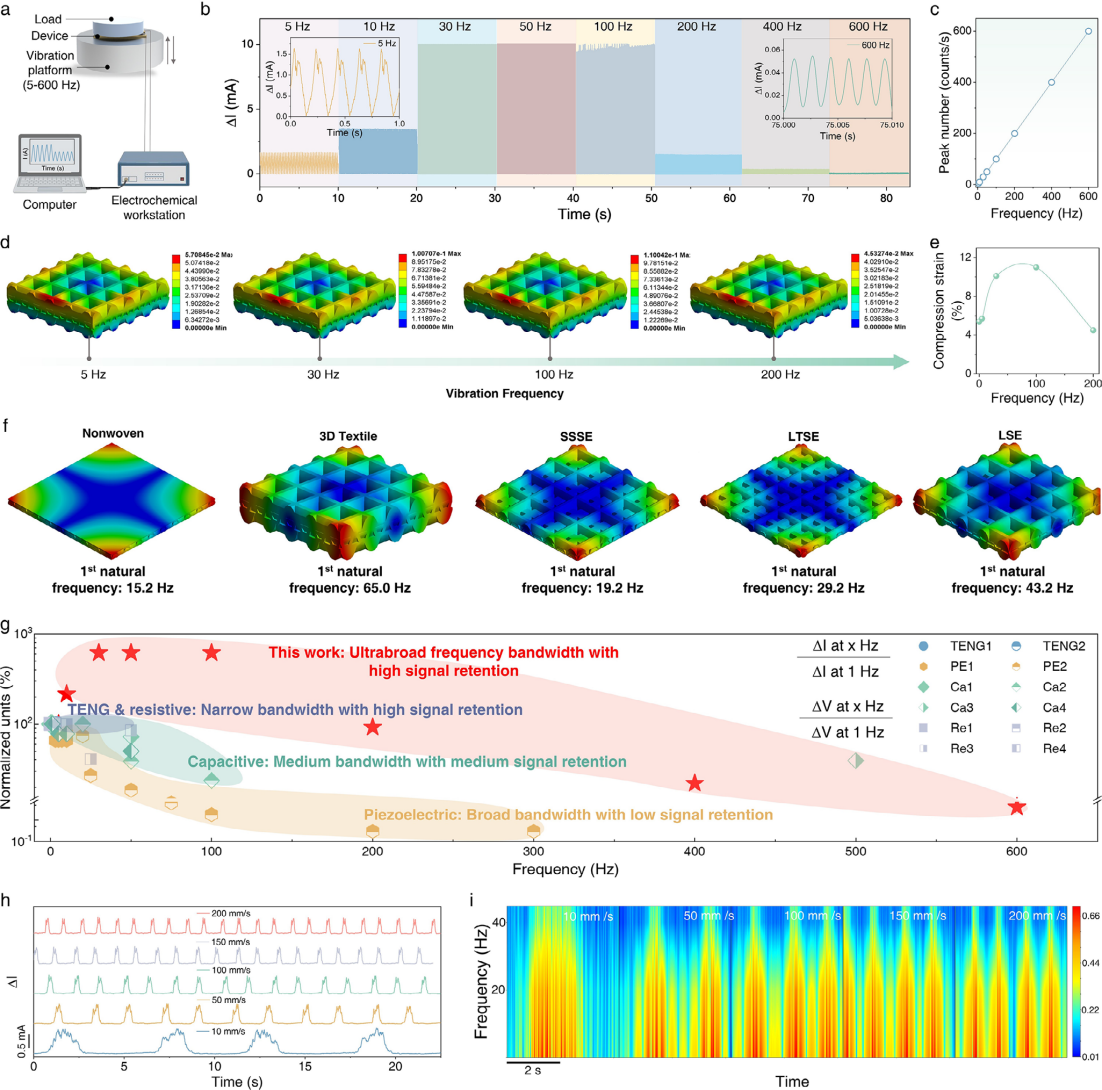
Vibration sensing capability of the TBTS. (a) Schematic diagram showing the measurement of vibration response behaviors. (b) Vibration‐response curves of sensor in the vibration range 5–600 Hz exceeds the human vibration detection range (<500 Hz). (c) Linear relationship between vibration frequency and peak number. (d) FEA modeling of compression strain distribution and (e) the compression strain value of the sensor under a dynamic force (2 kPa) with frequency of 5, 30, 100, and 200 Hz. (f) first natural frequency of various sensing electrode based on FEA method. (g) Comparison of the signal retention of our TBTS with reported tactile sensors based on resistive (RE)‐ [59, 60, 61, 62], triboelectric (TENG)‐ [27, 63], capacitive (CA)‐ [26, 64, 65], piezoelectric (PE)‐ [66, 67], based mechanisms. (h) Current response of TBTS sliding the braille “6” under various speed and (i) the corresponding frequency spectra.

We next evaluated the high‐frequency vibration response of the TBTS, which is a critical determinant of its performance in high‐speed braille recognition. As the sensor's operational frequency during scanning is proportional to the sliding speed, a broad bandwidth is essential to faithfully capture the temporal shape of the braille‐induced signals without distortion at elevated velocities. Figure 3 shows the high‐frequency response of TBTS by placing the device on a vibration stage. The corresponding signals were collected as a function of vibration frequency, while the applied pressure was maintained constant (Figure 3a). TBTS gives distinct current changes in an ultrabroad frequency range spanning from 5 to 600 Hz (Figure 3b; Figures S20 and S21), and the responding signal (peak number) varied linearly with vibration frequency (Figure 3c), surpassing the sensibility of human skin (<500 Hz) [58]. Notably, the strength of responding signal shows obvious dependence on frequency (Figure 3b) similar to the resonant behavior and retains at a high level within 30–100 Hz. Finite element analysis (FEA) was performed to probe the distinctive behavior of TBTS to vibration and the results are presented in Figure 3d,e. Figure 3d displays the compressive strain distribution of the 3D woven sensing fabric under 2 kPa across the frequency range of 5–200 Hz (Figure 3e; Figure S22). When the vibration frequency increased from 5 to 30 Hz, the compressive strain gradually increased. Within 30–100 Hz, the compressive strain remained at a high plateau, reaching 10% and 11%, respectively. Beyond 100 Hz, the compressive strain dropped sharply. This indicates that the resonant frequency of the device lies within 30–100 Hz, coinciding with our experimental observation (Figure 3b), therefore leads to the maximum mechanical deformation and the largest current change. To further validate our hypothesis, a free modal FEA was performed on sensing fabrics with different woven configurations (Figure 3f). The 3D woven fabric sensing layer exhibits a higher first‐order natural frequency of 65.0 Hz compared to the nonwoven one (15.2 Hz), indicating its superior high‐frequency response characteristics. Moreover, the structure‐dependent resonant behavior was further highlighted by evaluating the natural frequencies of different woven configurations, such as the single‐side sensing electrode (SSSE), low‐thickness sensing electrode (LTSE), and large sensing electrode (LSE). The results confirm that the natural frequency severely depends on structural parameters (Figure S23) and suggests the high‐frequency response of TBTS can be tailored by adjusting the woven patterns. Therefore, the observed decline in signal amplitude beyond 100 Hz for TBTS is a direct consequence of the system's dynamics. As the driving frequency moves progressively higher than the structure's first natural frequency (∼65 Hz), the inertial mass of the fabric prevents it from fully complying with the external excitation. This leads to a rapid attenuation of the achievable compressive strain, which in turn causes the corresponding drop in electrical response. This behavior is characteristic of a damped harmonic oscillator and confirms that the sensor's frequency response is governed by its engineered mechanical properties.

To demonstrate the potential of our TBTS for braille recognition, we compared its operational frequency with that of a proficient braille reader (reading speed: ∼5 characters s^−1^ [1]). The recognition frequency for a skilled human reader falls in the range of 5–10 Hz (see calculation in the Supporting Information), which is substantially lower than the natural frequency of TBTS (65.0 Hz). This notable disparity highlights the significant potential and competitive advantage of TBTS for high‐speed braille recognition beyond human perception. To quantitatively evaluate the vibration‐detection performance of our TBTS, we introduce a normalized unit (Nu = Δ*I*_at x Hz_/Δ*I*_at 1 Hz_ or Δ*V*_at x Hz_/Δ*V*_at 1 Hz_). TBTS exhibits high signal retention across a broad frequency range (Figure 3g), outperforming most previously reported tactile sensors based on triboelectric, piezoresistive, piezoelectric, or capacitive mechanism [26, 27, 59, 60, 61, 62, 63, 64, 65, 66, 67]. The high signal retention across an ultrabroad frequency range and the comparison with other mechanisms of TBTS can be attributed not only to the resonant behavior but also to the effective damping of spurious mechanical vibrations. The viscoelastic polymer matrix within the 3D woven fabric introduces intrinsic material damping, which broadens the resonance peak and suppresses high‐frequency mechanical noise beyond the operational bandwidth, thereby contributing to the stable and distinguishable signals even at frequencies far from the resonance. Additionally, our sensor surpasses existing dual‐mode counterparts by achieving ultrabroad‐band vibration perception solely through a single piezoresistive mechanism. This streamlined approach enables a simpler device architecture, fewer signal acquisition circuits, less signal crosstalk, and superior signal integrity (Table S4).

A potential conceptual consideration is the role of the viscoelastic polymer‐based materials in our resonant frequency engineering strategy. In classical structural dynamics, homogeneous isotropic materials (e.g., metals) possess a well‐defined Young's modulus, leading to a straightforward calculation of natural frequency. In contrast, the viscoelastic polymers used in our textile structure exhibit a frequency‐dependent complex modulus. However, this does not invalidate the concept of structural resonance for our system. On the contrary, for a defined architecture under small‐amplitude vibrations, the structure exhibits a dominant resonant mode, the frequency of which is effectively engineered by its geometric design, as unequivocally demonstrated by our FEA (Figure 3f). We further examined the response fidelity of our TBTS by scanning over a braille character of “6” using a robotic prosthesis. Tests were conducted at constant speeds of 10, 50, 100, 150, and 200 mm s^−1^. Clearly discernible and well‐defined electrical signals were consistently obtained even at the speed of 200 mm s^−1^ (Figure 3h,i; Figure S24), which exceeds the reading speeds typically used by visually impaired persons (15–60 mm s^−1^) [68].

**FIGURE 4:**
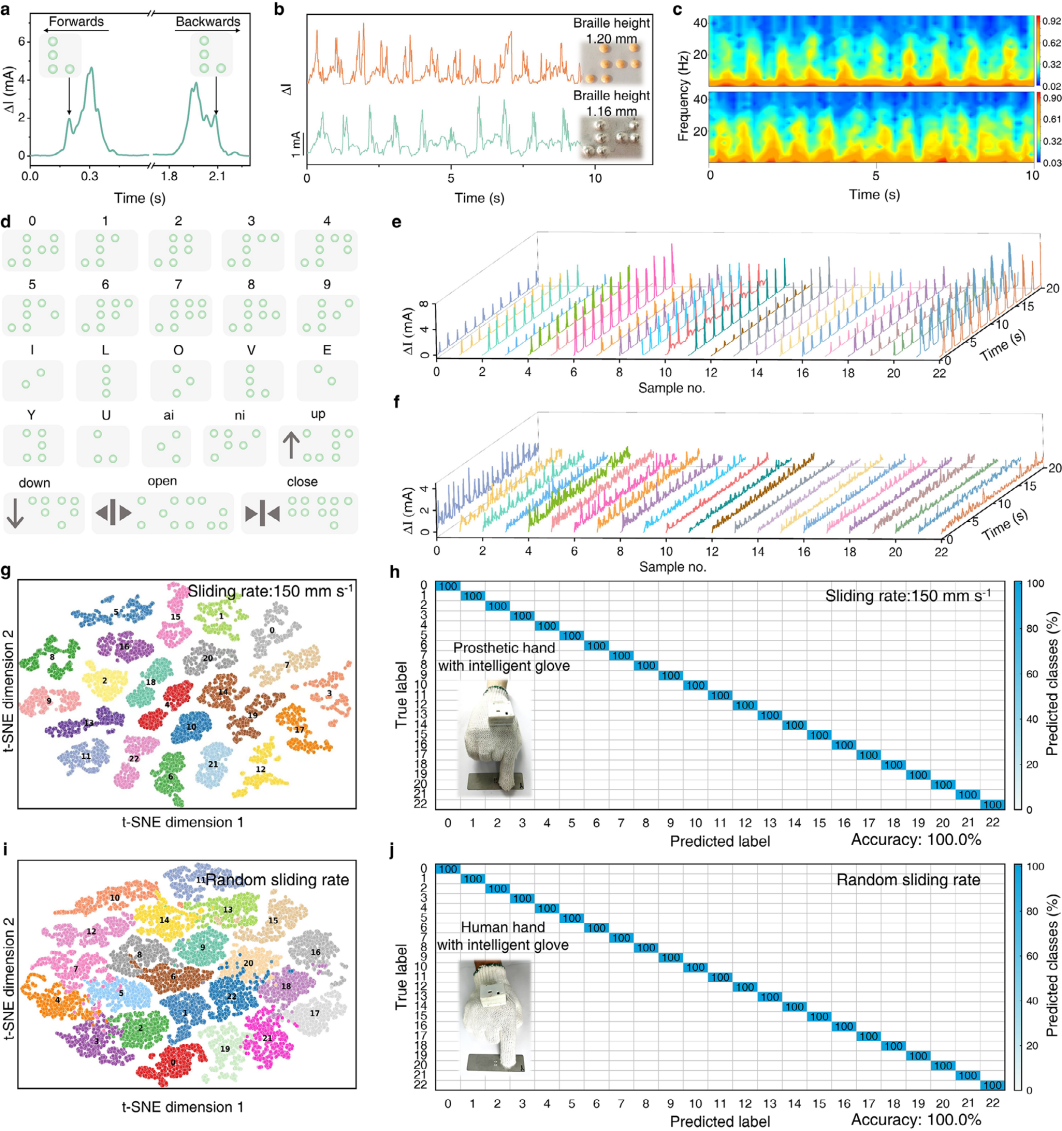
Recognition of braille characters/numbers/buttons using the TBTS. (a) The relative current response of the sensor scanning over the braille “V” backward and forward at 150 mm s^−^ [1]. (b) Current response and (c) frequency‐domain signals obtained using wavelet transform of the TBTS when scanning the braille “0” with different braille height. (d) Images of 23 customized braille. Time‐domain signals of 23 customized braille sensed using the TBTS at sliding rate of (e) 150 mm s^−^ [1] and (f) random sliding rate. (g) T‐SNE visualization and (h) confusion matrix for the recognition of 23 customized braille at a sliding rate of 150 mm s^−^ [1]. (i) T‐SNE visualization and (j) confusion matrix for the recognition of 23 customized braille at a random sliding rate.

As a proof‐of‐concept demonstration, TBTS was used for recognizing braille based on its excellent detecting capability of static pressure and high‐frequency vibration. Notably, the detection of braille character “V” at 150 mm s^−1^ reveals direction‐dependent current response profiles (Figure 4a) with prominent variation in peak position during forward and backward scanning; therefore, the implementation of reciprocating scanning protocols is necessitated for comprehensive spatial pattern resolution. The capability of our TBTS for height discrimination was quantified using braille specimens of numeral “0” with 1.16 and 1.20 mm dot protrusions (Figure 4b). While almost maintaining consistent temporal response profiles, TBTS produced signal strength proportional to dot height. It is noted that the wavelet transform‐based frequency domain preserved the characteristic spectral signatures regardless of the braille dot height (Figure 4c), indicating the spatial arrangement is the primary decoding criterion rather than dimensional parameters. TBTS was further systematically evaluated by sliding over 23 customized braille (dot height: 1.16 mm) and 19 homemade braille (dot height: 1.20 mm) respectively representing numeric, alphabetic and elevator symbols under either constant (150 mm s^−1^) or random rates (Figure 4d; Figures S25 and S26). The recorded real‐time signals demonstrate distinguishable signatures for all braille configurations no matter what sliding rate and dot height were used (Figure 4e,f; Figures S27–S32). Complementary wavelet transformation of the acquired signals enables the extraction of characteristic frequency‐domain features (Figures S33–S35). A machine learning framework by combining feature extraction with dimensionality reduction is implemented to address the inherent complexity of output signals. High‐dimensional data were projected into a 2D visualization space to effectively preserve both global and local structural relationships by using t‐distributed stochastic neighbor embedding (t‐SNE).

As demonstrated in Figure 4g, the t‐SNE plot exhibits well‐segregated clusters corresponding to different braille patterns, with complete separation maintained even under randomized sliding conditions. The confusion matrix confirms exceptional classification accuracy (100.0%) across all 23 customized and 19 homemade braille symbols (Figure 4h) is achieved because of three key design elements: (i) standardized tactile datasets generated under controlled sliding rates minimize signal variance; (ii) ultrabroad frequency vibration detection capability, and (iii) the optimized sensing performance, particularly its high sensitivity and rapid response, enables accurate discrimination of subtle braille feature differences. To validate practical applicability, we mounted a TBTS on the index finger of a man. Despite the pressure and velocity vary during sliding, both cluster separation in t‐SNE space and 100.0% recognition accuracy are still achievable (Figure 4i,j; Figures S36–S40 and Video S1). This robustness to operational variability confirms the reliability of our TBTS in real‐world assistive applications for visually impaired individuals.

**FIGURE 5:**
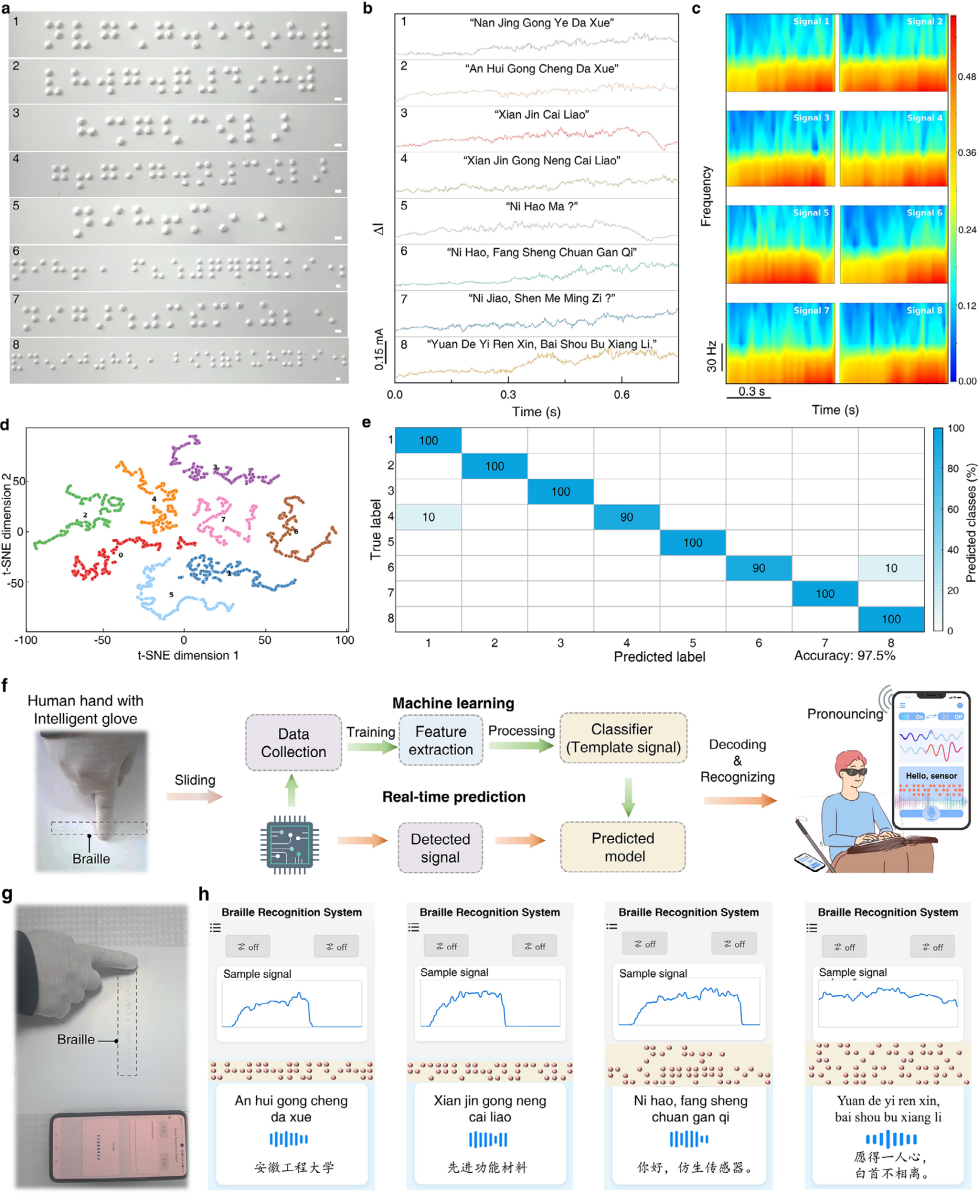
Recognition of braille sentences and conversion to audio feedbacks. (a) Image of 8 Chinese braille sentences. Scale bar: 1.5 mm. (b) Current response curves and (c) time‐frequency analysis comparison of the TBTS toward 8 braille sentences. (d) T‐SNE visualization and (e) confusion matrix for the recognition of the 8 braille sentences at a random sliding rate. (f) Structure diagram of the intelligent braille recognition system. (g) Photo and (h) demonstration of the real‐time braille‐to‐audio transduction of Chinese sentences using the intelligent braille recognition system at random sliding rates.

In contrast to the alphanumeric system of English braille, the combination of initials and finals makes Chinese braille more difficult. Additionally, compared to that on rigid substrates, the recognition on conventional braille paper presents greater challenges primarily due to its softness accompanying with possible mechanical deformation induced by sliding. As the dynamic interaction between sensor and compliant paper may alter the original dot morphology beyond standardized specifications, such distortion significantly compromises the accuracy of character recognition. According to the experimental results discussed in Figure 5, it is reasonable to speculate that our TBTS has the power for quick and accurate recognition of Chinese braille. We conducted randomized trials on eight Chinese braille sentences (4–10 characters long) as shown in Figure 5a to evaluate the practicality of our TBTS. Distinguishable time‐domain signals were obtained (Figure 5b) and these time‐domain signals were further processed using wavelet transform for time‐frequency analysis (Figure 5c, Figures S29–S31). This advanced signal processing technique serves as a powerful software filter, effectively isolating the characteristic frequency features of braille dots from the background electronic noise, thereby enhancing the signal‐to‐noise ratio prior to classification. The implemented RSNet architecture further incorporates a shrinkage module that performs adaptive denoising, reinforcing the model's robustness against residual signal fluctuations. We employed t‐SNE to classify these signals for dimensionality reduction and the 2D projection revealed well‐separated clusters for all eight sentences (Figure 5d), demonstrating the effectiveness of this method for feature discrimination. Subsequent confusion matrix analysis confirmed an average classification accuracy of 97.5% (Figure 5e). Importantly, our TBTS demonstrated overall performance improvements in terms of recognition accuracy, scanning rate, character numbers, and sentence length (Table S3) [6, 7, 8, 9, 10, 11, 30, 69, 70], compared with the reported braille recognition devices involving piezoresistive, capacitive, piezoelectric, triboelectric, or their combined dual‐mode mechanisms, such as piezoresistive/piezoelectric dual‐mode tactile sensor (accuracy: 77.5–90.6%, scanning rate<17.0 mm s^−^ [1]) [30], and triboelectric sensor (accuracy: 96.1%) [6].

We further developed an intelligent braille recognition system that enables real‐time braille‐to‐audio conversion by wirelessly integrating TBTS with machine learning algorithms, signal processing modules, and a smartphone interface. This system provides multimodal feedback (audio and visual) through a user‐friendly smartphone application, offering an effective tool to assist visually impaired individuals in braille literacy and communication. The system comprises four key components: (i) a sensor‐embedded glove for tactile detection, (ii) a customized signal acquisition module that processes braille sensing data and transmits it wirelessly via Bluetooth to a mobile device, (iii) the above‐described machine learning framework for braille pattern recognition, and (iv) a multimodal output interface providing simultaneous text and audio feedback (Figure 5f). In the operational workflow, the TBTS initially collects training data through randomized sliding across braille surfaces. Subsequent machine learning employs feature extraction algorithms to identify discriminative spatial‐temporal patterns in the obtained data, establishing optimized classification templates through supervised learning. During operation, continuous tactile signals acquired through dynamic scanning undergo systematic comparison with these pre‐trained templates using adaptive pattern matching algorithms, enabling accurate character‐level braille identification. This hierarchical recognition architecture combines statistical learning with dynamic signal processing to achieve both robustness and rapid recognition. As illustrated in Figure 5g, the system demonstrates efficient braille decoding through random scanning, with recognition results instantaneously displayed as on‐screen text and audibly delivered through smartphone speakers. Comprehensive testing confirmed the capability of our system to accurately recognize and vocalize complete braille sentences (Figure 5h; Figure S41 and Video S2). This performance demonstrates the practical applicability of our intelligent braille recognition system in enabling real‐time access to braille information for visually impaired users, significantly enhancing their ability to obtain textual/audible information and improve quality of life through assistive technology.

## 3 Conclusions

In summary, we have developed a high‐performance textile‐based tactile sensor and modulated its sensibility through structure‐dependent resonant frequency engineering strategy. Solely relying on piezoresistive mechanism, tailoring 3D woven structure can tune the resonant frequency of sensing fabric and thus endows TBTS the ability to precisely record both static pressure and high‐frequency vibrations with an ultrabroad detectable bandwidth of 5–600 Hz, surpassing human vibrotactile sensation (<500 Hz) and overcoming the long‐standing limitation of single‐mode sensors. As a consequence, our TBTS exhibits faster braille recognition than that of skilled human reader (5–10 Hz). Furthermore, we integrate TBTS into an intelligent braille recognition system combining machine learning, signal processing, and a smartphone interface, achieving exceptional accuracy in recognizing 23 braille characters/numbers/buttons (accuracy: 100.0%), and even 8 complex Chinese sentences (accuracy: 97.5%). With embedded processing enabling real‐time audio feedback, the system offers a significant advancement over existing braille reading aids. This work demonstrates a successful fusion of flexible textile electronics, adaptive algorithms and user‐centered design, establishing a new paradigm for high‐performance assistive technology. It provides an effective platform for braille education and navigation, and opens promising avenues for future accessible wearables with enhanced tactile perception capabilities.

## 4 Experimental Section

### 4.1 Preparation of Textile Bionic Tactile Sensor

The textile sensor was composed of interdigitated electrode and sensing layer. The textile‐based interdigitated electrodes were fabricated via a commercial screen‐printing method as described in our previous reports [38, 50]. In detail, the commercial silver paste (80 wt%) was screen‐printed on the cellulose nonwoven fabric to form the interdigitated electrodes with a size of 13 mm × 30 mm. Specially, the electrode width and gap were set as 1.0 mm and 0.5 mm, respectively. The sensing fabrics were fabricated via a continuous spray‐coating method. Cotton honeycomb fabric (honeycomb size: 1.5 mm × 1.5 mm) was used as the scaffold. Prior to coating conductive materials, fabrics were first desized to increase the fabric hydrophilicity in a mixture solution of 10 g L^−^ [1] NaOH and 1 g L^−^ [1] fatty alcohol polyoxyethylene ether in the bath ratio of 1:30.10 mg mL^−^ [1] MXene dispersion was spray‐coated on the desized honeycomb fabric, and then dried at 50°C for 10 min. The obtained fabric was labeled as M1. Subsequently, 10 mg mL^−^ [1] PEDOT:PSS dispersion was then spray‐coated on the surface of M1 following the same procedure of MXene coating, and the obtained fabric was labeled as M1P1. To further increase the conductivity of composite fabrics, MXene and PEDOT:PSS was continuously spray‐coated on the M1P1, respectively, and the obtained fabrics were labeled as M2P1 and M2P2, respectively. The fabricated silver‐based interdigitated electrode and bionic sensing electrode were adhered together using double‐side tape to form the pressure sensor.

### 4.2 Finite Element Analysis (FEA) Simulation

FEA was implemented by using commercial package ANSYS workbench. The unit cell of honeycomb fabric was simplified into a 3D grid, and the structure model was built using SOLIDWORKS software (Figure S42). The structure parameters were set to be consistent with its actual values (length: 1.5 mm, width: 1.5 mm, height: 1.3 mm, wall thickness: 0.24 mm). The constructed model was then imported into the ANSYS Workbench, and the parameters were also set according to their actual values. A load of 10, 20 and 30 kPa were applied on the upper surface of the fabric, and a displacement constraint was added on the lower surface. Eventually, a contour map of strain distribution can be obtained. Additionally, by applying a strain of 10% on the fabric surface, a stress distribution contour map can be obtained.

### 4.3 Data Collection of Braille Signals

A variety of braille signs, including braille numbers (0–9), English alphabet (I, L, O, V, E, Y, U), Chinese Pinyin (“ai”, “ni”), and elevator braille (“up”, “down”, “open”, and “close”) were used for recognition. In addition, 8 Chinese braille sentences, including “Nan Jing Gong Ye Da Xue”, “An Hui Gong Cheng Da Xue”, “Xian Jin Cai Liao”, “Xian Jin Gong Neng Cai Liao”, “Ni Hao Ma?”, “Ni Hao, Fang Sheng Chuan Gan Qi”, “Ni Jiao Shen Me Ming Zi?”, “Yuan De Yi Ren Xin, Bai Shou Bu Xiang Li” were also used for recognition. Data collection of braille signals was involved in two cases: (i) attaching the sensor on a prosthetic hand and sliding on braille signs under constant speed and (ii) attaching the sensor on a participant finger and sliding on braille signs under random speed. During sliding, the current response was collected at a frequency of 1000 Hz. It should be noted that a constant loading pressure (6.5 kPa) is essential for mimicking the process of fingertip reading braille and providing a stable mechanical boundary condition, minimizing vibration noise induced by inconsistent sensor‐text interactions. The signal acquisition system was built around a low‐noise design, utilizing precision amplifiers in the signal conditioning circuit to minimize electronic noise floor optimizing the physical contact between the interdigitated electrodes and the sensing electrode.

### 4.4 Machine Learning for Braille Recognition

A sensor moved back and forth over braille signs to collect enough data for machine learning. These data were then processed through machine learning and training to achieve recognition and prediction. Specifically, an improved Residual Network (RSNet) based on 1D convolution was employed for classifying braille labels. The overall framework diagram (Figure S43) illustrates the RSNet structure. The network begins with an initial convolutional layer (Conv1) that expands the input from 1 channel to 16 channels, followed by four residual blocks (Resblock1, Resblock2, Resblock3, Resblock4) with channel dimensions of 64, 128, 256, and 512, respectively. Each residual blocks incorporates a Shrinkage module that implements soft‐thresholding denoising through adaptive average pooling and a two‐layer fully connected network with batch normalization and sigmoid activation. The network concludes with global average pooling and a fully connected layer that outputs logits for 23 braille classes. Additionally, we use a sliding window technique to segment the time‐series data into fixed‐length segments (512 data points) for real‐time dynamic recognition. To evaluate the model's performance, we conducted multiple experiments on the validation dataset, calculated the accuracy and loss values, and plotted the loss and accuracy curves during training. Furthermore, we used confusion matrices to display the model's predictions for each category and employed t‐SNE to reduce and visualize intermediate features to observe the distribution of features across different categories.

### 4.5 Construction of the Intelligent Braille Recognition System for Real‐Time Braille‐to‐Audio Transduction

The intelligent glove was composed of a tactile sensor, STM32F03C8T6 microcontroller minimum system board, transimpedance amplifier, and inverter. The real‐time data, obtained from sliding the sensors back and forth across the braille sign surface, was compared with a machine‐learning model designed for braille recognition. The recognition and prediction results are not only displayed via Bluetooth on mobile phone or computer app GUI, but also presented in audio format. This GUI featured real‐time current response curves, template current response curves, an image of the sample, and actual meaning of braille sign. The recognition experiments were conducted on a laptop with 16 GB of RAM. Importantly, the signal collection frequency was maintained at 1000 Hz or higher to capture detailed textures.

### 4.6 Ethical Statement

All experiments were performed in accordance with all local laws and approved all relevant ethics bodies. Informed signed consent was received from the participants of the experiments with the wearable sensor devices.

## Conflicts of Interest

The authors declare no conflicts of interest.

## Supporting information




**Supporting File 1**: advs73428‐sup‐0001‐SuppMat.docx.

**Supporting File 2**: advs73428‐sup‐0002‐VideoS1.mp4.

**Supporting File 3**: advs73428‐sup‐0003‐VideoS2.mp4.

## Data Availability

The data that support the findings of this study are available from the corresponding author upon reasonable request.
