# Supplementary material for: Structure‐Dependent Resonant Frequency Engineering of Textile Tactile Sensors Toward Rapid and Precise Braille Recognition Surpassing Human Sensation
Source: Adv Sci (Weinh). 2026 Jan 12;13(13):e20152. doi: 10.1002/advs.202520152 (PMC12955891; doi:10.1002/advs.202520152)
Supplement: Supplementary file 1 — Supporting File 1: advs73428‐sup‐0001‐SuppMat.docx. [file ADVS-13-e20152-s001.docx]

**Supporting Information**

**Structure-Dependent Resonant Frequency Engineering of Textile Tactile Sensors towards Rapid and Precise Braille Recognition Surpassing Human Sensation**

*Xianhong Zheng,*^#,^* *Runrun Zhang,*^#^ *Yu Shi,*^#^ *Zhao Zhang,*^#^ *Guiyang Li, Zhengliang Shang, Xin Liu, Fan Zhao, Sheng Hu, Ran Xu, Shuai Wang, Zhiqi Zhao, Zhi Liu, Lihua Zou, Xu Han, Zongqian Wang, Wei Huang,** *Gengzhi Sun**

Xianhong Zheng, Guiyang Li, Shuai Wang, Zhiqi Zhao, Zhi Liu, Lihua Zou, Xu Han, Zongqian Wang

School of Textile and Garment, Anhui Polytechnic University, Wuhu 241000, China

E-mail: zhengxianhong@ahpu.edu.cn

Runrun Zhang, Yu Shi, Wei Huang, Gengzhi Sun

School of Flexible Electronics (Future Technologies) & Institute of Advanced Materials (IAM), Nanjing Tech University (Nanjing Tech), Nanjing 211816, China

E-mail: vc@nwpu.edu.cn; iamgzsun@njtech.edu.cn

Zhao Zhang, Zhengliang Shang

College of Intelligent Science and Control Engineering, Jinling Institute of Technology, Nanjing 211199, China

Xianhong Zheng

International Joint Laboratory of Green Textile (Zhejiang Sci-Tech University), Ministry of Education, Hangzhou, 310018, China

Xin Liu

College of Intelligent Systems Science and Engineering, Harbin Engineering University, Harbin 150001, China

Fan Zhao, Sheng Hu, Ran Xu

Key Laboratory of Textile Science & Technology, College of Textiles, Donghua University, Shanghai 201620, China

Xianhong Zheng

Key Laboratory of Advanced Textile Materials and Manufacturing Technology, Ministry of Education (Zhejiang Sci-Tech University), Hangzhou, 310018, China

^#^These authors contribute equally to this work.

# Experimental Section

## Materials

Lithium fluoride (LiF, 99.99 wt%) was purchased from Zhengzhou Feynman Biotechnology Co., Ltd (Zhengzhou, China). Hydrochloric acid and ethanol (99.7 wt%) were provided by Sinopharm Chemical Reagent Co., Ltd., China. Silver paste was provided by Hong Kong Mechanic Co., Ltd (Hong Kong, China). Ti_3_AlC_2_ MAX was obtained from 11 Technology Co., Ltd. PEDOT:PSS (Clevios PH1000) was purchased from Wuhan Zhuojia Technology Co., Ltd (Wuhan, China). Honeycomb cotton fabric with different specifications was provided by Changyi Huawei Fabric Co., Ltd., China. All of the chemicals were used as received without further purification.

## Characterizations

The X-ray diffraction (XRD) patterns were characterized by SmartLab SE. The surface morphology of the electrodes was observed using a field emission scanning electron microscope (SEM, CIQTEK3200A and HITACHI S4800). The gas permeability of fabrics was measured via a YG461E-Ⅱ gas permeability instrument, following the standard of GB5453–1997. The sheet resistance of the sensing electrode was measured by the CXT2663 four-point probe meter. The 3D morphology of the sensing electrode was observed via a KEYENCE VHX-970 3D ultra-depth field microscope. The pressure sensing performance of sensors was characterized using a home-made testing system, containing a force meter and electrochemical sensing analyzer. The sensitivity is defined as S=(Δ*I*/*I*_0_)/Δ*P*, where *I*_0_, Δ*I*, and Δ*P* is the current without external pressure, relative change of current, and external pressure, respectively.

## Calculation of braille reading frequency for blind people

Consider a proficient Braille reader scanning text at a rate of **5 characters per second (5 Hz)**.

**• Scenario 1: Single-Dot Character**

The simplest character, such as the letter 'a' (⠁), consists of a single raised dot. As the finger glides over this dot, it completes one full cycle of mechanical stimulation (contact, peak pressure, and release). The fundamental frequency of this tactile event cycle corresponds directly to the character scanning rate, which is **5 Hz**.

**• Scenario 2: Multi-Dot Character and Higher-Frequency Components**

A more complex character, such as 'f' (⠋), features three vertically aligned dots.

When a finger moves at a constant speed across this character, it encounters these three dots in rapid succession.

This process can be described as a superposition of a faster "dot-passing frequency" onto the slower 5 Hz "fundamental character frequency."

Calculation of the Dot-Passing Frequency:

Assuming a standard Braille dot diameter of ~1 mm and a center-to-center dot spacing of ~2.5 mm, the finger's scanning velocity is calculated as follows:

5 characters/s × 2.5 mm/character = 12.5 mm/s.

For a character like 'f' with dots in a column, the time interval to pass over all three dots is:

(Spacing between first and last dot) / Velocity = (2.5 mm × 2) / 12.5 mm/s = 0.4 seconds. Over a duration of 0.4 seconds, three events occur. This yields an average event rate of 3 events / 0.4 seconds = 7.5 events per second, which is equivalent to 7.5 Hz.

Therefore, while the primary frequency component of the tactile signal generated at a scanning rate of 5 characters per second lies within the 5-10 Hz range, the signal contains crucial higher-frequency information that can extend to tens or even hundreds of Hertz. The human fingertip, acting as a biological high-pass filter, is exquisitely sensitive to these high-frequency vibrations, which are critical for precise texture and pattern discrimination.


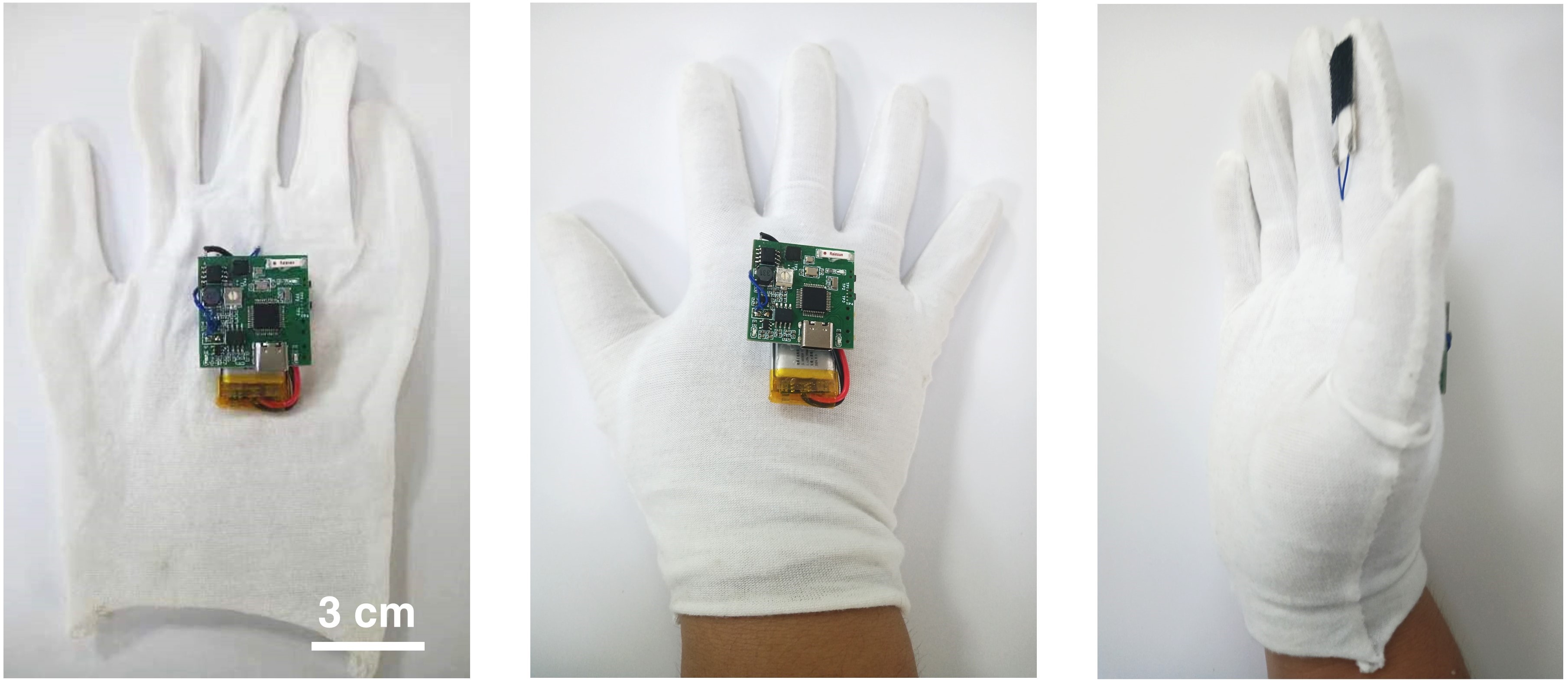


**Figure S1.** Wearable demonstrations of the intelligent braille recognition system.


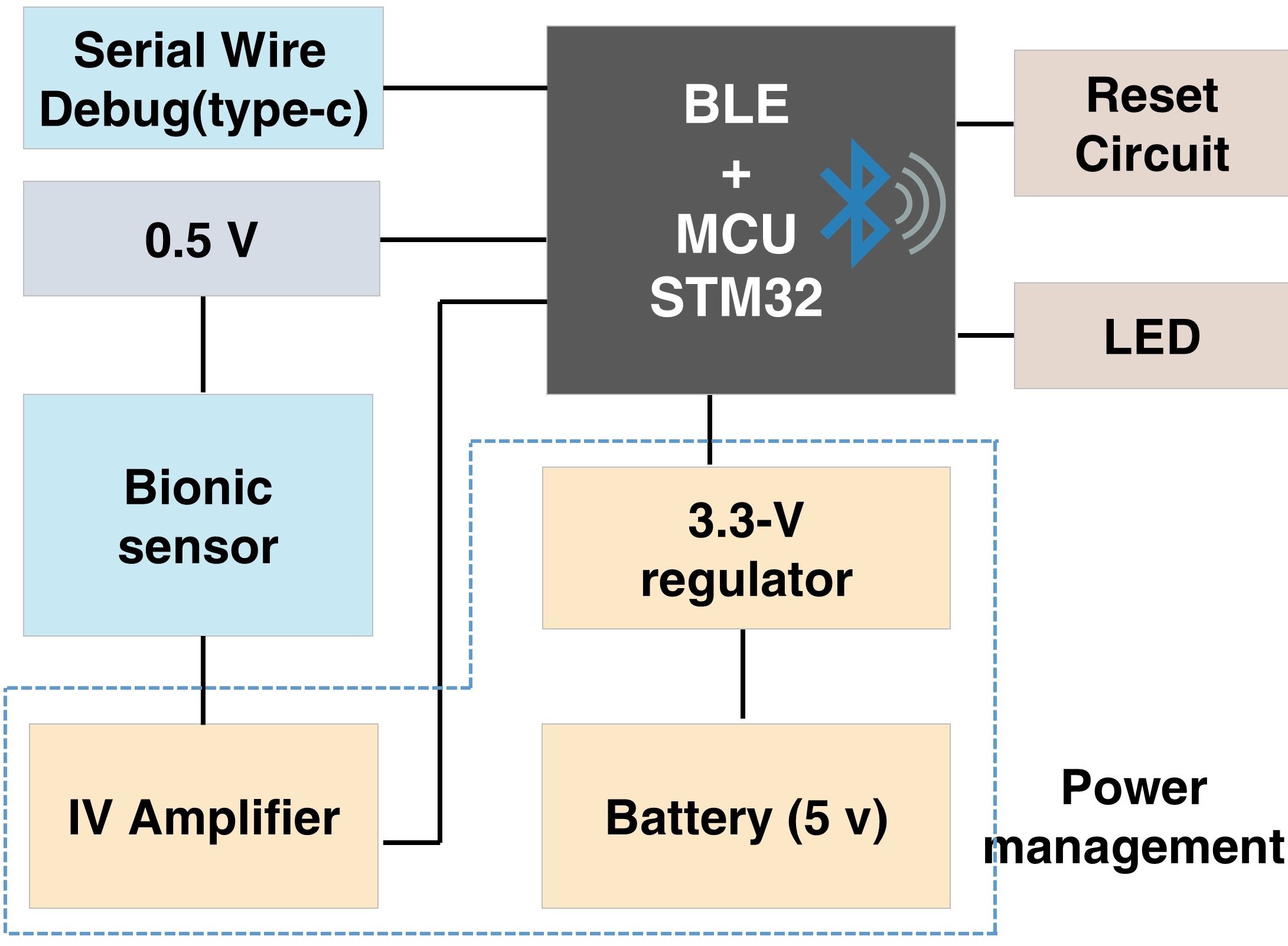


**Figure S2.** Brief block diagram of the intelligent braille recognition system.


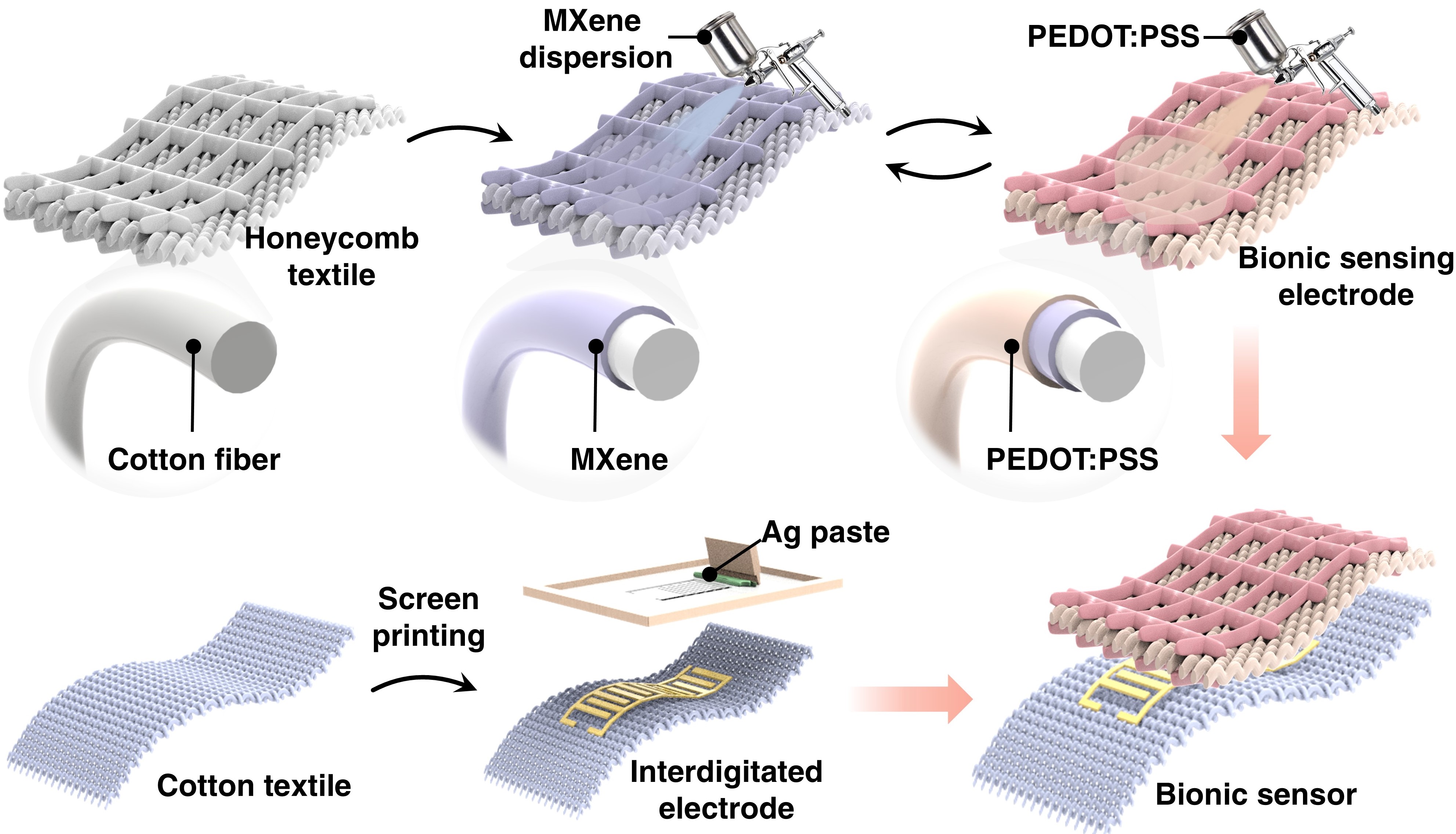


**Figure S3.** Schematic diagram of the fabrication of the TBTS.


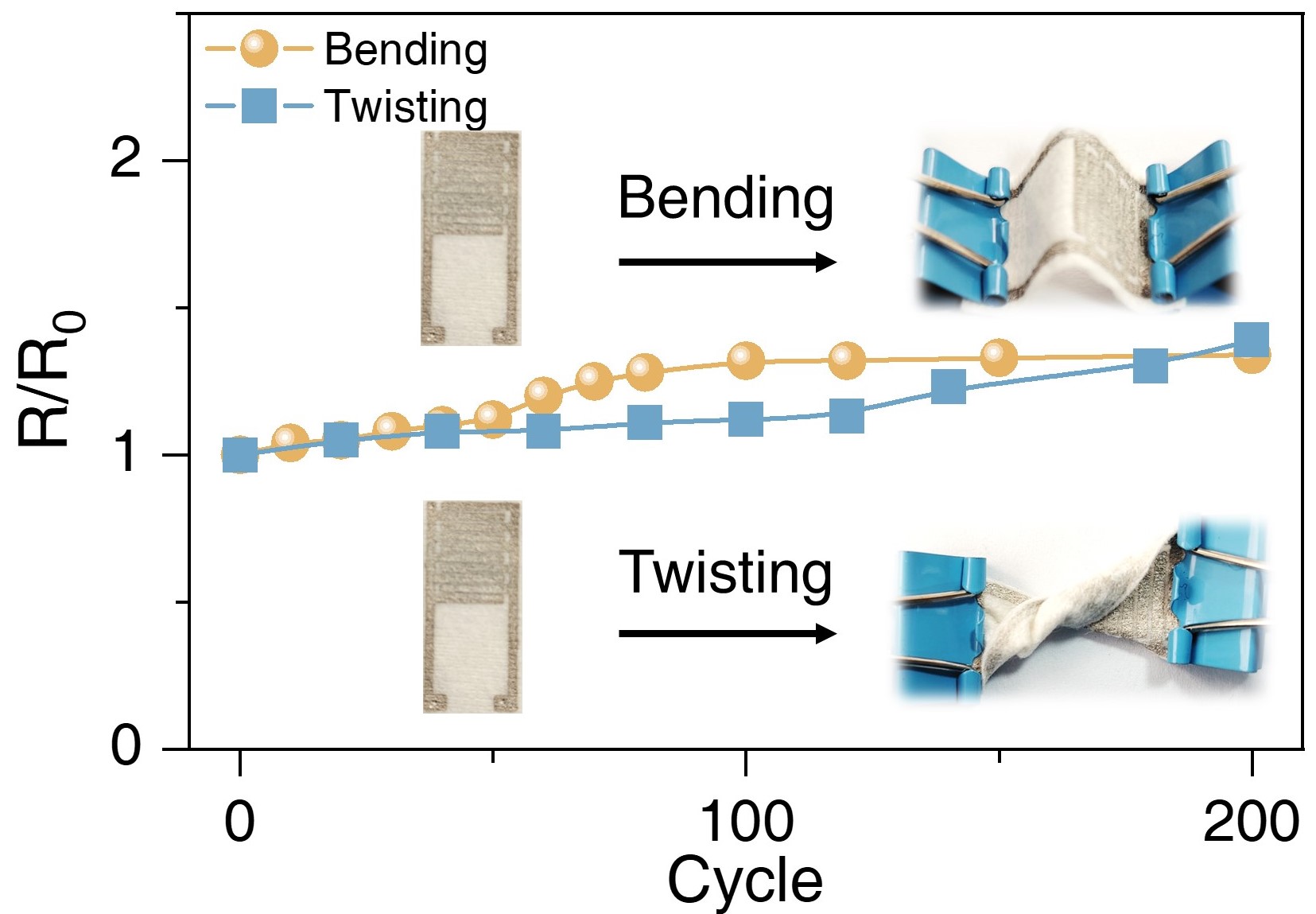


**Figure S4.** Electrical conductivity test of textile-based interdigitated electrode.


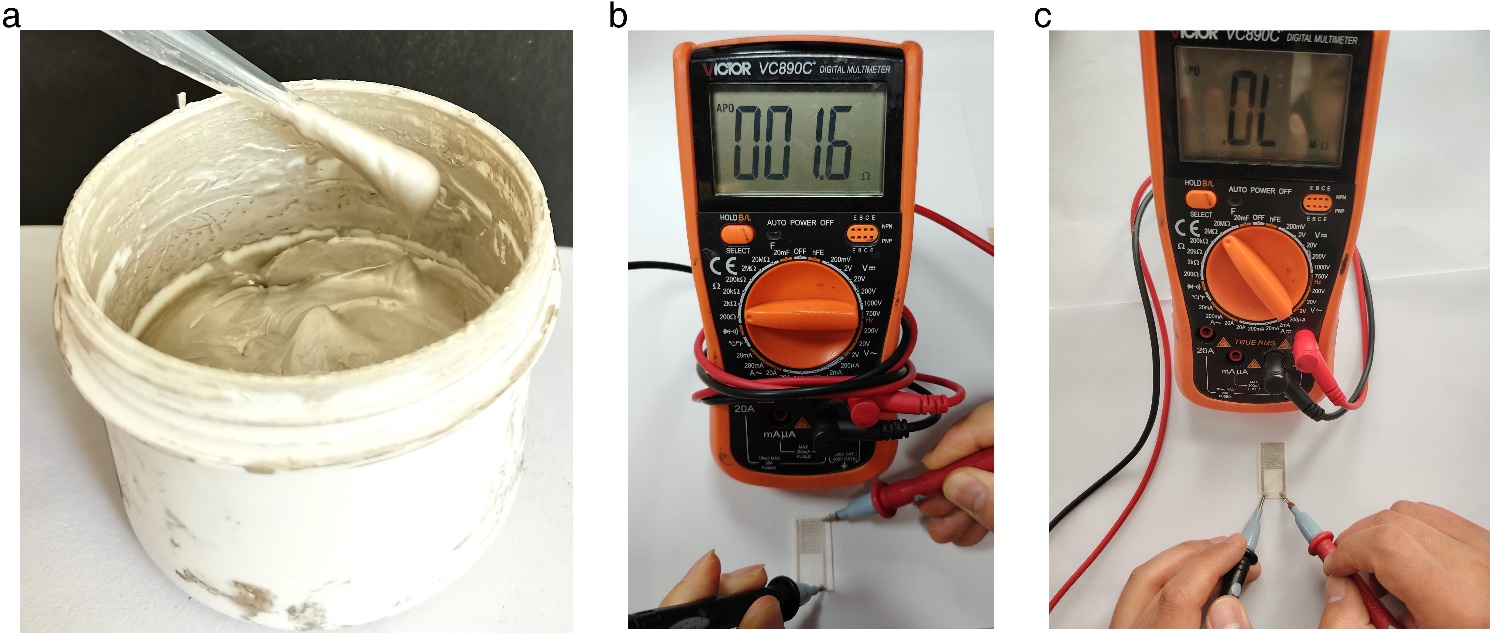


**Figure S5.** Photograph of silver paste and the conductivity test of screen-printed interdigitated electrode.


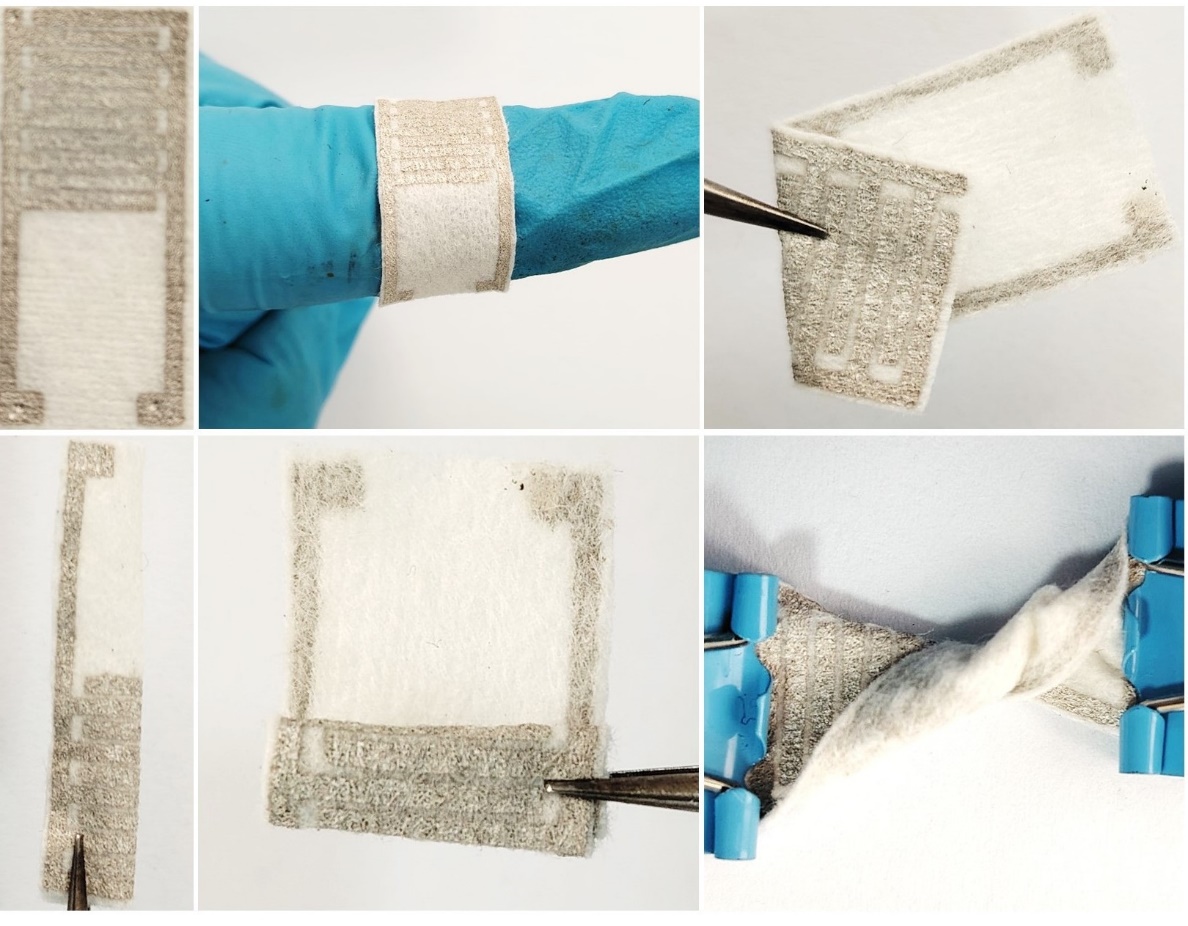


**Figure S6.** Flexibility demonstrations of screen-printed interdigitated electrode.


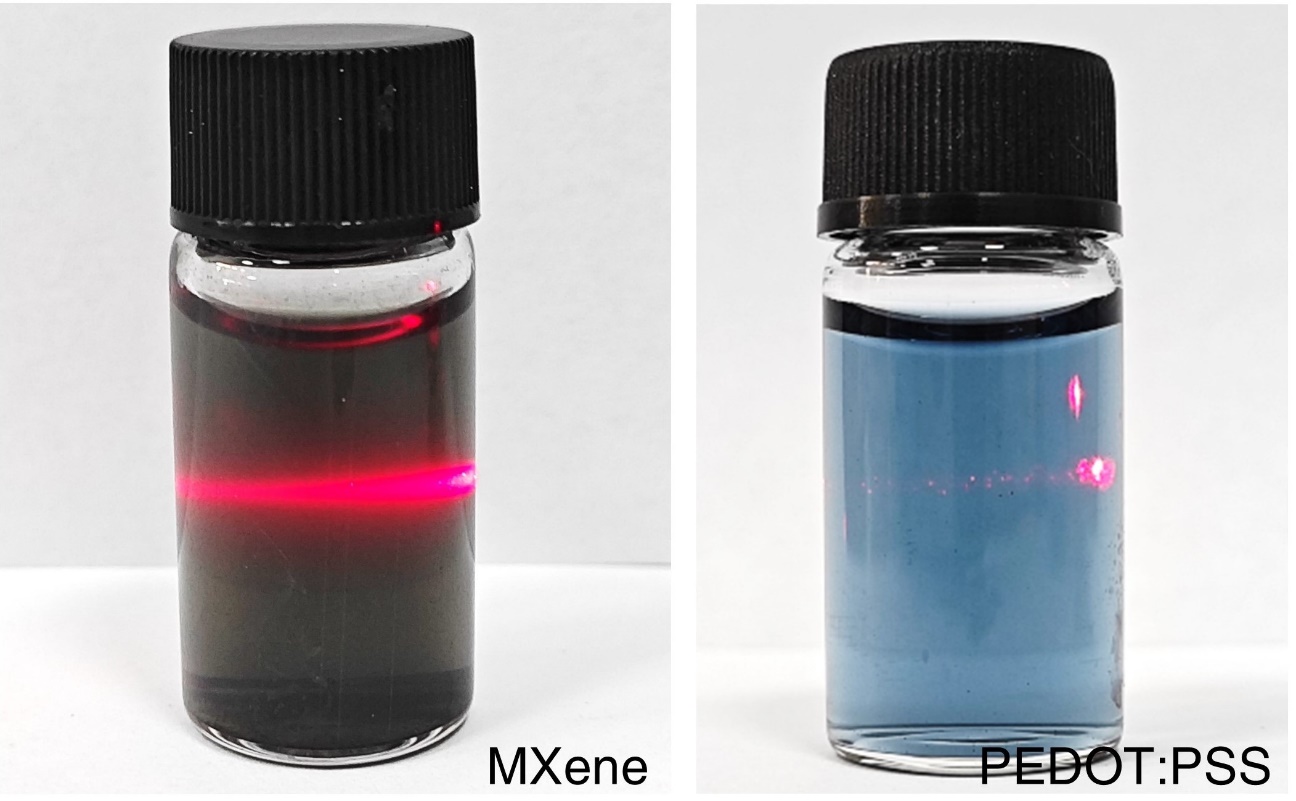


**Figure S7.** Tyndall effect of MXene and PEDOT:PSS dispersion.


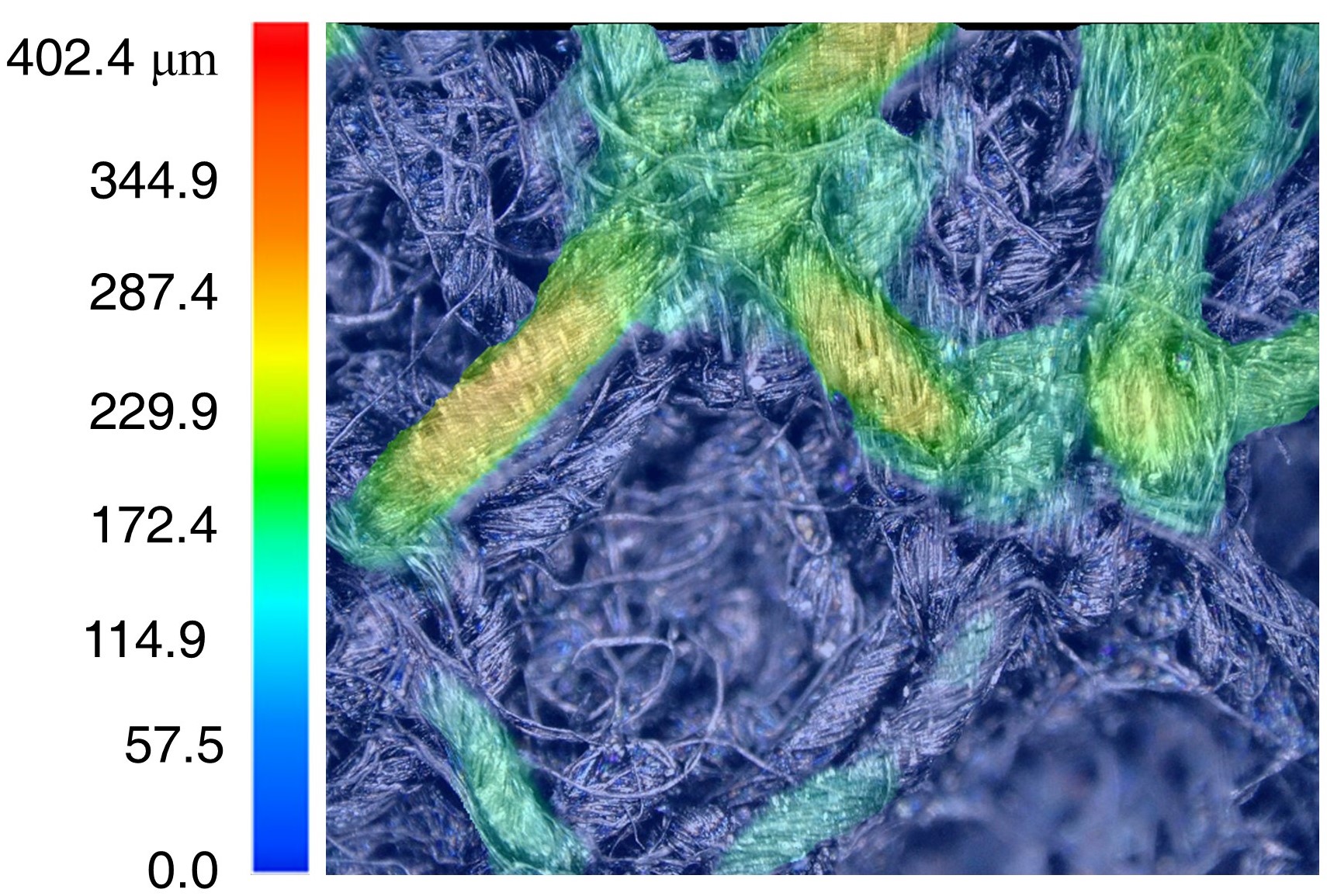


**Figure S8.** 3D ultra-depth microscope image of the 3D textile sensing electrode.


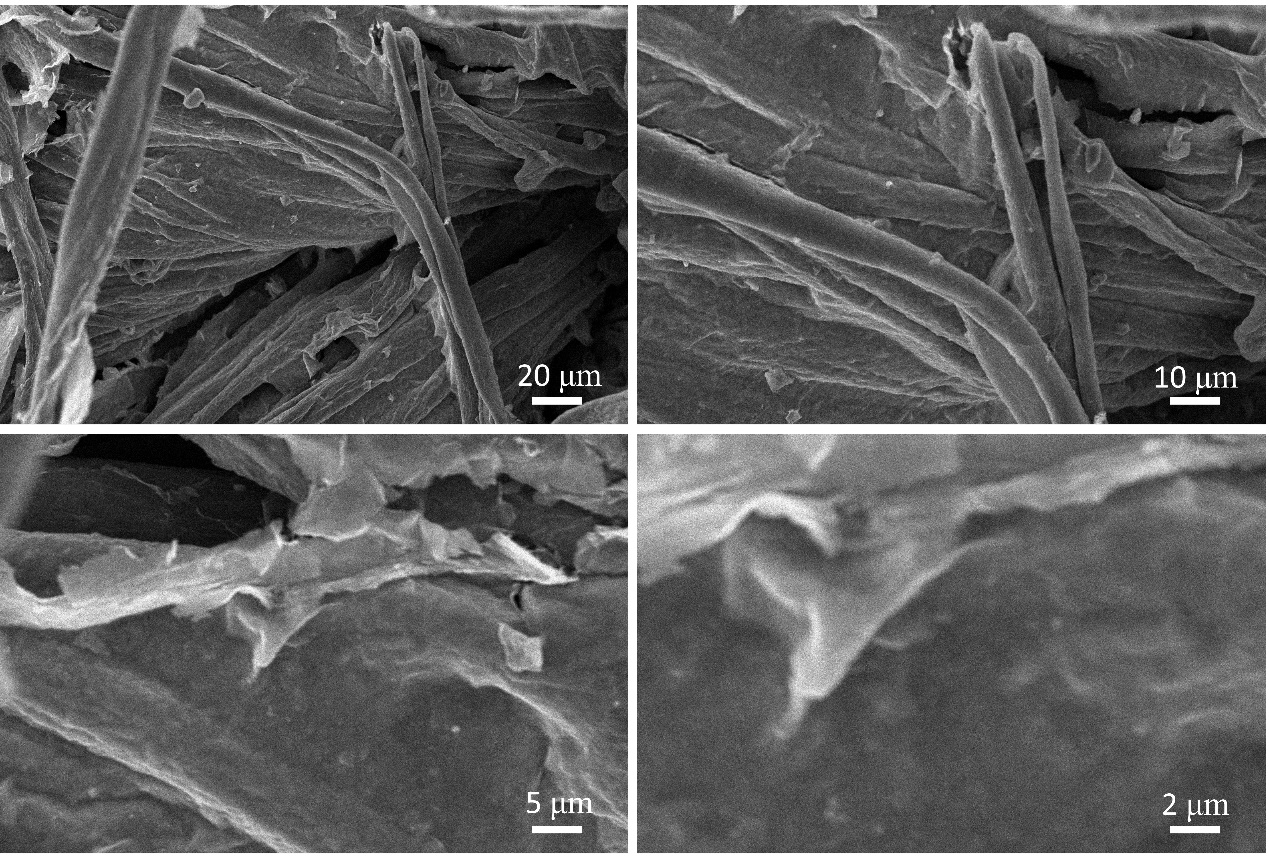


**Figure S9.** SEM images of M1 sensing electrode.


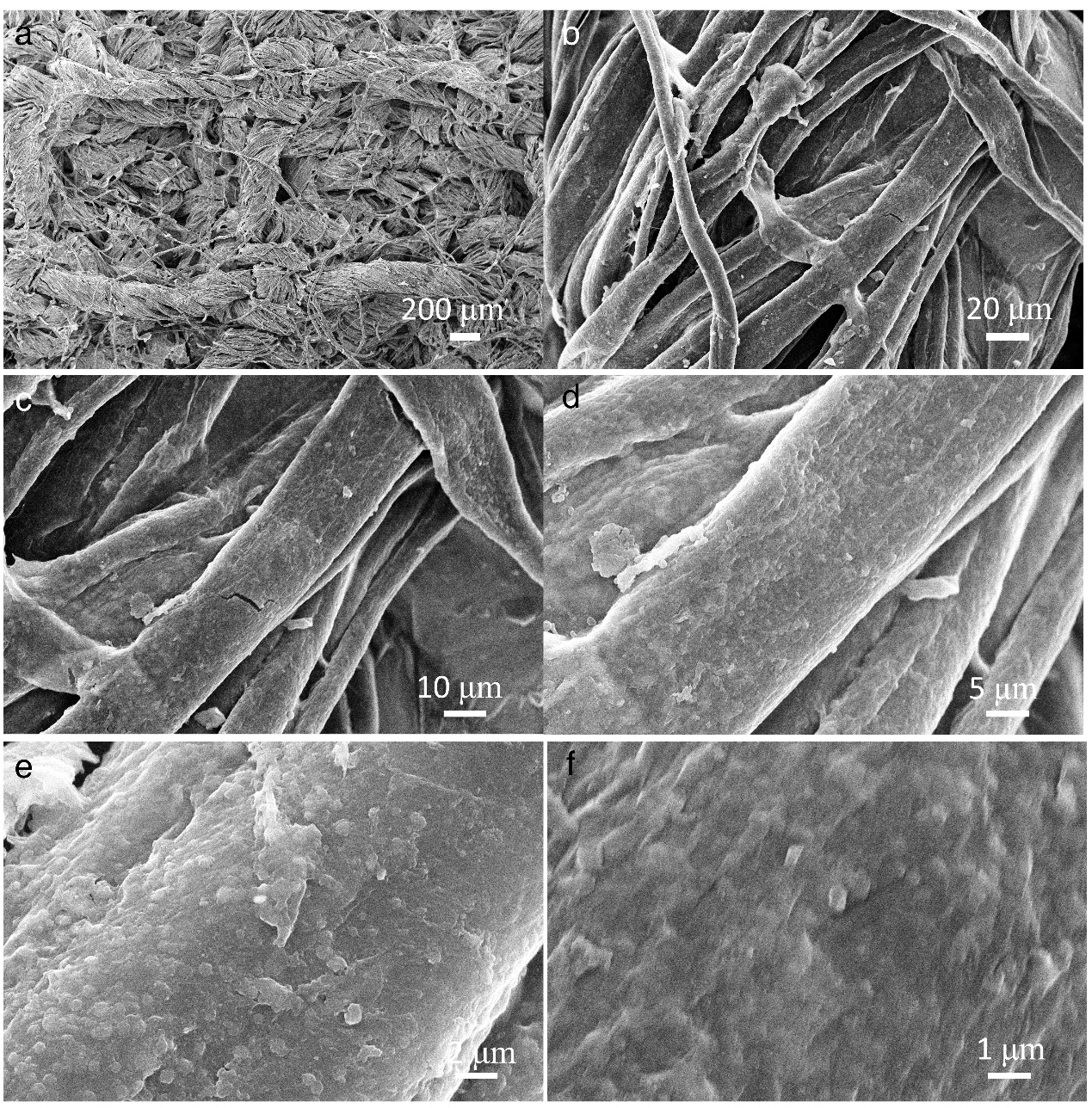


**Figure S10.** SEM images of M1P1 sensing electrode.


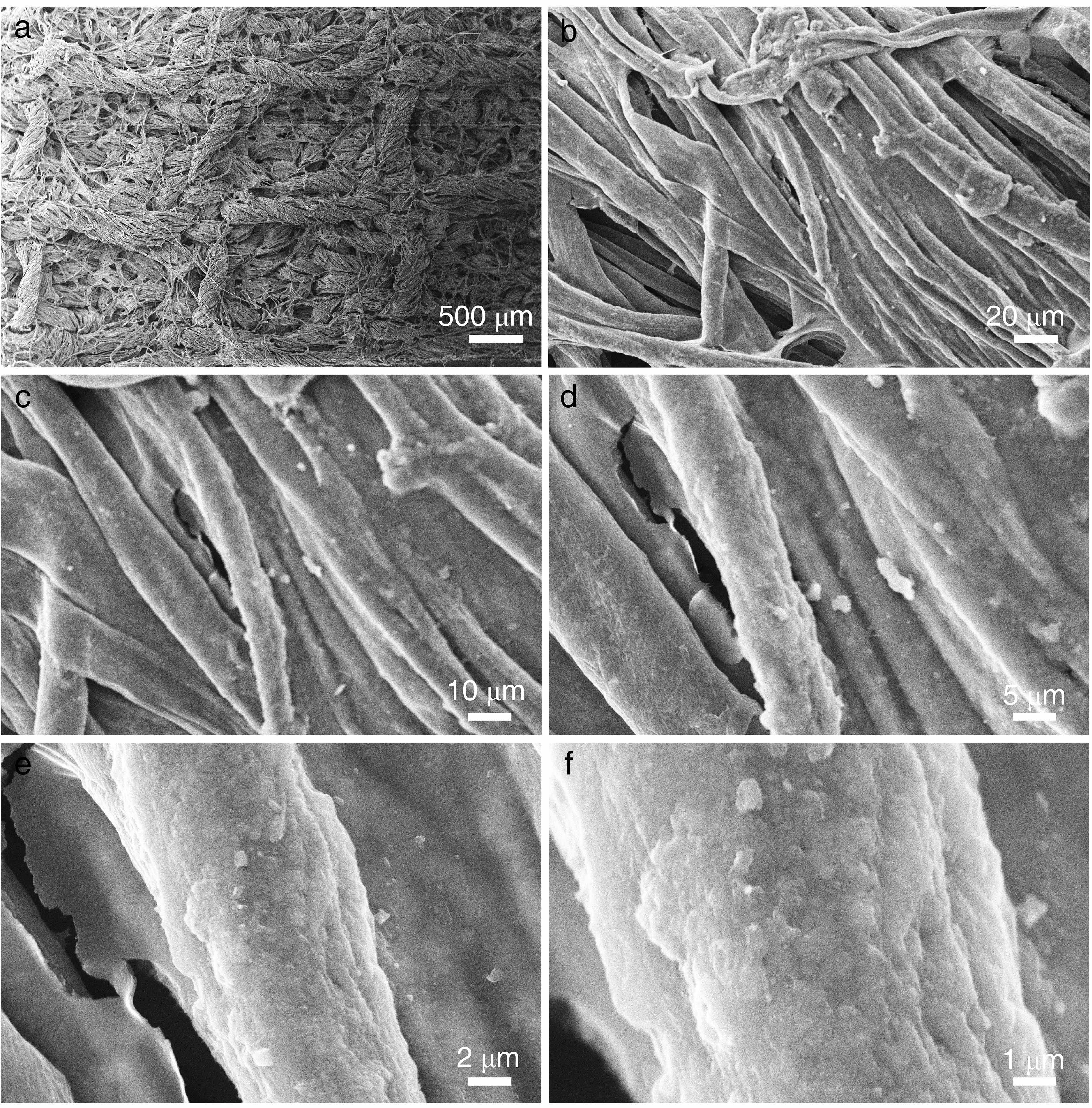


**Figure S11.** SEM images of M2P2 sensing electrode.


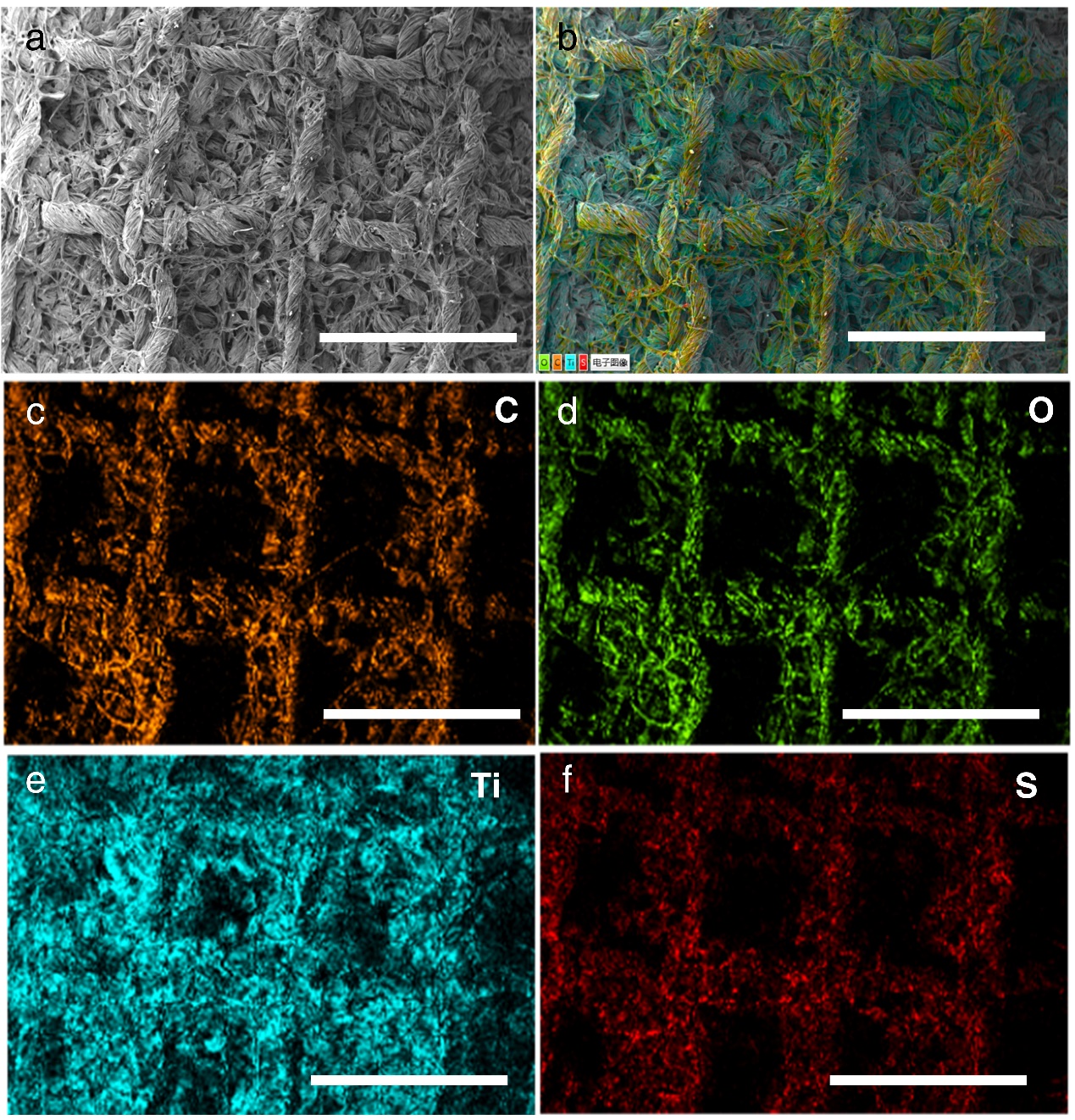


**Figure S12.** EDS mapping images of M2P2 sensing electrode. (a) SEM image of M2P2 sensing electrode. (b) EDS element hierarchical image. EDS elemental mapping of (c) C, (d) O, (e) Ti, (f) S. Scale bar: 500 μm.


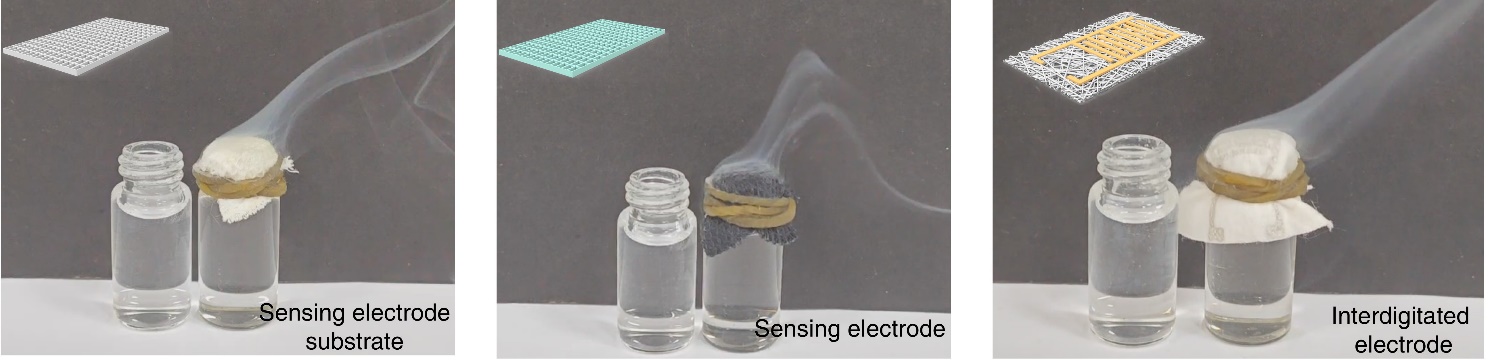


**Figure S13.** Air permeability demonstrations. Two glass bottles containing NH_3_·H_2_O and HCl covered with sensing electrode substrate, interdigitated electrode, and sensing electrode


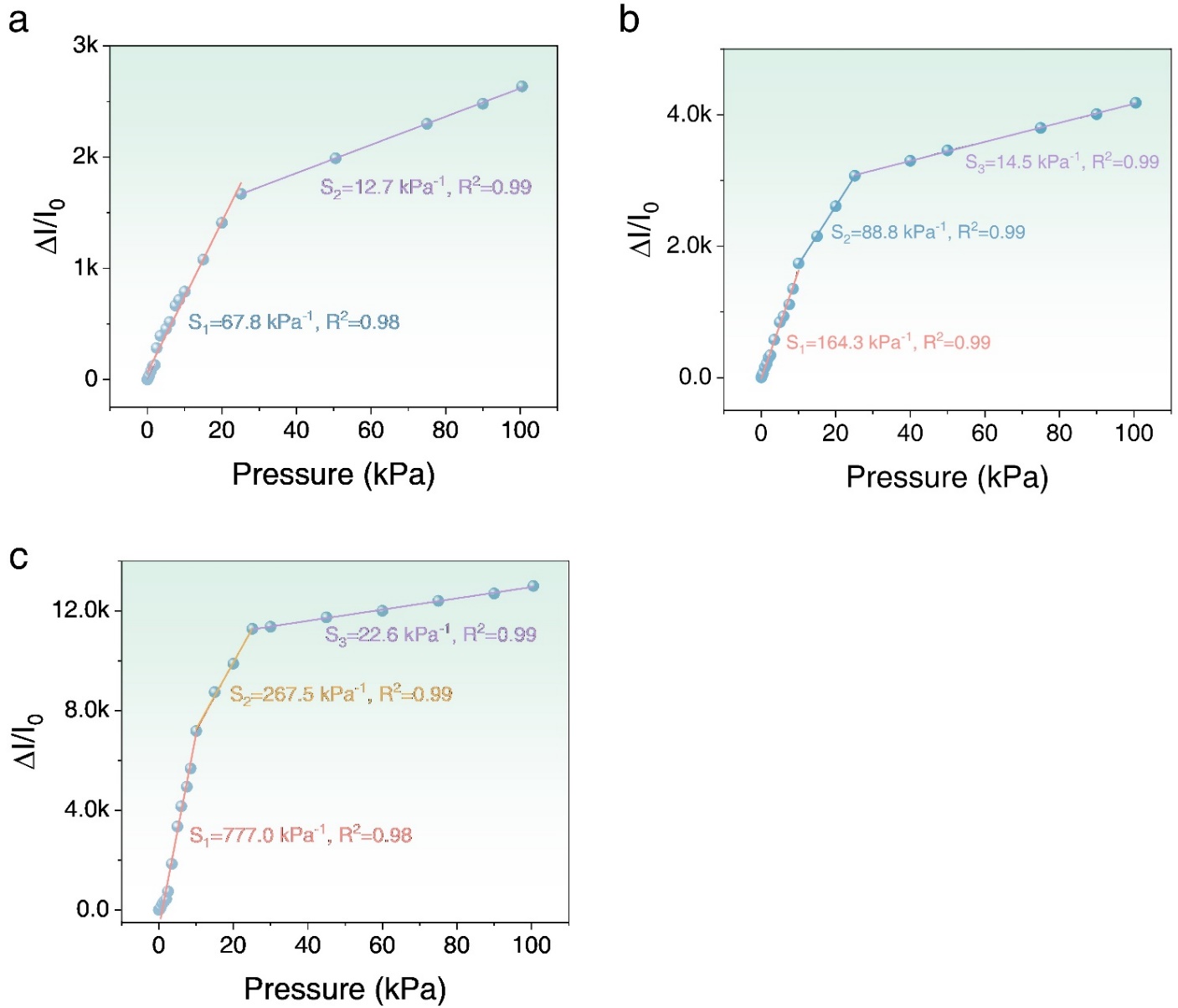


**Figure S14.** Sensitivities of (a) M1-, (b) M1P1-, (c) M2P1-based tactile sensors.


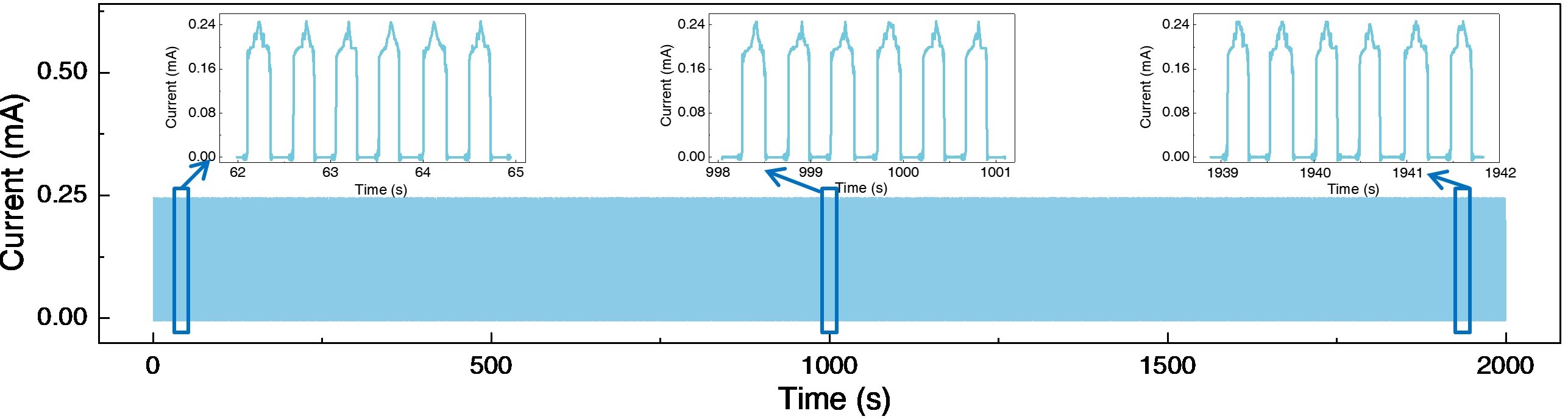


**Figure S15.** Cycling stability of TBTS over 4000 loading/unloading cycles.


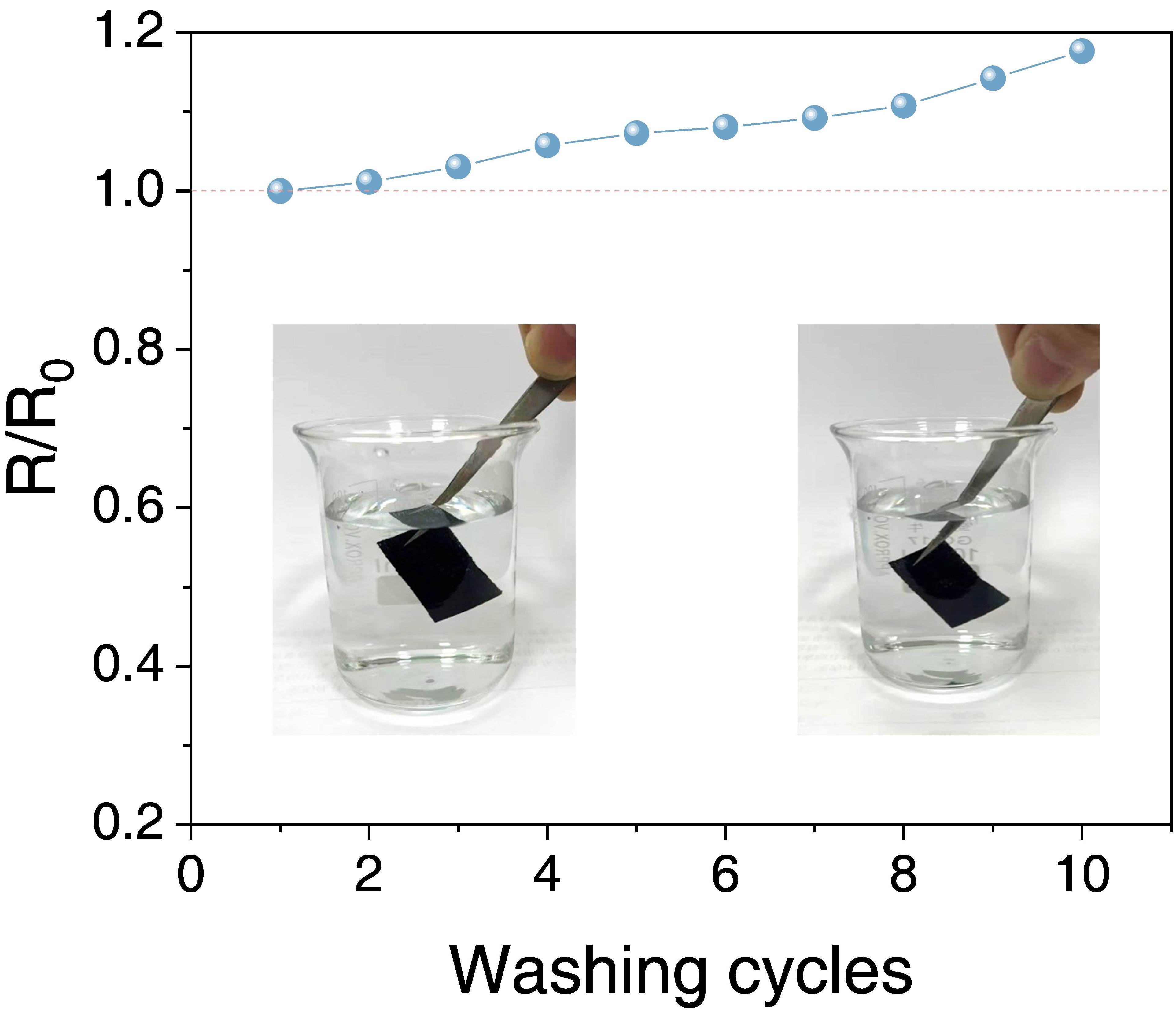


**Figure S16.** Resistance viraition of sensing electrode after 10 washing cycles


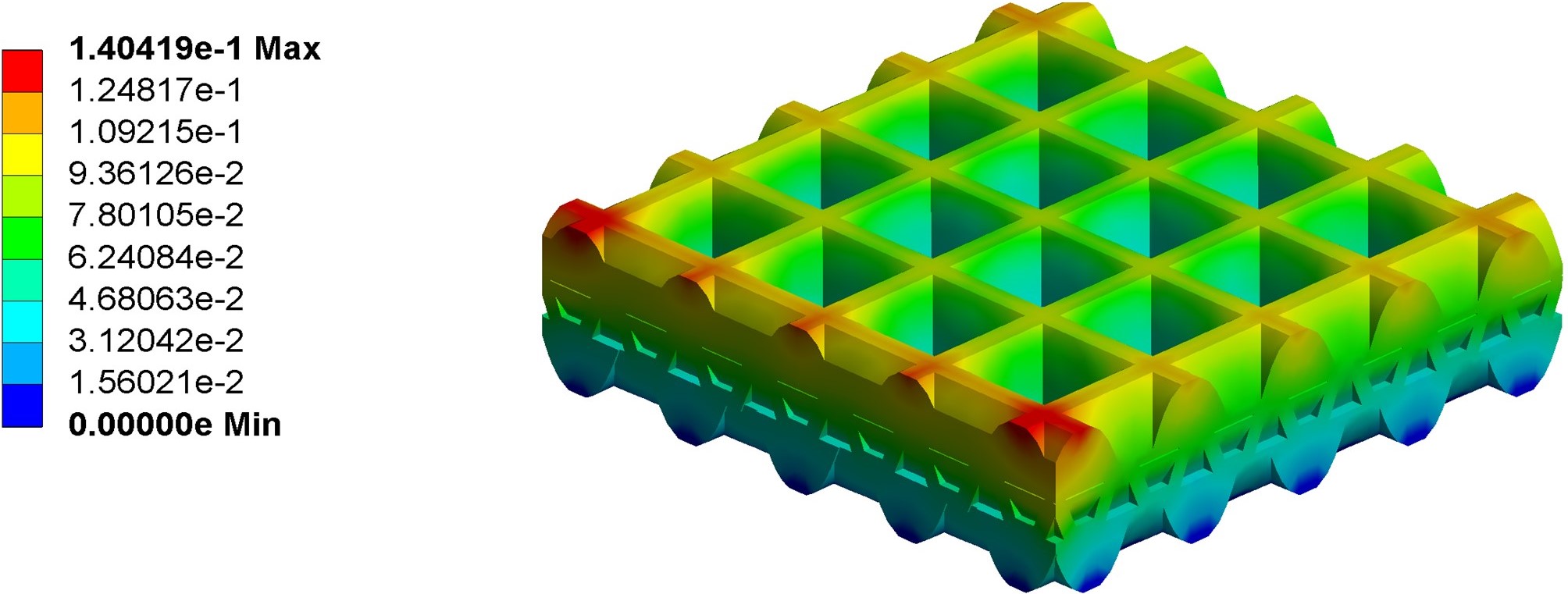


**Figure S17.** Finite element modeling of the strain distribution of the sensing layer under 10 kPa.


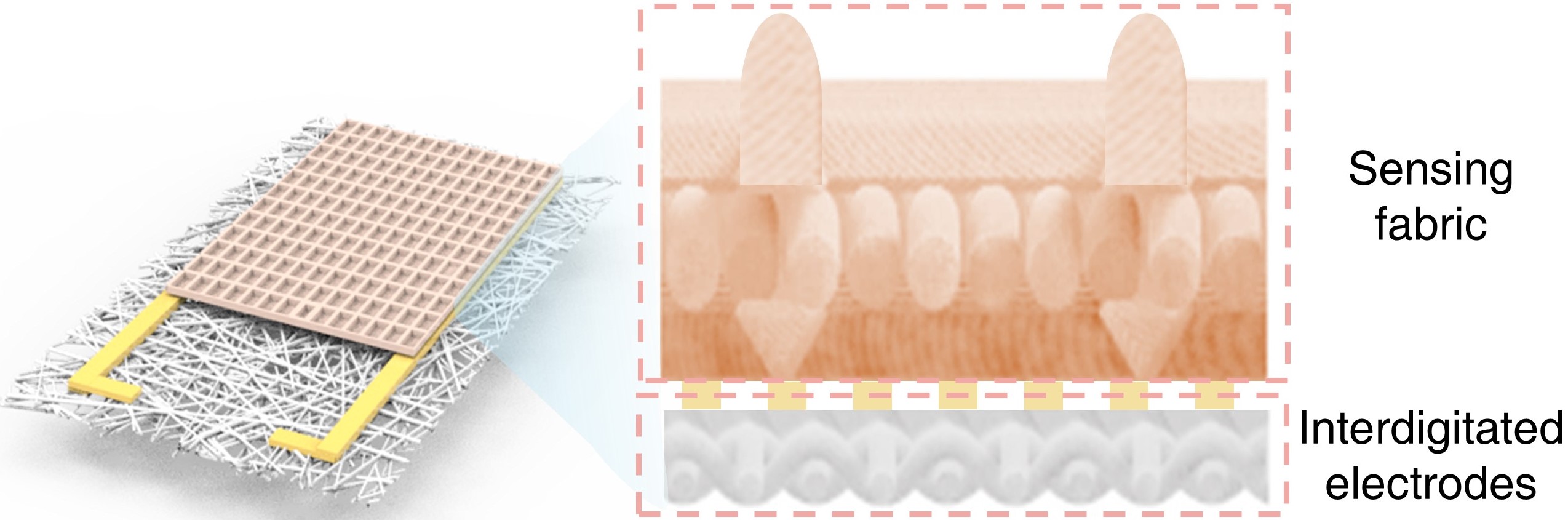


**Figure S18.** Structure diagram of TBTS.


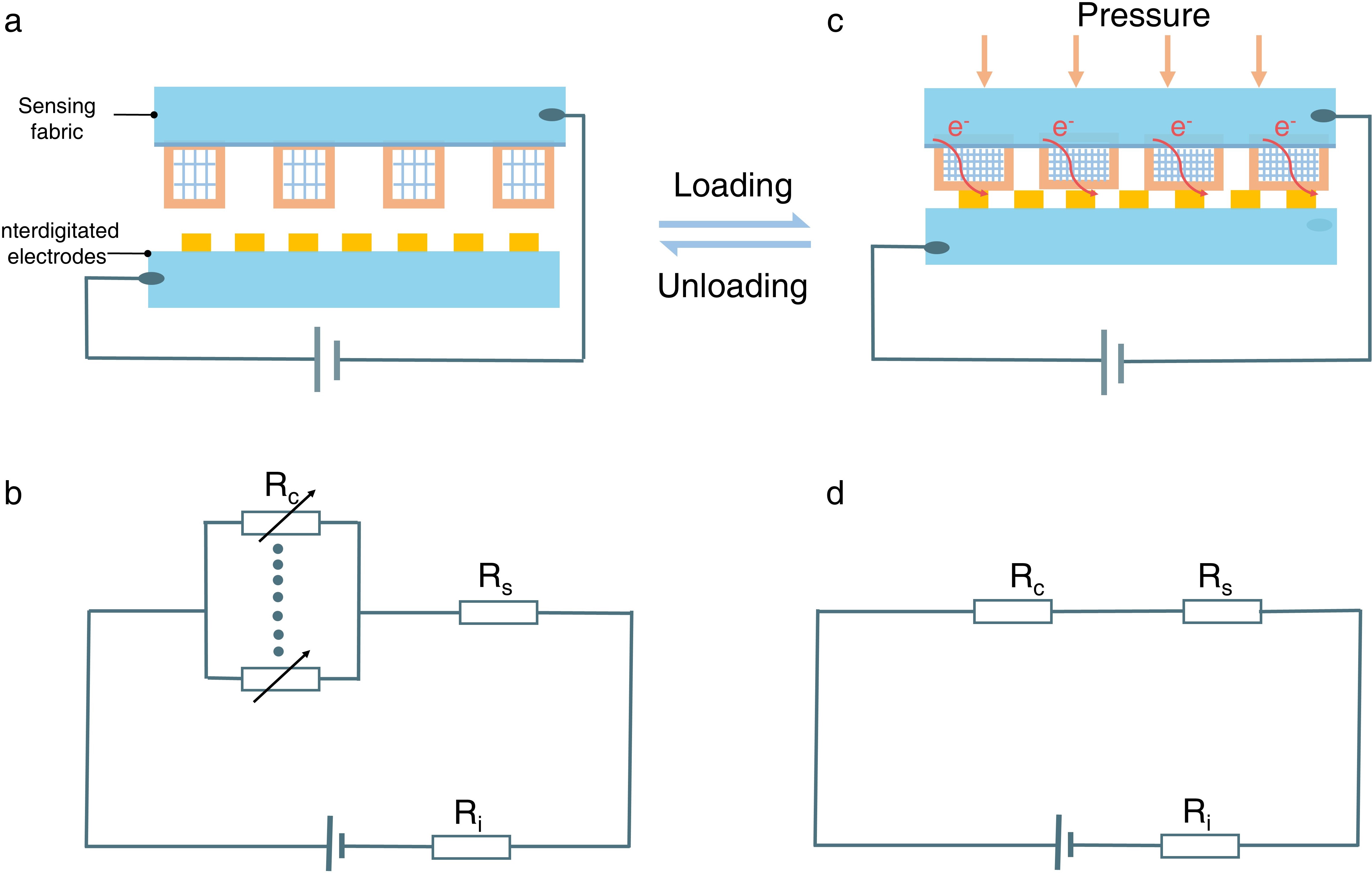


**Figure S19.** The sensing mechanism of TBTS-based sensor. (a) and (b) Structure diagram of the sensor without applied pressure and the corresponding circuit diagram. (c) and (d) Structure diagram of the sensor with applied pressure and the corresponding circuit diagram. R_c_, R_i_, and R_s_ represent contact resistance between interdigitated electrode and sensing electrode, resistance of interdigitated electrodes, and resistance of sensing electrode, respectively.


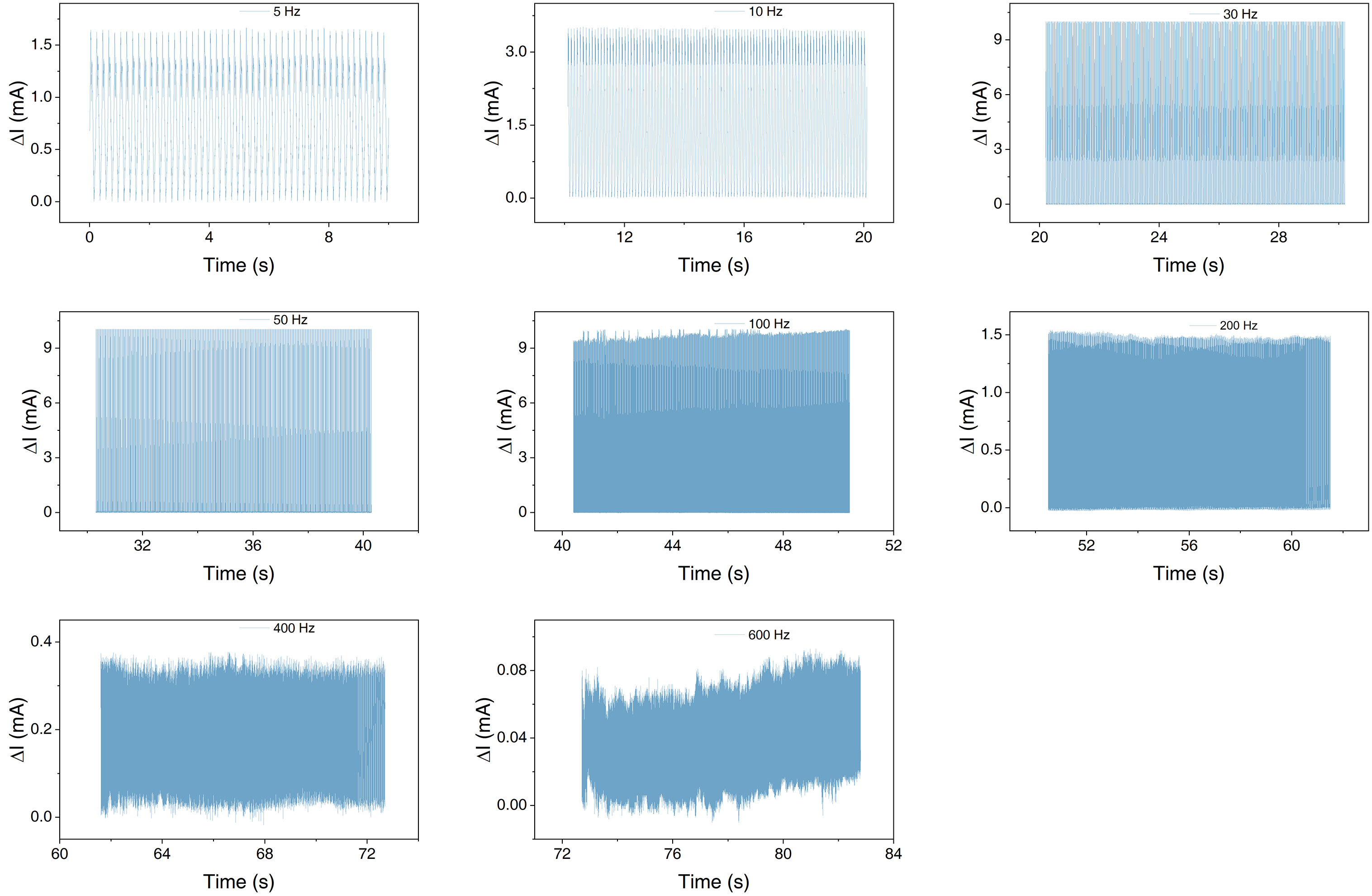


**Figure S20.** Response curves of the sensor towards the dynamic force with frequency from 5 Hz to 600 Hz.


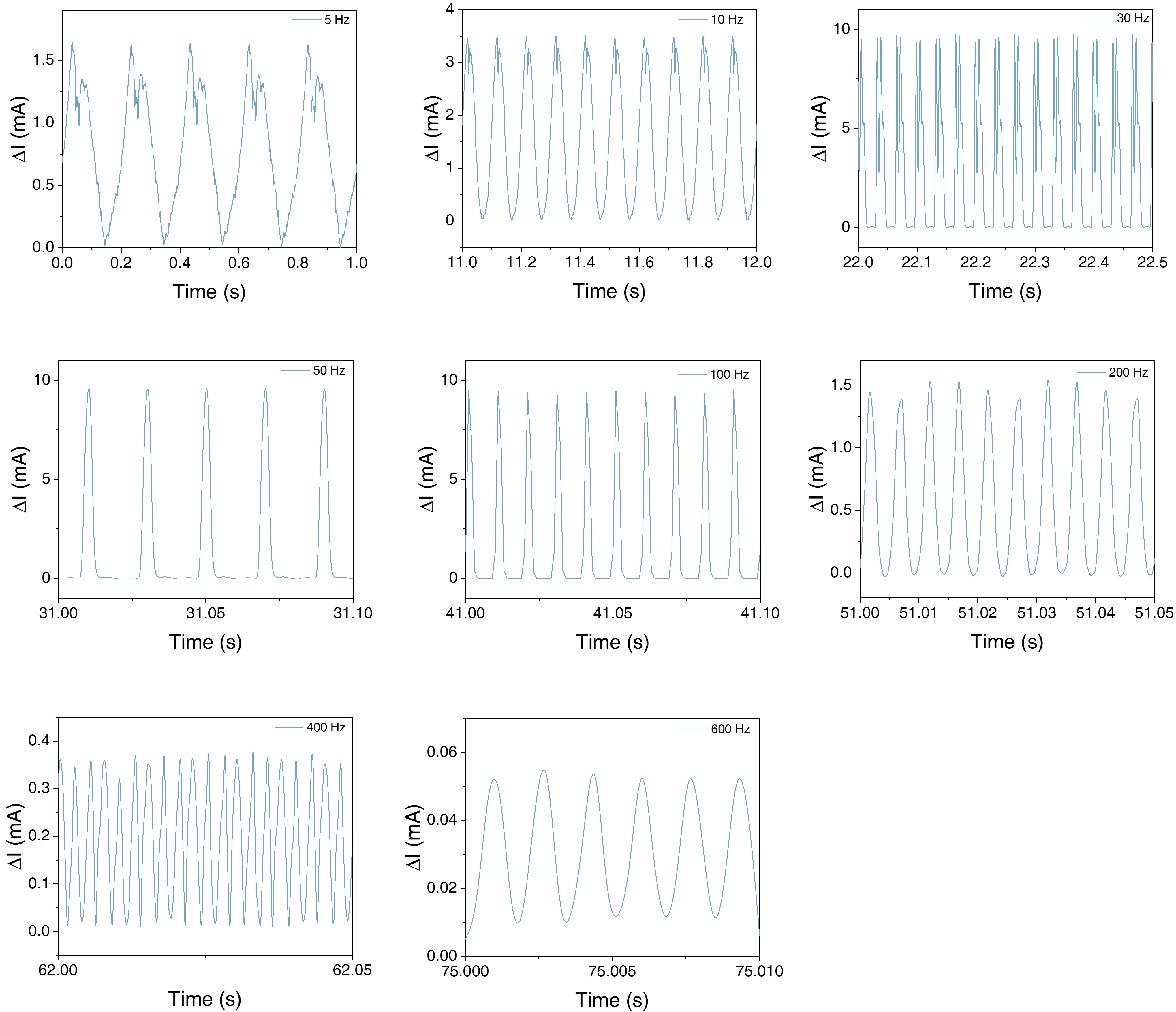


**Figure S21.** Magnified response curves of the sensor towards the dynamic force with frequency from 5 Hz to 600 Hz.


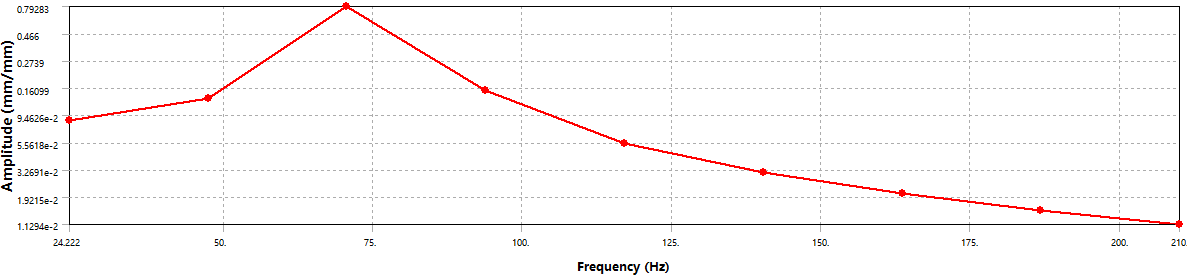


**Figure S22.** Amplitude as a function of vibration frequency under a dynamic force of 2 kPa.


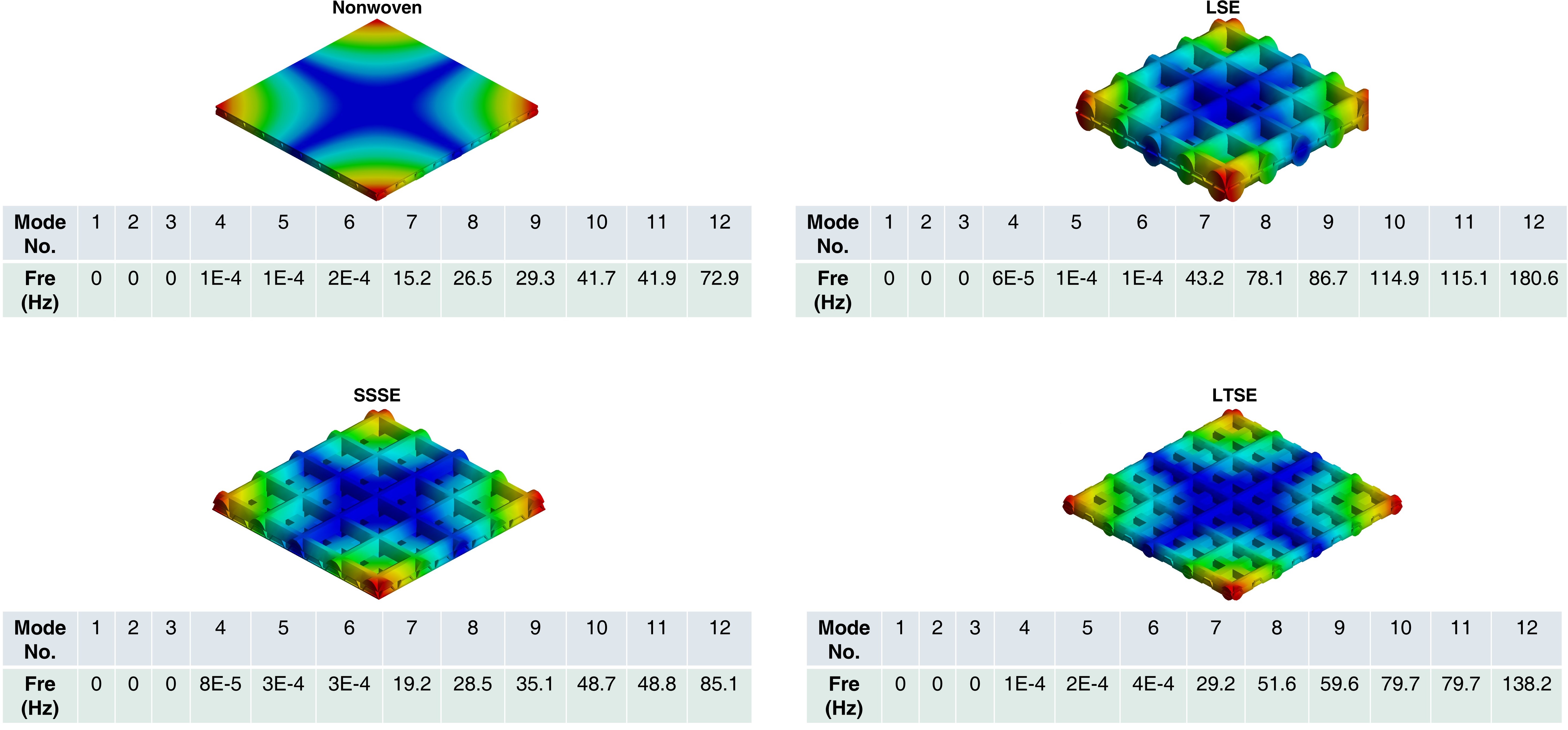


**Figure S23.** 1^st^ natural frequency of various sensing electrode based on FEA method.


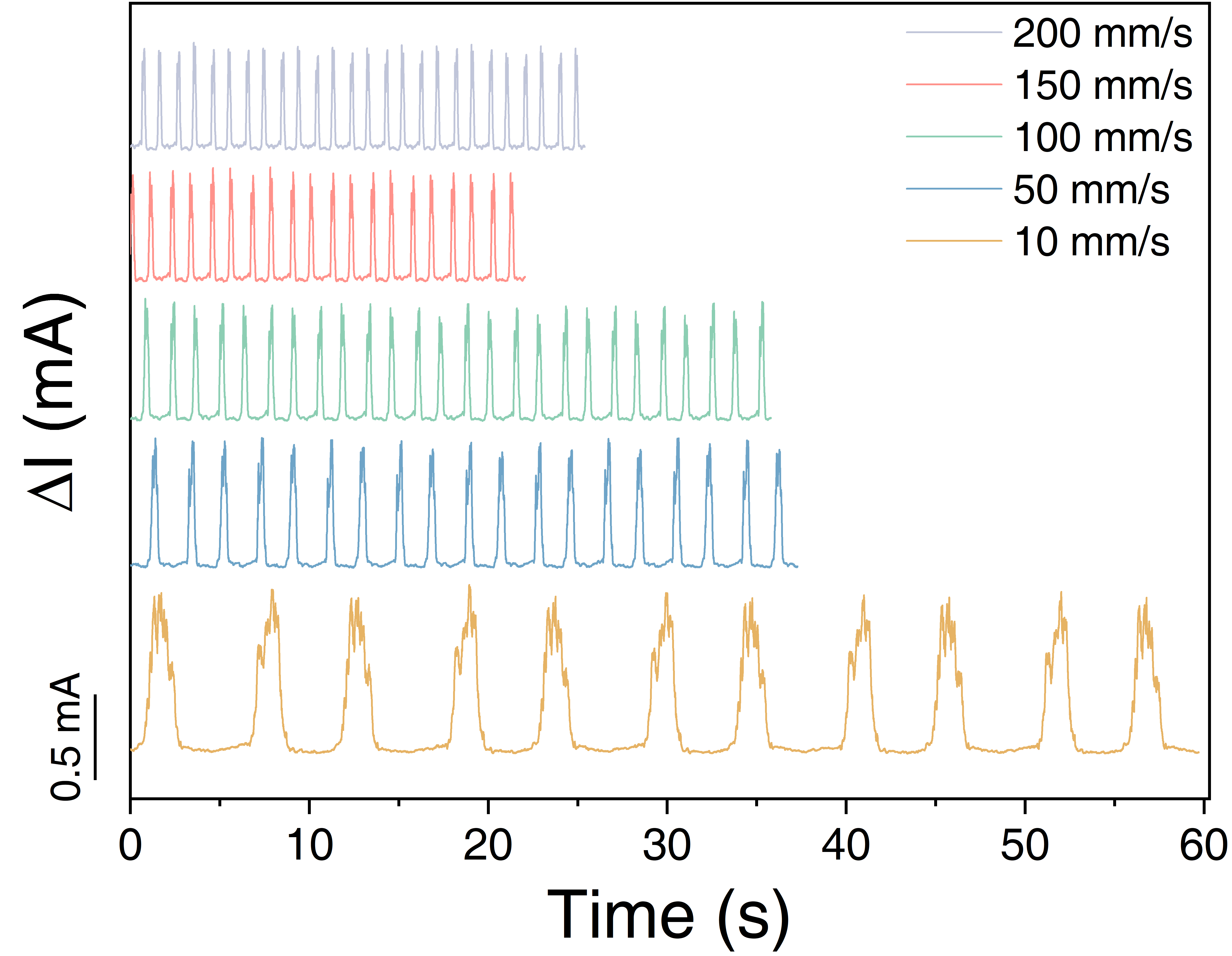


**Figure S24.** Current response signals of the TBTS sliding on the braille “6” under 10~200 mm s^-1^.


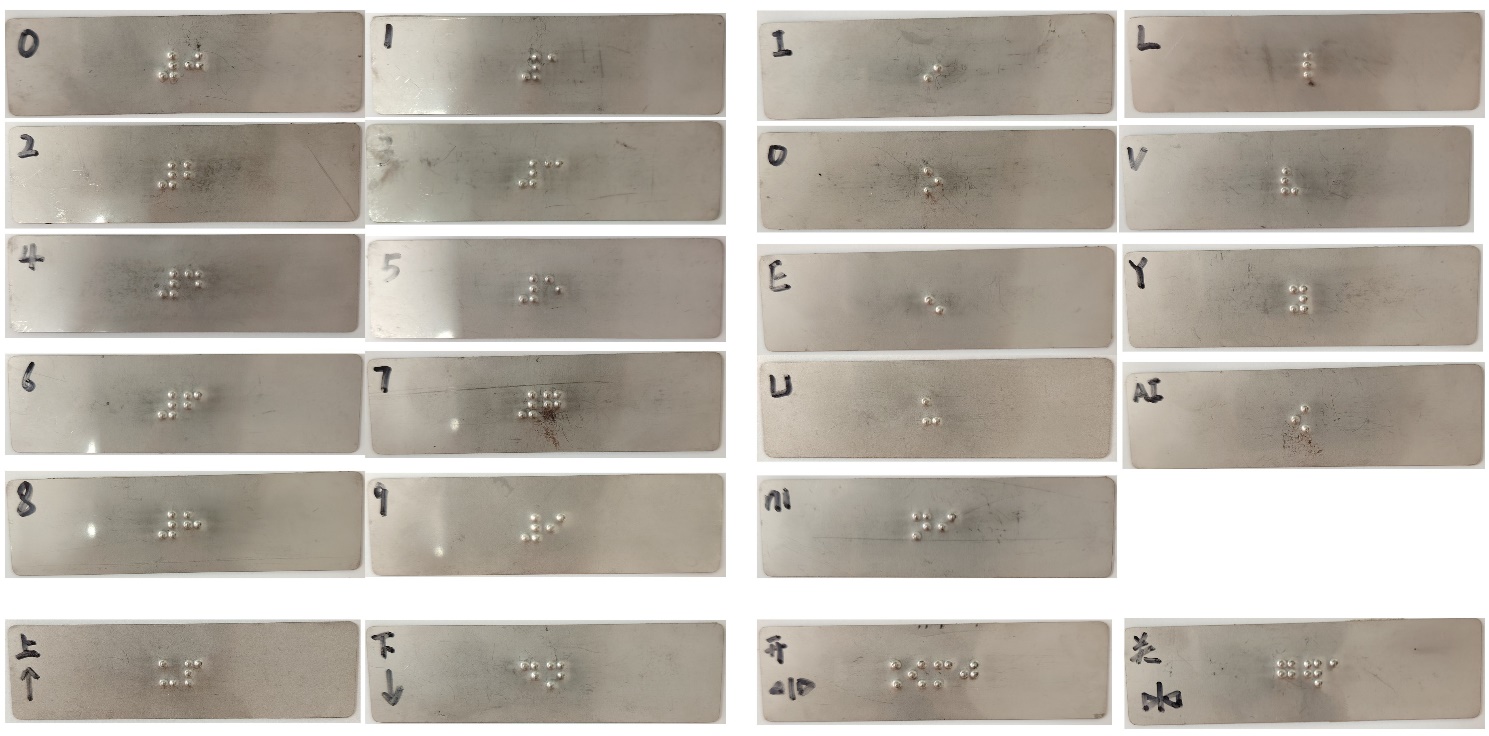


**Figure S25.** Photograph of 23 customized braille used for recognition.


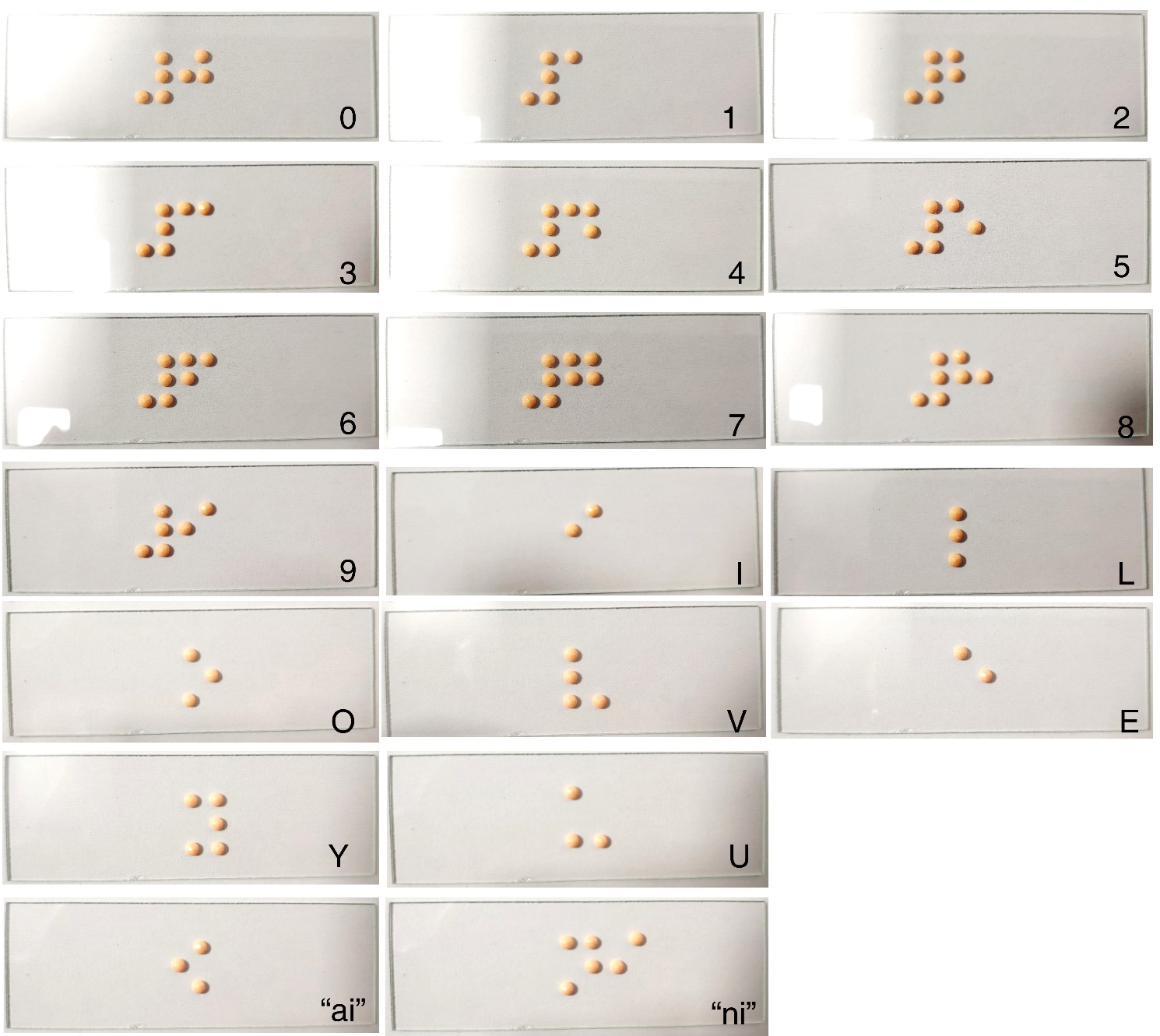


**Figure S26.** Photograph of 19 homemade braille used for recognition.


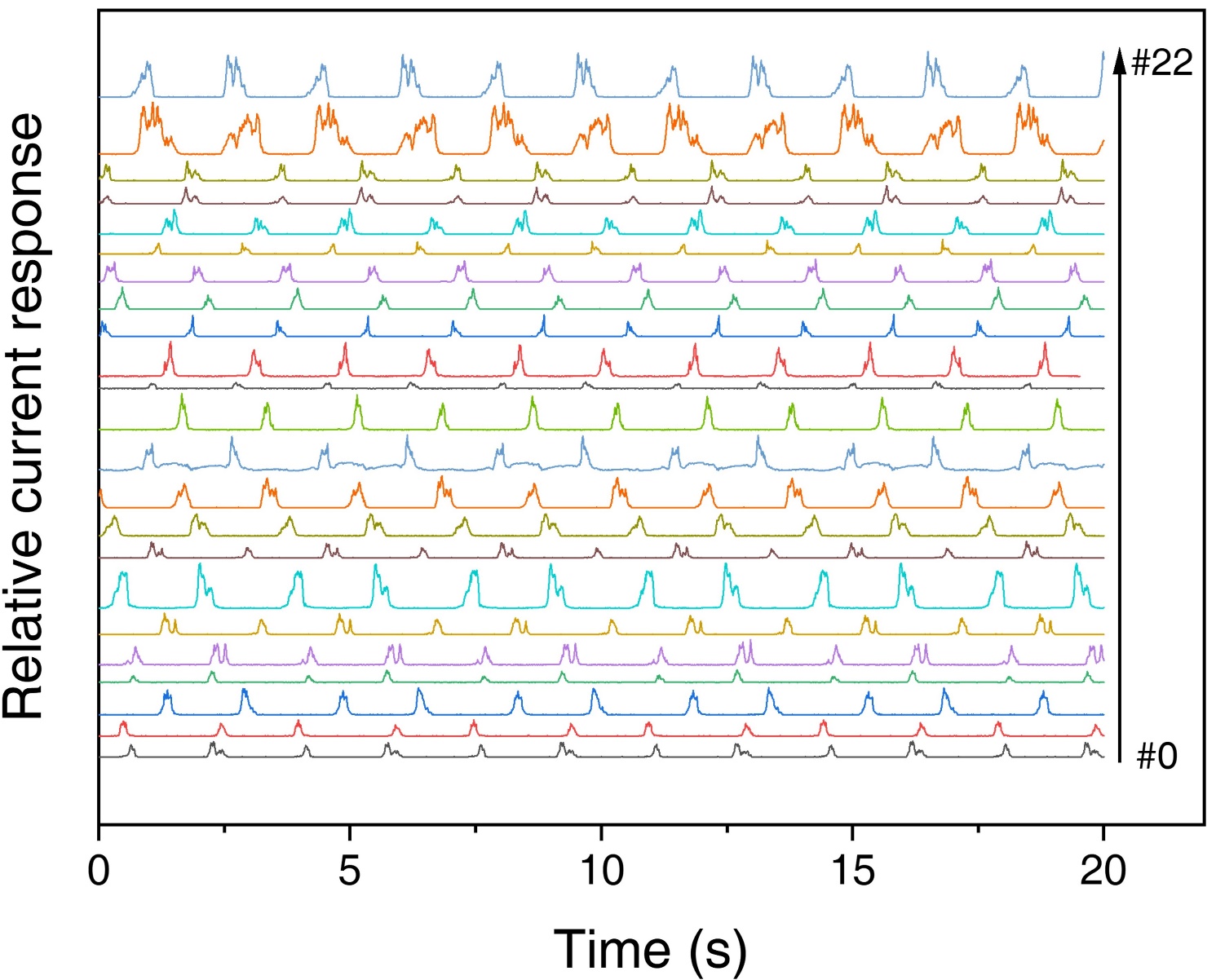


**Figure S27.** Current response of the bionic sensors towards 23 customized braille at a sliding rate of 150 mm s^-1^.


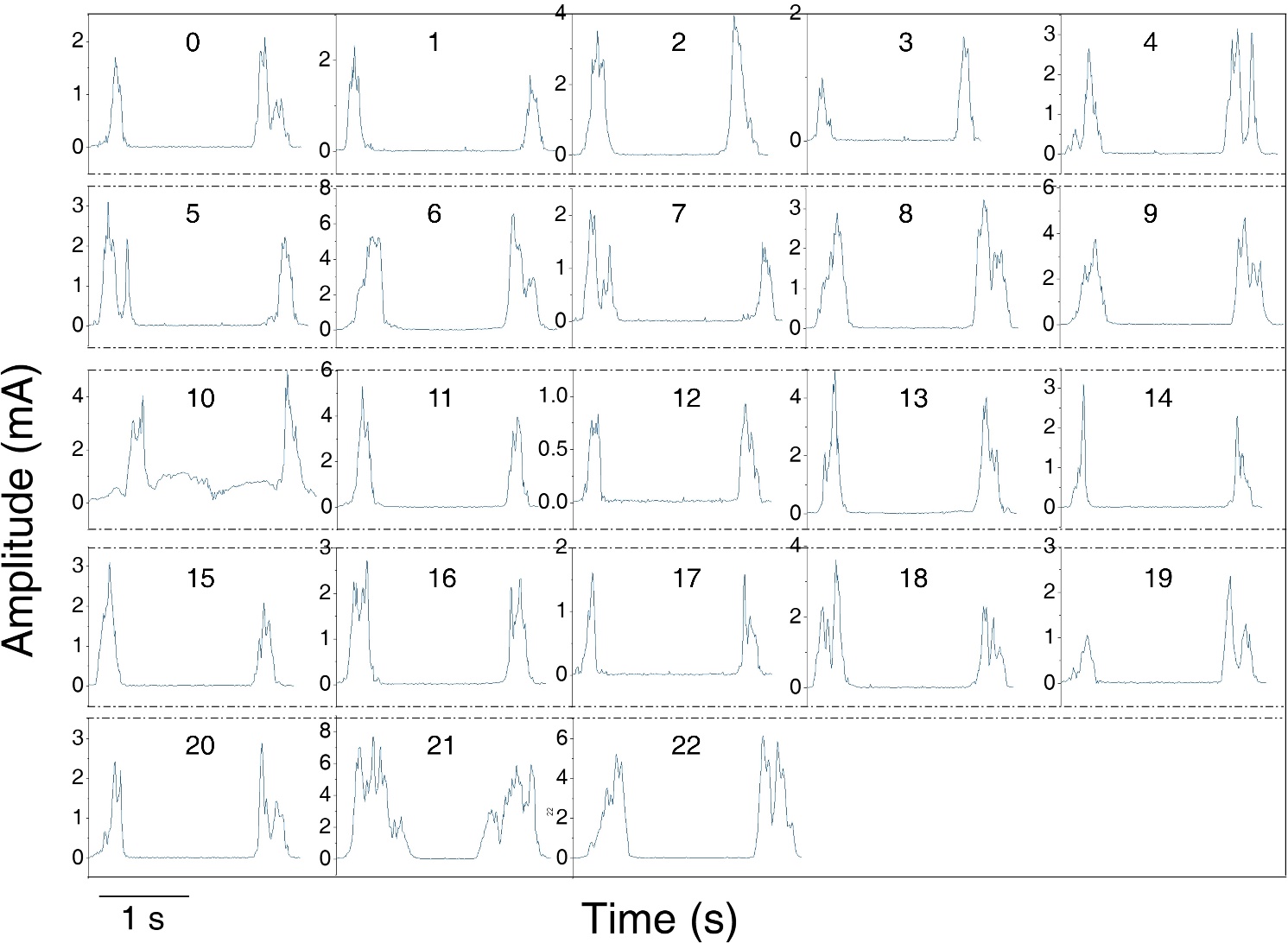


**Figure S28.** Time-domain signals of the bionic sensor corresponding to 23 customized braille at a sliding rage of 150 mm s^-1^.


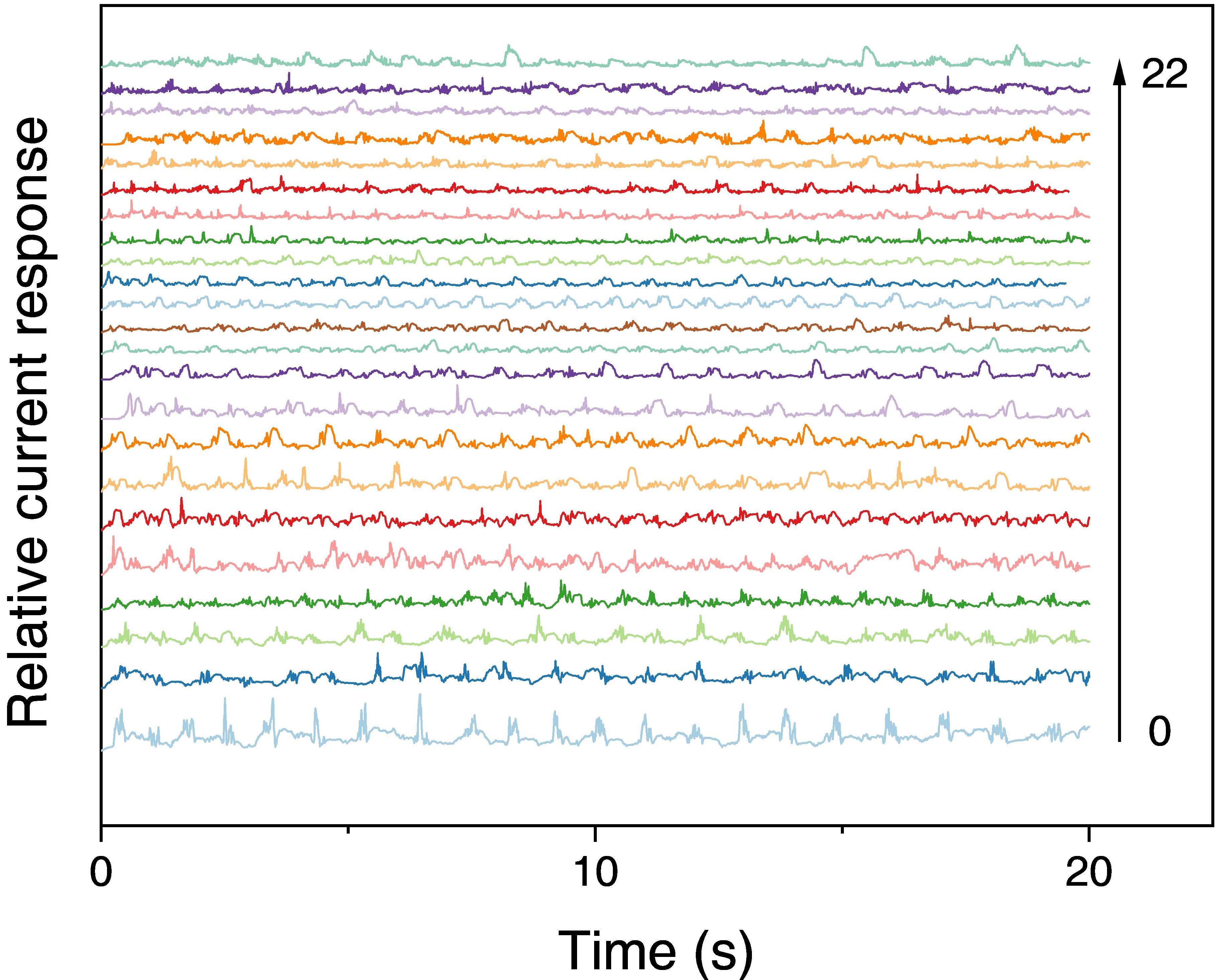


**Figure S29.** Current response of the bionic sensors towards 23 customized braille under random sliding rates.


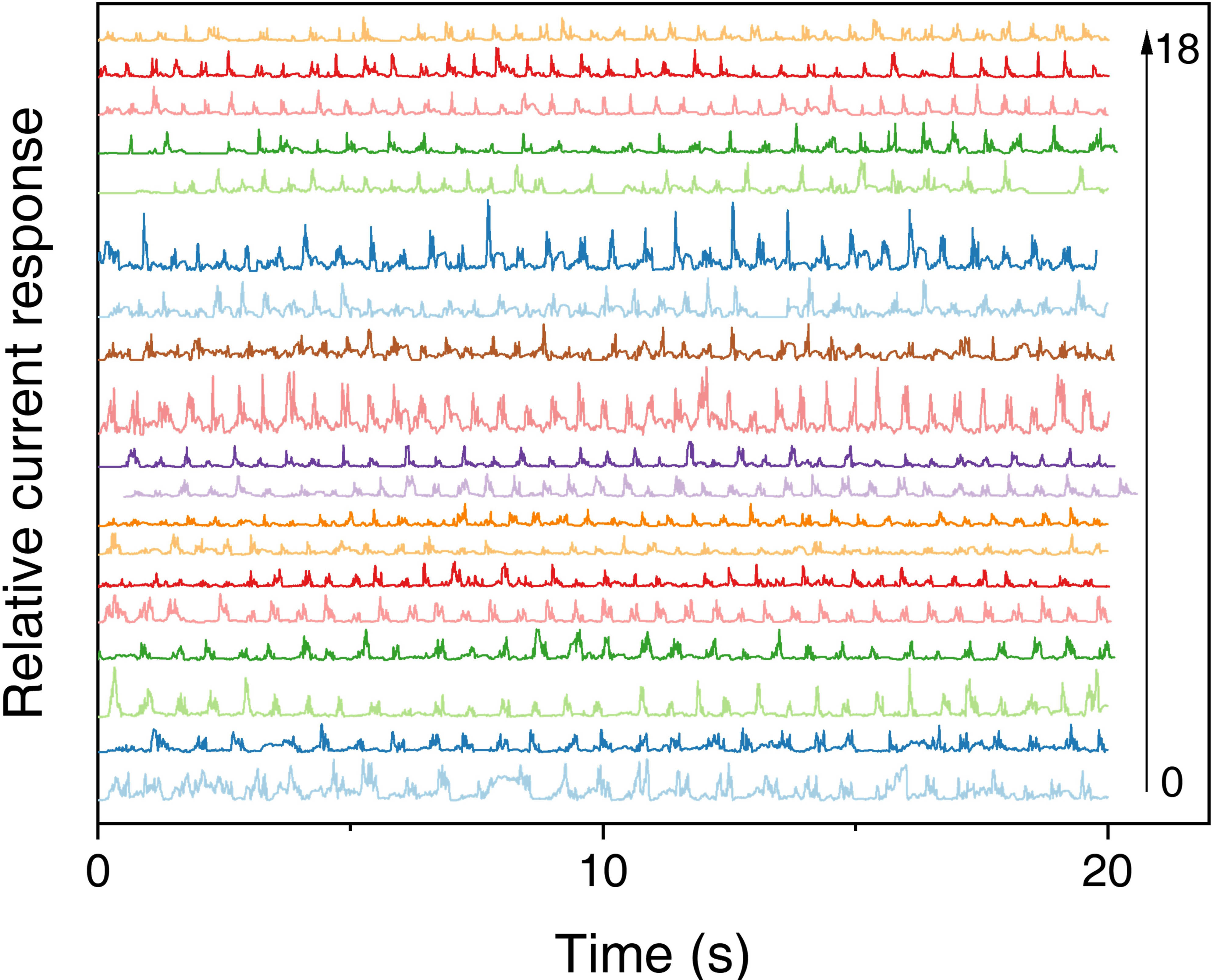


**Figure S30.** Current response of the bionic sensors towards 19 homemade braille under random sliding rates.


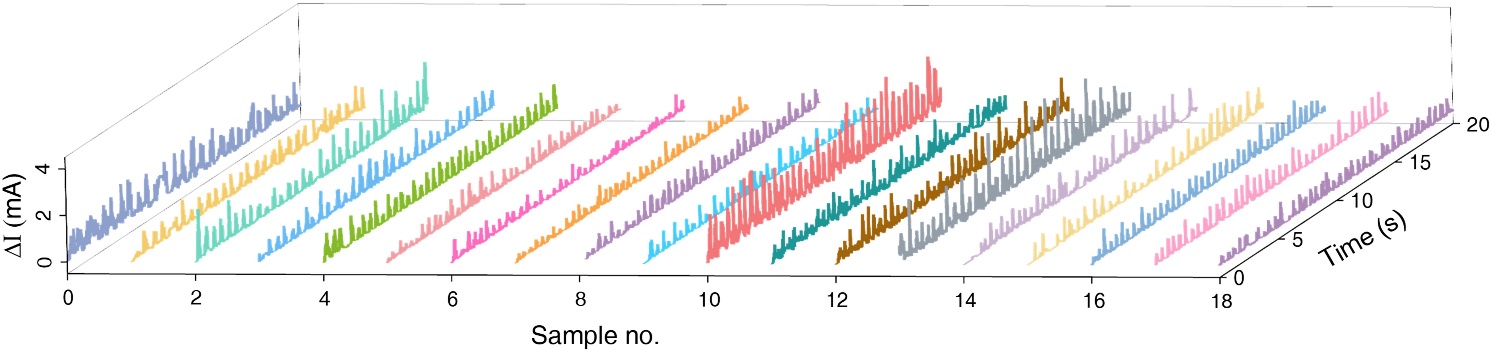


**Figure S31.** Time-domain signals of 19 homemade braille using the sensor at a random sliding rate.


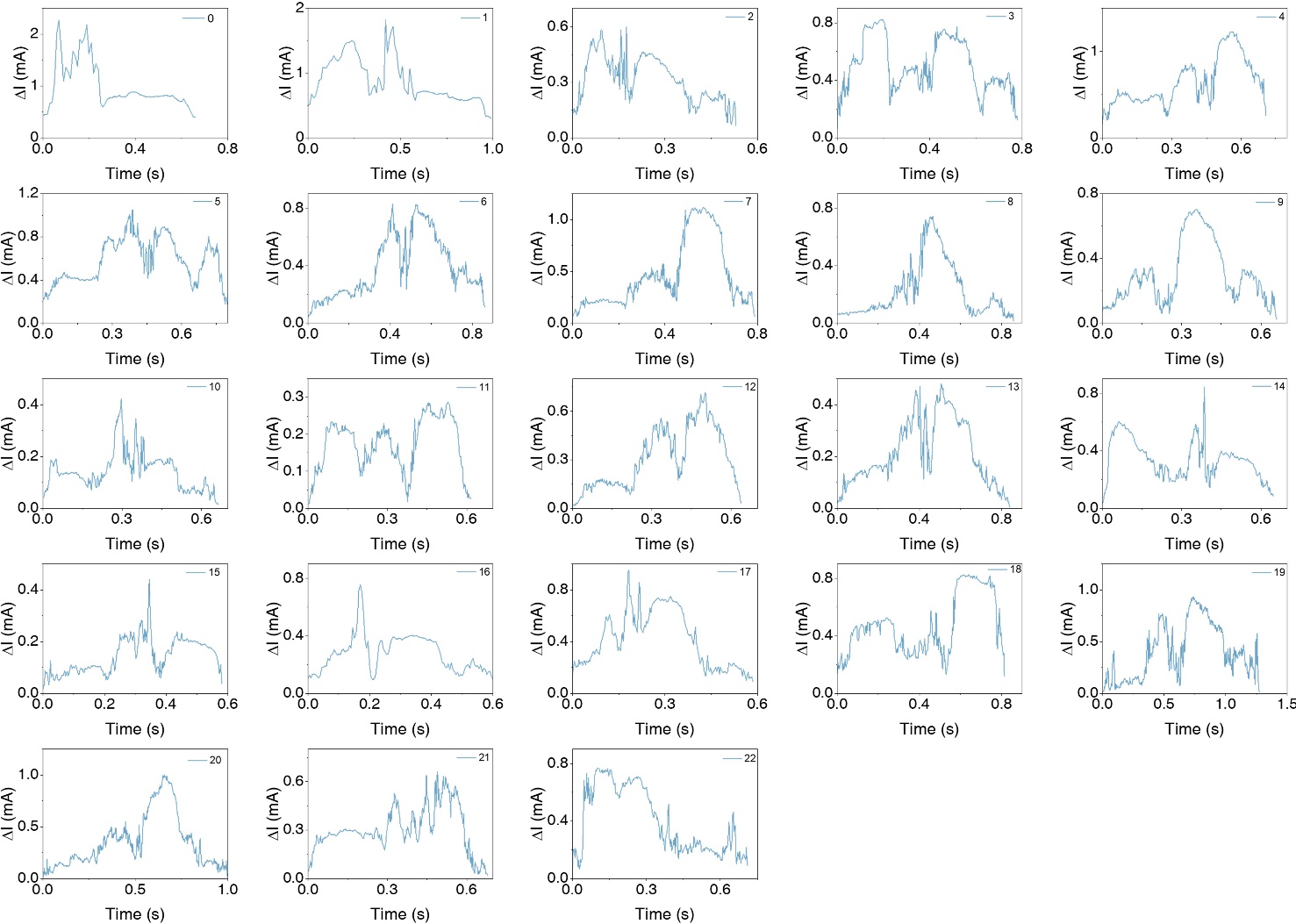


**Figure S32.** Time-domain signals of the bionic sensor corresponding to 23 customized braille under random sliding rates.


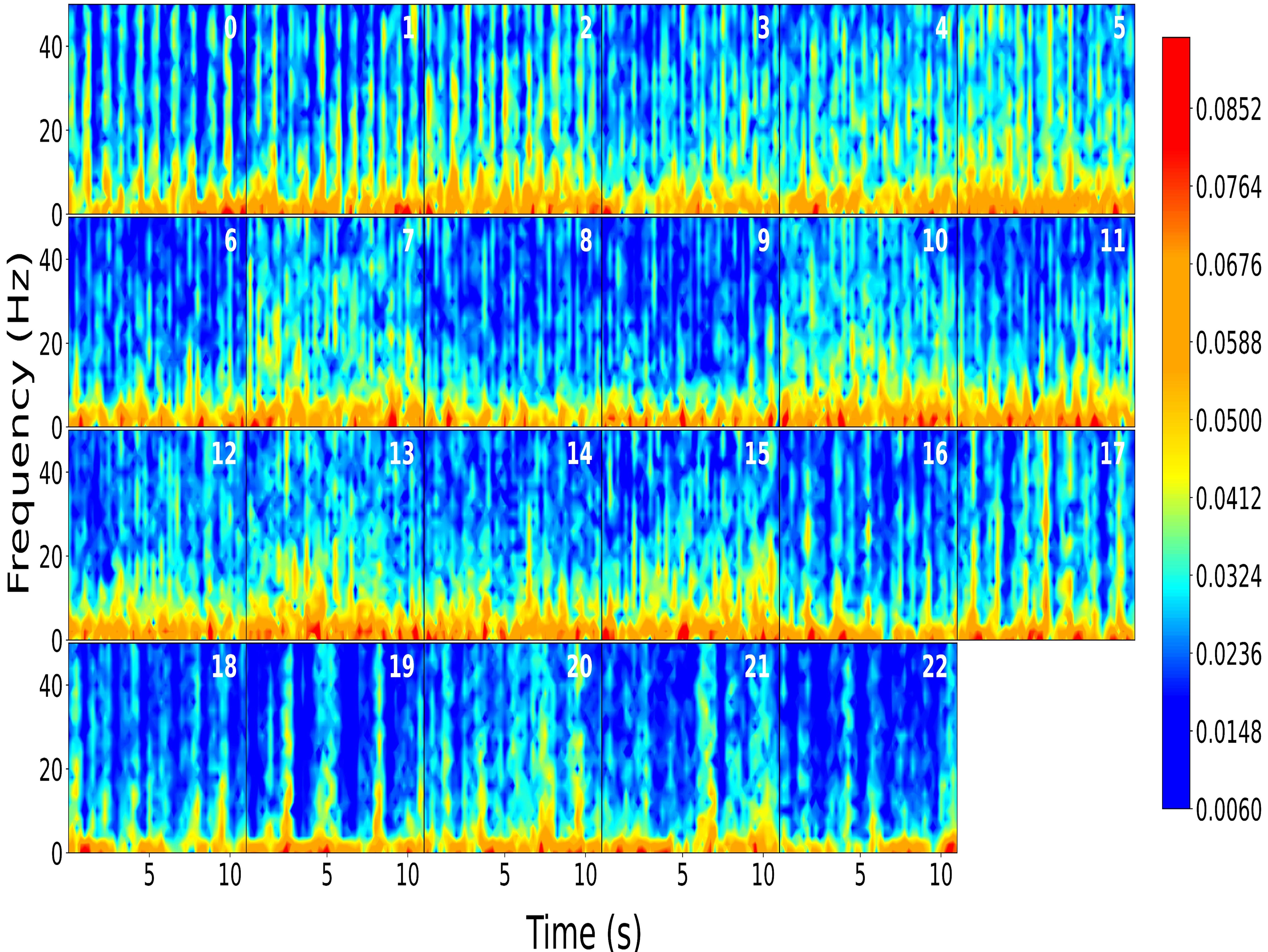


**Figure S33.** Frequency-domain signals of the 23 customized braille obtained using wavelet transform. The sliding rate is random.


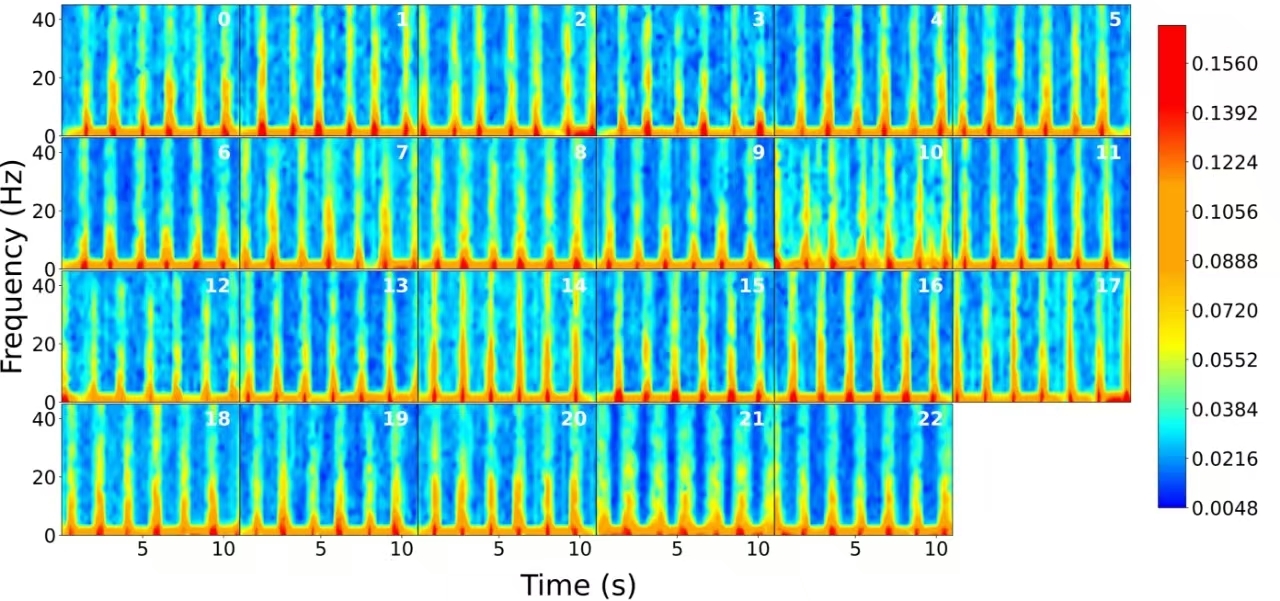


**Figure S34.** Frequency-domain signals of the 23 customized braille obtained using wavelet transform. The sliding rate 150 mm s^-1^.


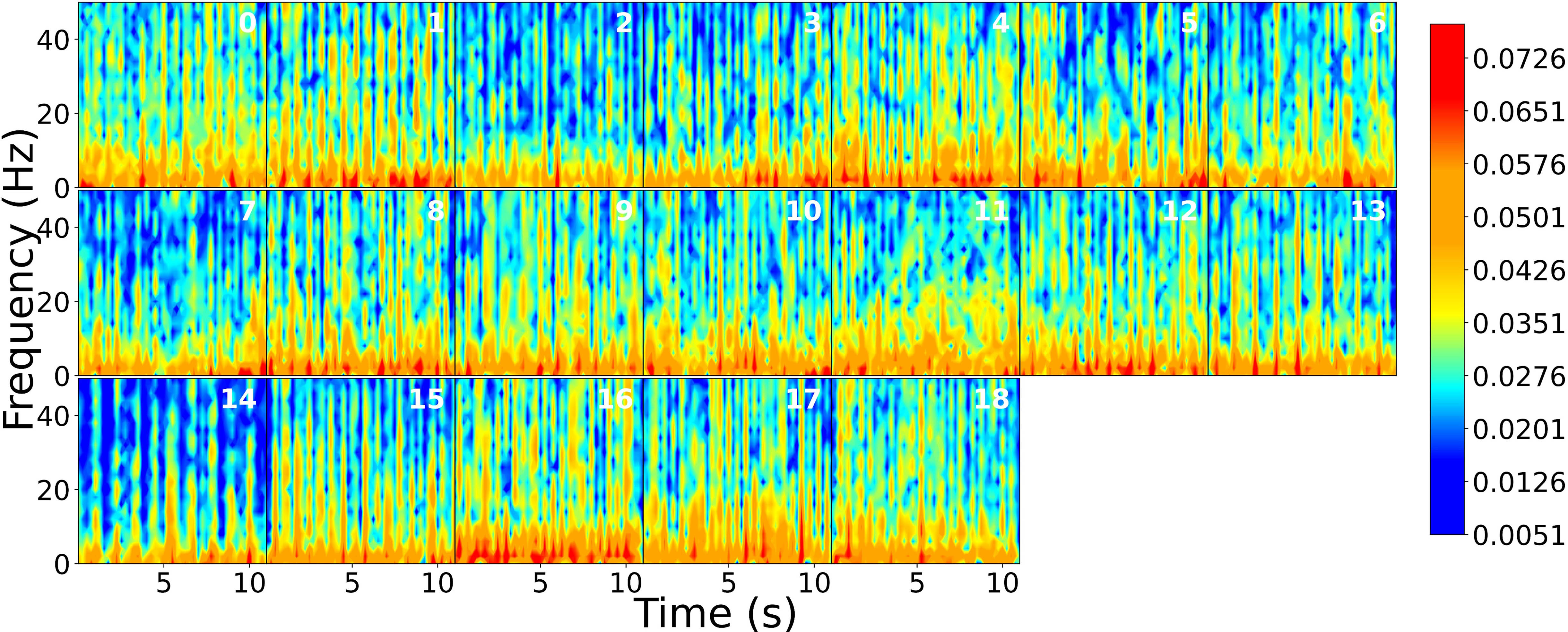


**Figure S35.** Frequency-domain signals of the 19 homemade braille obtained using wavelet transform. The sliding rate is random.


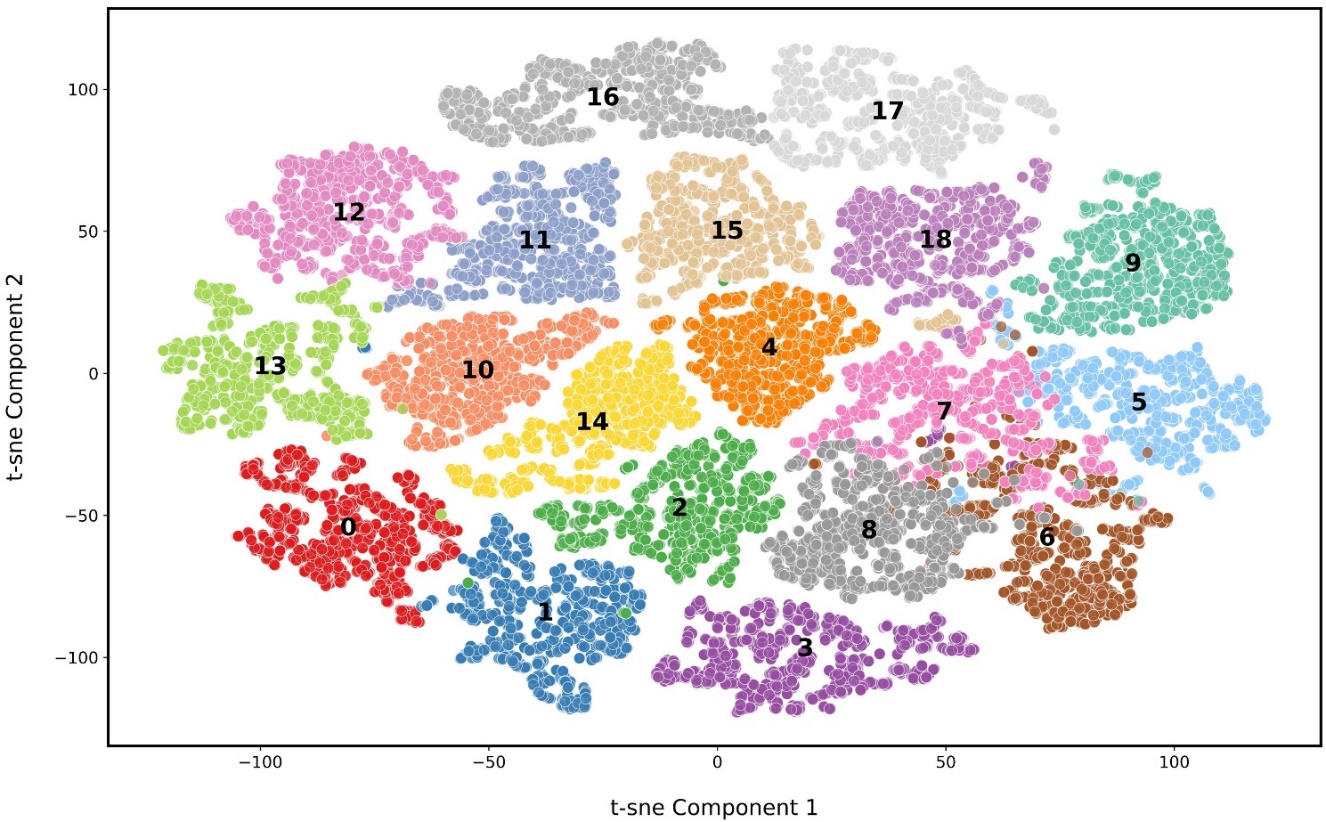


**Figure S36.** t-SNE visualization of the data set collected from the 19 homemade braille by using the sensor attached on human fiber slipping at random speed.


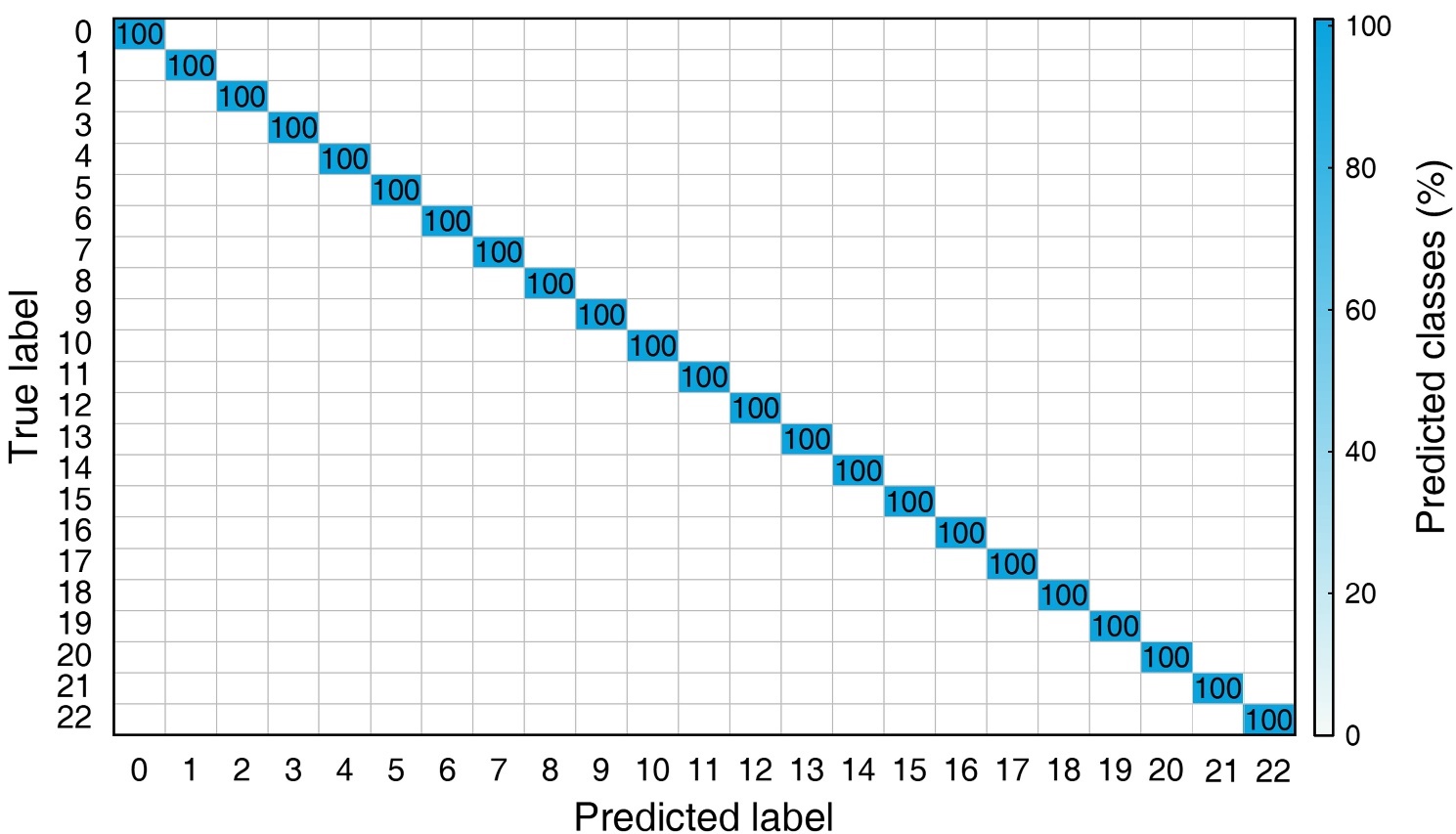


**Figure S37.** The confusion maps in classifying the 23 customized braille at a random sliding speed by attaching the sensor on a human finger.


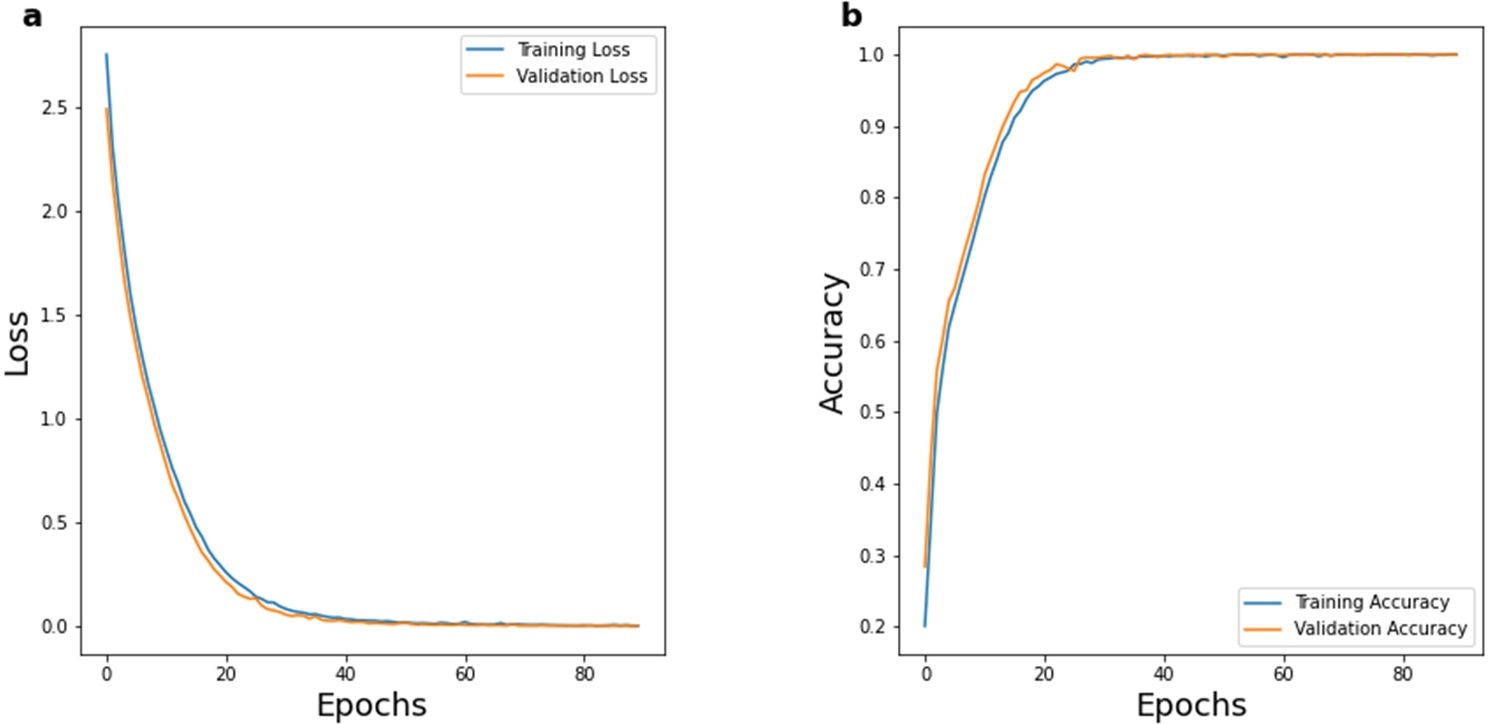


**Figure S38.** Recognition results of 23 customized braille. Training and validation (a) accuracy and (b) loss. The sliding rate is random.


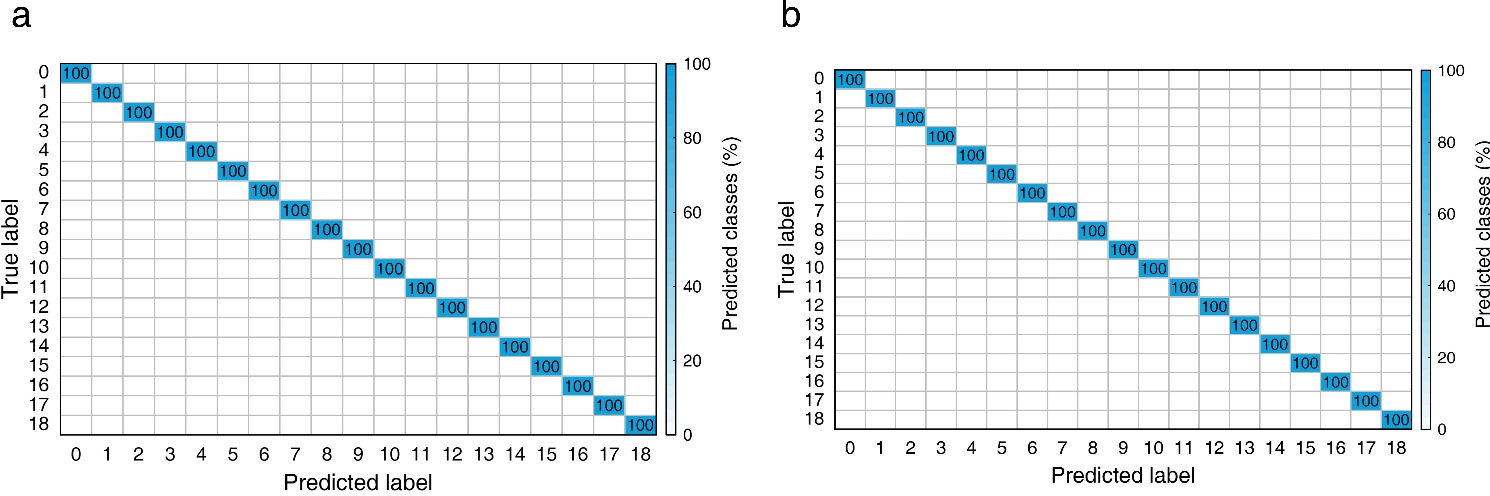


**Figure S39.** Recognition results of 19 homemade braille. The confusion maps for (a) classifying and (b) predicting the 19 homemade braille at a random sliding rate by attaching the sensor on a human finger. Both the training and predict accuracy of 100.0% were achieved.


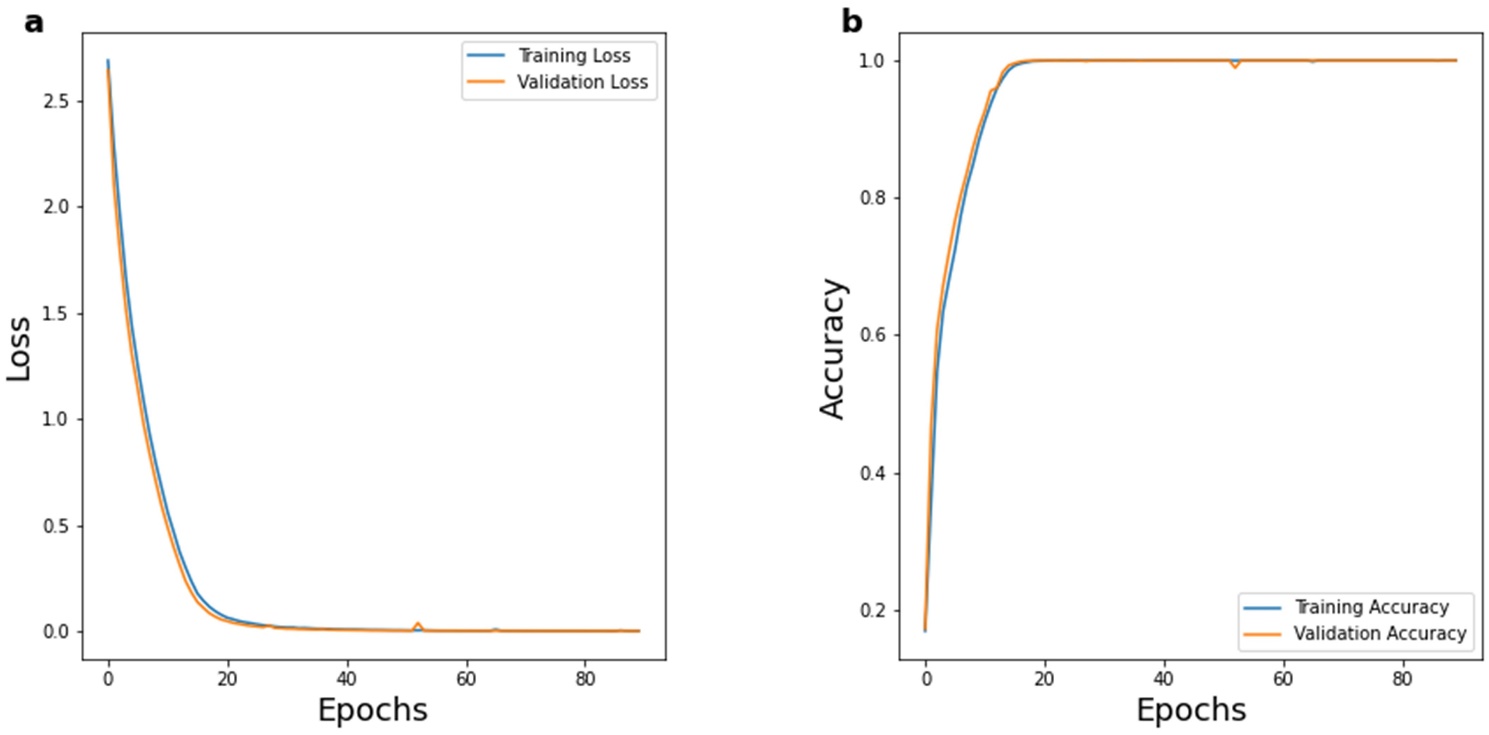


**Figure S40.** Training and validation (a) accuracy and (b) loss towards the 19 homemade braille. The sliding rate is random.

**
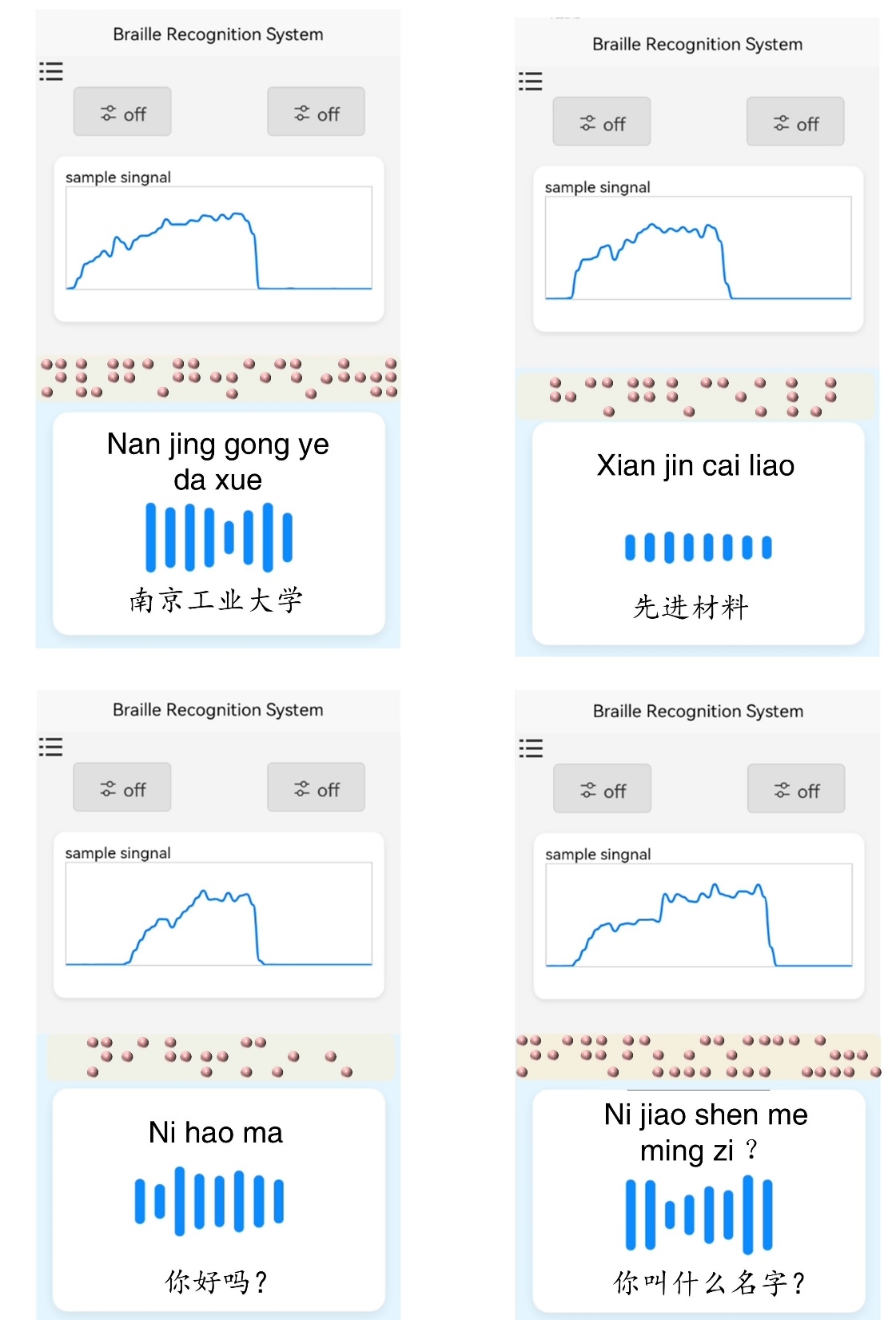
**

**Figure S41.** Demonstrations of the system for wireless recognition of braille sentences


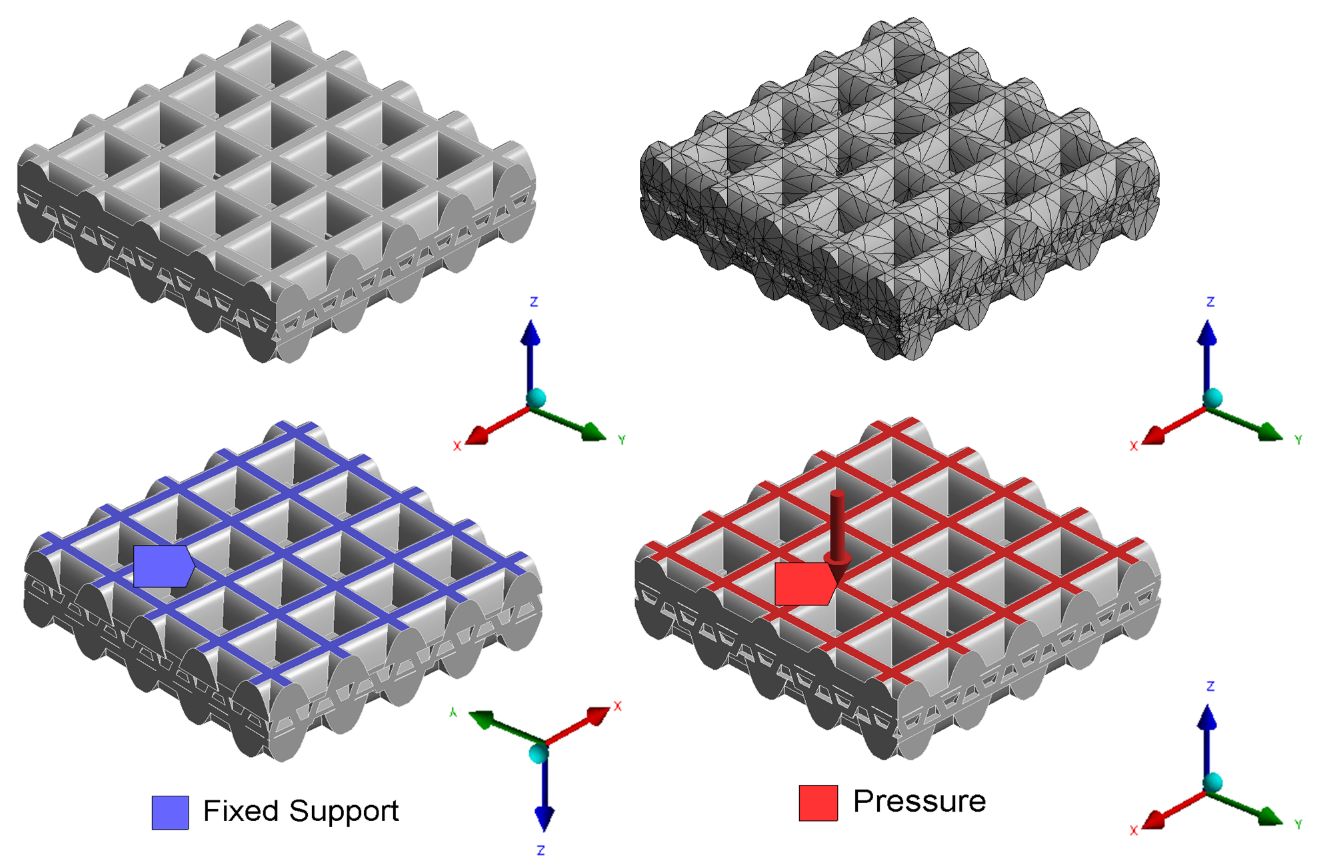


**Figure S42.** FEA modeling of 3D textile-based sensing electrode. (a) Schematic diagram of the fabric structure model. (b) Mesh generation result of the fabric structure. (c) and (d) Loading and constraint conditions of the fabric structure.


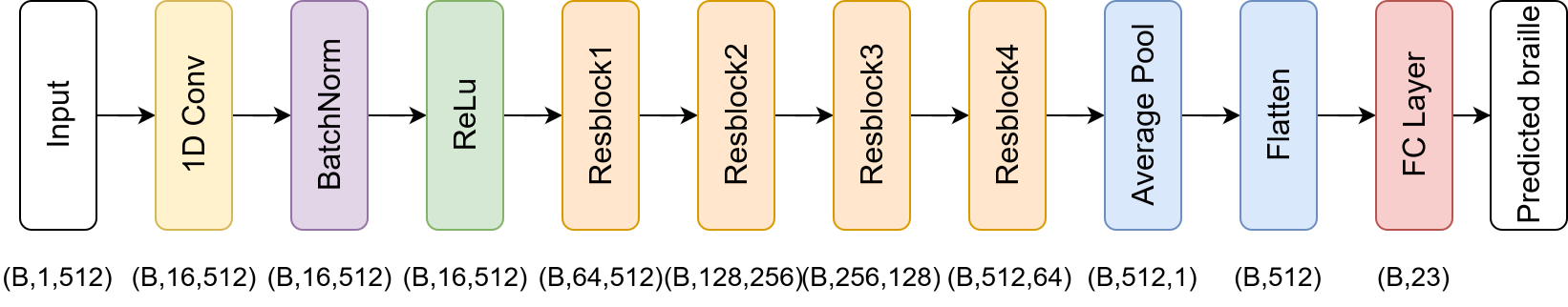


**Figure S43.** The structure of the RSNet model used for braille recognition

**Table S1.** Comparison of performance of TBTS and the other piezoresistive pressure sensors reported in the literature.

| **Electrode**  **type** | **Pressure sensor** | **Response/recovery**  **time (ms)** | **Detection limit (Pa)** | **Sensitivity (kPa^-1^)** | **Ref.** |  |
| --- | --- | --- | --- | --- | --- | --- |
|  | MXene/PEDOT:PSS/honeycomb fabric | **20/30** | **0.12** | **2662.2** (0-10 kPa), **926.1** (10-30 kPa), **460.3** (30-100.5 kPa), **49.5** (100.5-300 kPa) | **This work** |  |
| **Textile-based** | CNT/cotton fabric | 18/24 | 2 | **14.4** (0-3.5 kPa), **7.8** (3.5-15 kPa) | ^[1]^ |  |
|  | Ag NW/Tissue paper | 17/ N/A | 13 | **1.14** (0-5 kPa) | ^[2]^ |  |
|  | CNT/MXene nonwoven fabric | N/A | N/A | **0.245** (0.12-1.9 kPa)**, 0.06** (1.9-12.9 kPa) | ^[3]^ |  |
|  | MXene/woven fabric | 26/50 | N/A | **3.844** (0-29 kPa), **12.095** (29-40 kPa) | ^[4]^ |  |
|  | Graphene/woven fabric | 373/1000 | N/A | **0.16** (0-1 kPa), **0.05** (1-10 kPa) | ^[5]^ |  |
|  | MXene/AgNW nonwoven fabric | 140/30 | 1 | **474.8** (0-1.25 kPa), **72.5** (1.5-5 kPa), **11.9** (7.5-20 kPa), **6** (20-100 kPa) | ^[6]^ |  |
|  | MXene/PVDF nanofiber mat | 10/20 | N/A | **1970.65** (0.025-0.95 kPa), **2.16** (0.95-30 kPa) | ^[7]^ |  |
| **Film-based** | SWNT/PDMS film | 10 | 0.6 | **1.8** (0-0.3 kPa) | ^[8]^ |  |
|  | Au/PDMS film | 20/ N/A | 10.4 | **50.17** (0-0.07 kPa), **1.38** (0.2-1.5 kPa) | ^[9]^ |  |
|  | rGO/PDMS film | 120/80 | 16 | **25.1** (0-2.6 kPa), **0.68** (2.6-40 kPa) | ^[10]^ |  |
|  | rGO/VHB tape | 261/130 | 42 | **47.5** (0-0.1 kPa), **178.5** (0.1-0.2 kPa),  **43.9** (0.2-0.5 kPa), **7.94** (0.5-3 kPa) | ^[11]^ |  |
|  | Gr/PDMS/PET film | 40/30 | 1 | **14** (0-5 kPa), **3.2** (5-12 kPa) | ^[12]^ |  |
|  | PPy/PDMS stamps | 30/40 | 0.88 | **120** (0-0.5 kPa) | ^[13]^ |  |
|  | HPM-PDMS/CNT composite | 170/190 | 0.5 | **83.9** (0-0.14 kPa), **0.4** (0.4-10 kPa) | ^[14]^ |  |
|  | MXene/Au/PET film | 4/13 | 9 | **99.5** (0-1 kPa), **4** (1-4.5 kPa) | ^[15]^ |  |
|  | MXene/PDMS film | 125/104 | 4.4 | **151.4** (0-4.7 kPa), **33.8** (4.7-15 kPa) | ^[16]^ |  |
|  | AuNWs/PDMS film | 10/10 | N/A | **23** (0-0.6 kPa), **0.7** (0.6-3 kPa) | ^[17]^ |  |
|  | MXene/PDMS film | 54/48 | N/A | **461.27** (0-42.8 kPa), **51.01** (42.8-311 kPa) | ^[18]^ |  |
|  | PVANW/carbon paper/PPy film | 66.8/ N/A | 2.97 | **109.9** (0-0.17kPa), **228.5** (0.5-2 kPa),  **11.9** (5-9 kPa) | ^[19]^ |  |
| **Aerogel/sponge-based** | AgNWs/MXene aerogel | 60/114 | 1.25 | **645.7** (0-1 kPa), **50.99** (2-9 kPa) | ^[20]^ |  |
|  | carbon foam | N/A | 3 | **100.29** (0-2 kPa),  **21.22** (2.22-10 kPa) | ^[21]^ |  |
|  | MXene sponge | 138/127 | 9 | **147** (0-5.37 kPa), **442** (5.37-18.56 kPa) | ^[22]^ |  |
|  | CSx/MXene-C aerogels | 109.6/110.6 | 1 | **80.4** (0-5 kPa) | ^[23]^ |  |

**References:**

[1] M. Liu, X. Pu, C. Jiang, T. Liu, X. Huang, L. Chen, C. Du, J. Sun, W. Hu, Z. L. Wang, Large-Area All-Textile Pressure Sensors for Monitoring Human Motion and Physiological Signals, *Adv. Mater.* **2017**, 29, 1703700.

[2] S. Gong, W. Schwalb, Y. Wang, Y. Chen, Y. Tang, J. Si, B. Shirinzadeh, W. Cheng, A Wearable and Highly Sensitive Pressure Sensor with Ultrathin Gold Nanowires, *Nat. Commun.* **2014**, 5, 3132.

[3] X. Zheng, Q. Hu, Z. Wang, W. Nie, P. Wang, C. Li, Roll-to-Roll Layer-by-Layer Assembly Bark-Shaped Carbon Nanotube/Ti_3_C_2_T_x_ MXene Textiles for Wearable Electronics, *J. Colloid Interface Sci.* **2021**, 602, 680.

[4] T. Li, L. Chen, X. Yang, X. Chen, Z. Zhang, T. Zhao, X. Li, J. Zhang, A Flexible Pressure Sensor Based on an MXene–Textile Network Structure, *J. Mater. Chem. C* **2019**, 7, 1022.

[5] Y. Kim, J. Bin Park, Y. J. Kwon, J.-Y. Hong, Y.-P. Jeon, J. U. Lee, Fabrication of Highly Conductive Graphene/Textile Hybrid Electrodes Via Hot Pressing and Their Application as Piezoresistive Pressure Sensors, *J. Mater. Chem. C* **2022**, 10, 9364.

[6] X. Zheng, S. Zhang, M. Zhou, H. Lu, S. Guo, Y. Zhang, C. Li, S. C. Tan, MXene Functionalized, Highly Breathable and Sensitive Pressure Sensors with Multi-Layered Porous Structure, *Adv. Funct. Mater.* **2023**, 33, 2214880.

[7] W. Du, Z. Li, Y. Zhao, X. Zhang, L. Pang, W. Wang, T. Jiang, A. Yu, J. Zhai, Biocompatible and Breathable All-Fiber-Based Piezoresistive Sensor with High Sensitivity for Human Physiological Movements Monitoring, *Chem. Eng. J.* **2022**, 446, 137268.

[8] X. Wang, Y. Gu, Z. Xiong, Z. Cui, T. Zhang, Silk-Molded Flexible, Ultrasensitive, and Highly Stable Electronic Skin for Monitoring Human Physiological Signals, *Adv. Mater.* **2014**, 26, 1336.

[9] B. Su, S. Gong, Z. Ma, L. W. Yap, W. Cheng, Mimosa-Inspired Design of a Flexible Pressure Sensor with Touch Sensitivity, *Small* **2015**, 11, 1886.

[10] Y. Pang, K. Zhang, Z. Yang, S. Jiang, Z. Ju, Y. Li, X. Wang, D. Wang, M. Jian, Y. Zhang, R. Liang, H. Tian, Y. Yang, T.-L. Ren, Epidermis Microstructure Inspired Graphene Pressure Sensor with Random Distributed Spinosum for High Sensitivity and Large Linearity, *ACS Nano* **2018**, 12, 2346.

[11] J. Jia, G. Huang, J. Deng, K. Pan, Skin-Inspired Flexible and High-Sensitivity Pressure Sensors Based on rGO Films with Continuous-Gradient Wrinkles, *Nanoscale* **2019**, 11, 4258.

[12] G. Y. Bae, S. W. Pak, D. Kim, G. Lee, D. H. Kim, Y. Chung, K. Cho, Linearly and Highly Pressure-Sensitive Electronic Skin Based on a Bioinspired Hierarchical Structural Array, *Adv. Mater.* **2016**, 28, 5300.

[13] S. Yu, L. Li, J. Wang, E. Liu, J. Zhao, F. Xu, Y. Cao, C. Lu, Light-Boosting Highly Sensitive Pressure Sensors Based on Bioinspired Multiscale Surface Structures, *Adv. Funct. Mater.* **2020**, 30, 1907091.

[14] T. Zhao, T. Li, L. Chen, L. Yuan, X. Li, J. Zhang, Highly Sensitive Flexible Piezoresistive Pressure Sensor Developed Using Biomimetically Textured Porous Materials, *ACS Appl. Mater. Interfaces* **2019**, 11, 29466.

[15] Y. Gao, C. Yan, H. Huang, T. Yang, G. Tian, D. Xiong, N. Chen, X. Chu, S. Zhong, W. Deng, Y. Fang, W. Yang, Microchannel-Confined MXene Based Flexible Piezoresistive Multifunctional Micro-Force Sensor, *Adv. Funct. Mater.* **2020**, 30, 1909603.

[16] Y. Cheng, Y. Ma, L. Li, M. Zhu, Y. Yue, W. Liu, L. Wang, S. Jia, C. Li, T. Qi, J. Wang, Y. Gao, Bioinspired Microspines for a High-Performance Spray Ti_3_C_2_T_x_ MXene-Based Piezoresistive Sensor, *ACS Nano* **2020**, 14, 2145.

[17] B. Zhu, Y. Ling, L. W. Yap, M. Yang, F. Lin, S. Gong, Y. Wang, T. An, Y. Zhao, W. Cheng, Hierarchically Structured Vertical Gold Nanowire Array-Based Wearable Pressure Sensors for Wireless Health Monitoring, *ACS Appl. Mater. Interfaces* **2019**, 11, 29014.

[18] S. Wang, W. Deng, T. Yang, Y. Ao, H. Zhang, G. Tian, L. Deng, H. Huang, J. Huang, B. Lan, W. Yang, Bioinspired MXene-Based Piezoresistive Sensor with Two-Stage Enhancement for Motion Capture, *Adv. Funct. Mater.* **2023**, 33, 2214503.

[19] C. Luo, N. Liu, H. Zhang, W. Liu, Y. Yue, S. Wang, J. Rao, C. Yang, J. Su, X. Jiang, Y. Gao, A New Approach for Ultrahigh-Performance Piezoresistive Sensor Based on Wrinkled Ppy Film with Electrospun Pva Nanowires as Spacer, *Nano Energy* **2017**, 41, 527.

[20] S. Chen, N. Wu, S. Lin, J. Duan, Z. Xu, Y. Pan, H. Zhang, Z. Xu, L. Huang, B. Hu, J. Zhou, Hierarchical Elastomer Tuned Self-Powered Pressure Sensor for Wearable Multifunctional Cardiovascular Electronics, *Nano Energy* **2020**, 70, 104460.

[21] W. Liu, N. Liu, Y. Yue, J. Rao, C. Luo, H. Zhang, C. Yang, J. Su, Z. Liu, Y. Gao, A Flexible and Highly Sensitive Pressure Sensor Based on Elastic Carbon Foam, *J. Mater. Chem. C* **2018**, 6, 1451.

[22] Y. Yue, N. Liu, W. Liu, M. Li, Y. Ma, C. Luo, S. Wang, J. Rao, X. Hu, J. Su, Z. Zhang, Q. Huang, Y. Gao, 3D Hybrid Porous Mxene-Sponge Network and Its Application in Piezoresistive Sensor, *Nano Energy* **2018**, 50, 79.

[23] Y. Hu, H. Zhuo, Q. Luo, Y. Wu, R. Wen, Z. Chen, L. Liu, L. Zhong, X. Peng, R. Sun, Biomass Polymer-Assisted Fabrication of Aerogels from Mxenes with Ultrahigh Compression Elasticity and Pressure Sensitivity, *J. Mater. Chem. A* **2019**, 7, 10273.

**Table S2.** Sensing performance comparison of the assembled tactile sensor with human skin

| **Sensor** | **Maximum sensing range (kPa)** | | **Detection limit**  **(Pa)** | **Response/recovery**  **time (ms)** |
| --- | --- | --- | --- | --- |
| This work | 300 | 1 | | 20/30 |
| Human skin | 100-300 ^[1]^ | ~1 ^[1]^ | | 20-40 ^[2]^ |

**References:**

[1] K. Cao, M. Wu, J. Bai, Z. Wen, J. Zhang, T. Wang, M. Peng, T. Liu, Z. Jia, Z. Liang, L. Jiang, Beyond Skin Pressure Sensing: 3D Printed Laminated Graphene Pressure Sensing Material Combines Extremely Low Detection Limits with Wide Detection Range, *Adv. Funct. Mater.* **2022**, 32, 2202360.

[2] S. Li, Y. Zhang, Y. Wang, K. Xia, Z. Yin, H. Wang, M. Zhang, X. Liang, H. Lu, M. Zhu, H. Wang, X. Shen, Y. Zhang, Physical Sensors for Skin-Inspired Electronics, *InfoMat* **2020**, 2, 184.

**Table S3.** Comparison of our bionic sensory system with reported sensor systems in terms of acquisition system and recognition accuracy.

| **Tactile sensor type** | **No. of acquisition systems** | **No. of recognized objects** | **Sensitivity** | **Scanning**  **rate**  **(mm s^-1^)** | **Recognition accuracy (%)** | **Ref** |
| --- | --- | --- | --- | --- | --- | --- |
| Piezoresistive +  Triboelectric | Two sets | 12 fabrics | 1.63 kPa^-1^ | 24 | 99.1 | ^[1]^ |
| Triboelectric | One set | 10 braille characters | / | / | 96.12 | ^[2]^ |
| Piezoresistive +  Piezoelectric | Two sets | 20 braille characters | 0.36 V kPa^-1^ | 5~16.98 | 77.5~90.58 | ^[3]^ |
| Piezoresistive | One set | 10 braille characters | 9.8 kPa^-1^ | / | 100.0 | ^[4]^ |
| Piezoresistive | One set | 26 braille characters | 507 kPa^-1^ | / | 99 | ^[5]^ |
| Capacitive | One set | 27 classes of braille letters | / | / | 78.32 | ^[6]^ |
| Piezoelectric | One set | 10 braille characters | 89.11 mV/N | 10 | 96.5 | ^[7]^ |
| Piezoelectric | One set | >25 braille characters | / | / | 90.8 | ^[8]^ |
| Piezoelectric | One set | braille characters | / | / | 88.05~91.45 | ^[9]^ |
| Magnetic | One set | braille characters | / | 15 | 97 | ^[10]^ |
| Piezoresistive | One set | **23 braille characters and 8 sentences** | **2662.2 kPa^-1^** | **10-200** | **100.0** | **This work** |

**References:**

[1] S. Chun, W. Son, H. Kim, S. K. Lim, C. Pang, C. Choi, Self-Powered Pressure- and Vibration-Sensitive Tactile Sensors for Learning Technique-Based Neural Finger Skin, *Nano Lett.* **2019**, 19, 3305.

[2] Y. Lu, D. Kong, G. Yang, R. Wang, G. Pang, H. Luo, H. Yang, K. Xu, Machine Learning-Enabled Tactile Sensor Design for Dynamic Touch Decoding, *Adv. Sci.* **2023**, 10, 2303949.

[3] Z. Gao, L. Chang, B. Ren, J. Han, J. Li, Enhanced Braille Recognition Based on Piezoresistive and Piezoelectric Dual-Mode Tactile Sensors, *Sens. Actuators, A* **2024**, 366, 115000.

[4] X. Liu, L. Fang, F. Zhang, Q. Zhang, Z. Wan, X. Chen, All-Optical Diffractive Deep Neural Networks Enabled Laser-Reduced Graphene Oxide Tactile Sensor for Braille Recognition, *ACS Appl. Electron. Mater.* **2024**, 6, 2049.

[5] X.-F. Zhao, C.-Z. Hang, H.-L. Lu, K. Xu, H. Zhang, F. Yang, R.-G. Ma, J.-C. Wang, D. W. Zhang, A Skin-Like Sensor for Intelligent Braille Recognition, *Nano Energy* **2020**, 68, 104346.

[6] S. F. Muller-Cleve, V. Fra, L. Khacef, A. Pequeno-Zurro, D. Klepatsch, E. Forno, D. G. Ivanovich, S. Rastogi, G. Urgese, F. Zenke, C. Bartolozzi, Braille Letter Reading: A Benchmark for Spatio-Temporal Pattern Recognition on Neuromorphic Hardware, *Front. Neurosci.* **2022**, 16, 951164.

[7] L. Qin, L. Hao, X. Huang, R. Zhang, S. Lu, Z. Wang, J. Liu, Z. Ma, X. Xia, G. Dong, Fingerprint-Inspired Biomimetic Tactile Sensors for the Surface Texture Recognition, *Sens. Actuators, A* **2024**, 371, 115275.

[8] M. Jung, S. Kim, J. Hwang, H. J. Kim, Y. Kim, J. Ahn, S. Jeon, Flexible Artificial Mechanoreceptor Based on Microwave Annealed Morphotropic Phase Boundary of Hfxzr1-Xo2 Thin Film, *Adv. Electron. Mater.* **2024**, 10, 2300594.

[9] M. Tanaka, K. Miyata, S. Chonan, A Wearable Braille Sensor System with a Post Processing, *IEEE/ASME Trans. Mechatron.* **2007**, 12, 430.

[10] Y. Yan, Z. Hu, Y. Shen, J. Pan, Surface Texture Recognition by Deep Learning-Enhanced Tactile Sensing, *Adv. Intell. Syst.* **2022**, 4, 2100076.

**Table S4.** Comparison of our bionic sensory with reported multi-mode sensors

| **Tactile sensor type** | **No. of acquisition systems** | **Frequency**  **response range**  **（Hz）** | **Signal integrity**  **(peaks s⁻¹ Hz⁻¹)** | **Signal crosstalk** | **Ref.** |
| --- | --- | --- | --- | --- | --- |
| Potentiometric + triboelectric | Two sets | 0.067-10 | / | Yes | ^[1]^ |
| Piezoresistive + piezoelectric | Two sets | 1-1000 | 1 | Yes | ^[2]^ |
| Piezoresistive +thermoelectric  + triboelectric | Three sets | 0.1-8 | 1 | Yes | ^[3]^ |
| Piezoresistive + triboelectric | Two sets | 0.9-2 | 1 | Yes | ^[4]^ |
| Piezoresistive + triboelectric | Two sets | 1-4 | 1 | Yes | ^[5]^ |
| Piezoresistive + triboelectric | Two sets | 0-200 | 1 | Yes | ^[6]^ |
| Piezoresistive | One set | 5-600 | 1 | No | This work |

**References:**

[1] X. Wu, J. Zhu, J. W. Evans, A. C. Arias, A Single-Mode, Self-Adapting, and Self-Powered Mechanoreceptor Based on a Potentiometric–Triboelectric Hybridized Sensing Mechanism for Resolving Complex Stimuli, *Adv. Mater.* **2020**, 32, 2005970.

[2] S. Chun, J.-S. Kim, Y. Yoo, Y. Choi, S. J. Jung, D. Jang, G. Lee, K.-I. Song, K. S. Nam, I. Youn, D. Son, C. Pang, Y. Jeong, H. Jung, Y.-J. Kim, B.-D. Choi, J. Kim, S.-P. Kim, W. Park, S. Park, An Artificial Neural Tactile Sensing System, *Nat. Electron.* **2021**, 4, 429.

[3] Y. Wang, H. Wu, L. Xu, H. Zhang, Y. Yang, Z. L. Wang, Hierarchically Patterned Self-Powered Sensors for Multifunctional Tactile Sensing, *Sci. Adv.* **2020**, 6, eabb9083.

[4] H. Zhang, H. Li, Y. Li, Biomimetic Electronic Skin for Robots Aiming at Superior Dynamic-Static Perception and Material Cognition Based on Triboelectric-Piezoresistive Effects, *Nano Lett.* **2024**, 24, 4002.

[5] X. Wei, H. Li, W. Yue, S. Gao, Z. Chen, Y. Li, G. Shen, A High-Accuracy, Real-Time, Intelligent Material Perception System with a Machine-Learning-Motivated Pressure-Sensitive Electronic Skin, *Matter* **2022**, 5, 1481.

[6] S. Chun, W. Son, H. Kim, S. K. Lim, C. Pang, C. Choi, Self-Powered Pressure- and Vibration-Sensitive Tactile Sensors for Learning Technique-Based Neural Finger Skin, *Nano Lett.* **2019**, 19, 3305.
